# NSP4 and ORF9b of SARS-CoV-2 Induce Pro-Inflammatory Mitochondrial DNA Release in Inner Membrane-Derived Vesicles

**DOI:** 10.3390/cells11192969

**Published:** 2022-09-23

**Authors:** Md Imam Faizan, Rituparna Chaudhuri, Shakti Sagar, Sarah Albogami, Nisha Chaudhary, Iqbal Azmi, Areej Akhtar, Syed Mansoor Ali, Rohit Kumar, Jawed Iqbal, Mohan C. Joshi, Gaurav Kharya, Pankaj Seth, Soumya Sinha Roy, Tanveer Ahmad

**Affiliations:** 1 Multidisciplinary Centre for Advanced Research & Studies (MCARS), Jamia Millia Islamia, New Delhi 110025, India; 2 Molecular and Cellular Neuroscience, Neurovirology Section, National Brain Research Centre (NBRC), Gurugram 122052, India; 3 CSIR-Institute of Genomics and Integrative Biology, New Delhi 110007, India; 4 Department of Biotechnology, College of Science, Taif University, P.O. Box 11099, Taif 21944, Saudi Arabia; 5 Department of Biotechnology, Jamia Millia Islamia, New Delhi 110025, India; 6 Department of Pulmonary Medicine and Sleep Disorders, Vardhman Mahavir Medical College, Safdarjung Hospital, New Delhi 10029, India; 7 Center for Bone Marrow Transplantation & Cellular Therapy Indraprastha Apollo Hospital, New Delhi 110076, India

**Keywords:** mitochondrial DNA, SARS-CoV-2, NSP4, ORF9b, myeloid cell leukemia-1, BCL2 antagonist/killer, tunneling nanotubes, intercellular mitochondrial transfer

## Abstract

Circulating cell-free mitochondrial DNA (cf-mtDNA) has been found in the plasma of severely ill COVID-19 patients and is now known as a strong predictor of mortality. However, the underlying mechanism of mtDNA release is unexplored. Here, we show a novel mechanism of SARS-CoV-2-mediated pro-inflammatory/pro-apoptotic mtDNA release and a rational therapeutic stem cell-based approach to mitigate these effects. We systematically screened the effects of 29 SARS-CoV-2 proteins on mitochondrial damage and cell death and found that NSP4 and ORF9b caused extensive mitochondrial structural changes, outer membrane macropore formation, and the release of inner membrane vesicles loaded with mtDNA. The macropore-forming ability of NSP4 was mediated through its interaction with BCL2 antagonist/killer (BAK), whereas ORF9b was found to inhibit the anti-apoptotic member of the BCL2 family protein myeloid cell leukemia-1 (MCL1) and induce inner membrane vesicle formation containing mtDNA. Knockdown of BAK and/or overexpression of MCL1 significantly reversed SARS-CoV-2-mediated mitochondrial damage. Therapeutically, we engineered human mesenchymal stem cells (MSCs) with a simultaneous knockdown of BAK and overexpression of MCL1 (MSCshBAK+MCL1) and named these cells IMAT-MSCs (intercellular mitochondrial transfer-assisted therapeutic MSCs). Upon co-culture with SARS-CoV-2-infected or NSP4/ORF9b-transduced airway epithelial cells, IMAT-MSCs displayed functional intercellular mitochondrial transfer (IMT) via tunneling nanotubes (TNTs). The mitochondrial donation by IMAT-MSCs attenuated the pro-inflammatory and pro-apoptotic mtDNA release from co-cultured epithelial cells. Our findings thus provide a new mechanistic basis for SARS-CoV-2-induced cell death and a novel therapeutic approach to engineering MSCs for the treatment of COVID-19.

## 1. Introduction

Severe acute respiratory syndrome coronavirus 2 (SARS-CoV-2) can directly or indirectly induce airway epithelial cell death [1,2,3]. In direct virus-induced cell death, viral proteins hijack the host cell’s anti-apoptotic proteins and subsequently activate pro-apoptotic signaling. Meanwhile, activated secondary molecules, such as damage-associated molecular patterns (DAMPs) and secreted pro-inflammatory cytokines (PICs), predominantly mediate the indirect mechanism of virus-induced cell death [4,5]. Among the PICs, TNF-α and IFN-γ promote inflammatory cell death [6,7] leading to a cytokine storm, which is a major cause of COVID-19-associated mortality [8,9]. In addition to PICs, DAMPs such as mtDNA promote cell death in inflammatory and infectious diseases [10,11]. The upregulated plasma mtDNA levels in patients with COVID-19 are positively correlated with the pro-inflammatory markers [12,13]. Circulating mtDNA was suggested to be an indicator of COVID-19 severity. The correlation of circulating mtDNA with COVID-19 severity is higher than that of other PICs [12,14]. Genetic studies have identified mtDNA variants in patients with COVID-19 that can predict disease severity or resistance and the risk of developing severe COVID-19 [15,16]. In addition to serving as a potential biomarker, extramitochondrial mtDNA induces a PIC response and chronic inflammation [11]. The release of mtDNA is generally preceded by extensive mitochondrial structural and functional changes, which activate the mitochondrial membrane pore-forming complexes [10].

The mitochondria are at the intersection of key pathways regulating antiviral signaling, the pro-inflammatory response, and cell death [17]. The outer mitochondrial membrane protein MAVS (mitochondrial antiviral-signaling protein) is a critical determinant of anti-viral signaling [18,19]. After viral infection, the viral nucleic acids activate the MAVS-dependent anti-viral innate immune responses. Recent studies have suggested that SARS-CoV-2 infection impairs MAVS-mediated anti-viral signaling [20,21]. In particular, ORF10b induces the autophagy-dependent degradation of MAVS, which limits the mitochondrial content available for participating in anti-viral signaling. Co-immunoprecipitation analysis has revealed that several SARS-CoV-2 proteins interact with the components of mitochondrial permeability transition pore (mPTP) to induce autophagy and mitochondrial clearance [22]. These effects of SARS-CoV-2 are mediated by direct interaction with the key mitochondrial proteins involved in mitochondrial calcium cycling in cardiomyocytes. Conversely, SARS-CoV-2 infection is reported to impair mitophagy [23], suggesting that the mitochondria are essential for SARS-CoV-2 replication.

Studies using apoptotic inducers have demonstrated that mtDNA release is mediated by a regulated pathway. Seminal work from the laboratory of Benjamin Kile and Stephen Tait has demonstrated that BAK/BAX macropores formed on the outer membrane provide a passage for mtDNA extrusion [24,25]. The inhibition of anti-apoptotic signaling facilitated macropore formation. The BAX/BAK-dependent mtDNA release was not dependent on mPTP formation, suggesting that the formation of the BAX/BAK outer membrane macropore specifically regulates the mtDNA extrusion pathway [25]. Recent studies have also demonstrated that mtDNA is released through the BAX/BAK macropore during viral infections [26] and inflammatory conditions [27]. However, the mechanism underlying mtDNA extrusion from the inner membrane has not been elucidated and is speculated to be a passive event. During this event, macropore formation facilitates inner membrane herniation, which subsequently undergoes dissolution upon direct exposure to the cytosol. Subsequently, the damaged mitochondria promote the release of mtDNA into the cytosol or the extracellular space. In the cytosol, mtDNA activates the classical cGAS/STING1-mediated interferon signaling and NLRP3 inflammasome formation. Extracellular mtDNA is a potential driver of the pro-inflammatory response [25,28,29,30]. SARS-CoV-2 infection is associated with extensive mitochondrial damage [31], high extracellular mtDNA levels [12], and impaired anti-viral signaling [32].

Mesenchymal stem cells (MSCs) have exhibited clinical success in the treatment of various inflammatory diseases [33,34]. According to a recently elucidated mechanism, the therapeutic efficacy of MSCs can be attributed to their unique ability to donate mitochondria through tunneling nanotubes (TNTs) and extracellular vesicles [35,36]. Direct mitochondrial transplantation can also alleviate pathological conditions [37,38]. Recent studies have reported that bone marrow and T-cell-mediated intercellular mitochondrial transfer (IMT) facilitate the survival of cancer cells [39,40]. Thus, IMT is emerging as a new paradigm through which mammalian cells, especially MSCs, exert their therapeutic effects. In this study, we found that SARS-CoV-2 and its encoded proteins NSP4 and ORF9b synergistically induced outer membrane macropore formation and inner membrane vesicle formation to facilitate extracellular mtDNA release. The released mtDNA induced a pro-inflammatory response in the target airway epithelial cells, leading to cell death. To design effective therapeutic strategies, MSCs were genetically engineered to harbor macropore-deficient mitochondria by simultaneously downregulating BAK and overexpressing MCL1. The donation of the engineered mitochondria by MSCs to SARS-CoV-2-infected or NSP4-ORF9b-expressing airway epithelial cells mitigated SARS-CoV-2-induced pro-inflammatory mtDNA release and rescued airway epithelial cell death.

## 2. Material and Methods

### 2.1. Ethical Clearance and Patient Information

Blood samples from the patients used in the present study were obtained from the Safdarjung Hospital. The blood samples were collected in vacutainers containing ethylenediaminetetraacetic acid (EDTA) (POLYMED Medical Devices, India). Human ethical clearance (IEC/VMMC/SJH/Project/2020-07/CC-06) was obtained for blood collection. Further, institutional ethical clearance (1/10/290/JMI/IEC/2020) and biosafety clearance (Ref. No. P1/12–21.12.2020) were also obtained from Jamia Millia Islamia as mentioned by us previously [41]. Patient consent was obtained while collecting the samples according to the ICMR GCP guidelines.

### 2.2. Sample Collection and Processing

In total, 162 blood samples were collected for the study by a trained clinician from Safdarjung hospital, New Delhi. Samples collected between January 2022 and March 2022 are referred to as OmicronS (Omicron-suspected). Similarly, as described by us previously [41], samples obtained between June 2020 and October 2020 are referred to as COVID-19. The collected samples included 89 COVID-19-positive, 35 COVID-19-negative, 14 convalescent patients (recovered with confirmed negative RT-qPCR results), and 24 new cases of OmicronS. The collection of the blood samples was conducted along with the swab and saliva samples, which were used for the RT-qPCR-based detection of COVID-19 by the hospital and further confirmed by us as mentioned earlier [41]. The processing of the samples was carried out in a NABL-certified (MC-3486) and ICMR-approved diagnostic laboratory for COVID-19 testing following regulatory guidelines and protocols as described previously [41]. Blood samples were processed to obtain the plasma by centrifuging at 2000× *g* for 15 m, and the processed samples were either used immediately or stored at −80 °C for future use.

### 2.3. RT-qPCR and PCR Analysis

Saliva samples from patients were collected and processed as previously described [41]. To check for the Omicron variant, a PCR assay was performed with S gene target failure (SGTF), as per the published report [42]. The *IL6* and *IFNB* RT-qPCR were performed using the RNA extracted (QIAGEN RNA extraction kit) from NHBE cells. The RNA was subjected to cDNA synthesis (QIAGEN RNA extraction kit) followed by PCR using KAPA SYBR FAST qPCR master mix (KAPA). The assay was performed using Rotor-Gene Q (QIAGEN).

The mitochondrial DNA copy number was determined in the patient plasma samples using 50 μL as the starting sample. DNA was extracted in 20 μL of TE buffer (Vivantis Technologies, Malaysia). Finally, 2 μL of DNA was used per sample of PCR reaction containing the primers spanning various regions of the mitochondrial genome as shown in Supplementary Table. PCR was performed using 2X PCR Master Mix (Thermo Fisher Scientific, Boston, MA, USA) or amaR OnePCR (GeneDireX, Inc., Vegas, NV, USA). The mtDNA copy number was determined as previously described [43]. Similarly, the mtDNA copy number was determined in the chromatin-immunoprecipitated plasma samples (described later in this section). The amplicon of the representative extracted mtDNA (ND2) was also confirmed by performing gel-electrophoresis as described by us previously [41].

### 2.4. Cell Culture and Transfection

A549 cells (adenocarcinoma human alveolar basal epithelial cells) were obtained from ATCC and cultured in Ham’s F12 medium (Sigma) supplemented with 10% heat-inactivated FBS (Gibco), 2 mM L-glutamine (Invitrogen), and 100 U/mL penicillin-streptomycin (Invitrogen). Cells were maintained in a 5% CO_2_ incubator at 37 °C. Transfection was carried out using lipofectamine 3000 (Thermo Fisher Scientific, Boston, MA, USA) according to the manufacturer’s protocols. Briefly, 30,000 cells were seeded in 24-well plates on the coverslip for immunofluorescence. After 48 h at 70% confluency, the cells were transfected with the respective plasmid’s master mix, which included 250 µL of reduced serum medium Opti-MEM (Thermo Fisher Scientific, Boston, MA, USA), 1 µg plasmid, and 2 µg transfection reagent. Cells were incubated for 6 h in the transfection mix, followed by the removal of the transfection complexes and the addition of fresh media. After 24 h, the cells were processed for immunoblotting or immunofluorescence. Normal Human Bronchial Epithelial (NHBE) cells (Lonza Bioscience) were maintained in a 5% CO_2_ incubator at 37 °C. The cells were grown in BEGM™-2 BulletKit medium (Lonza Bioscience). The medium was changed every 48 h.

### 2.5. Human Mesenchymal Stem Cell Isolation, Culture, and Characterization

Human bone marrow samples were obtained from healthy individuals with no history of infectious disease, diabetes, or metabolic syndrome at the Apollo Indraprastha Hospital, New Delhi, after obtaining proper patient consent. MSCs were isolated from the collected bone marrow as described by us previously [35]. Stem cell marker analyses were performed using flow cytometry. Briefly, cells were fixed with 4% paraformaldehyde (PFA) (Sigma) and incubated in a blocking solution (1.5% normal goat serum and 3% BSA) for 30 min, followed by staining with the respective primary fluorophore-conjugated antibodies for 1 h. CD44-FITC, CD73-PE, and CD90-PE were used as positive markers, and CD45-FITC was used as a negative marker. Analysis was performed by acquiring 10,000 events, and the samples were run in triplicate using a BD FACSMelody instrument. FlowJo™ v.10 software (BD Biosciences, East Rutherford, NJ, USA) was used for analysis.

### 2.6. DNA Constructs and Plasmids

All SARS-CoV-2 protein-encoding constructs were obtained as kind gifts from Dr. Nevan Krogan [44]. An empty vector was generated by deleting the open reading frame of NSP4 using the restriction enzymes EcoRI and BamH1 (NEB) while leaving the Strep II tag intact. BAK, BAX, and MCL1 shRNA were generated using the pLKO.1 vector backbone (Addgene#269279). The scrambled sequence was replaced with respective shRNA sequences as shown in Supplementary Table. The shRNA sequences for BAK and BAX were obtained as synthetic fragments; PCR was amplified to generate the double strands and then cloned into the vectors using the NdeI and EcoRI (NEB) restriction sites. The sequences were generated using the online (Custom Dicer-Substrate siRNA (DsiRNA)) tool from Integrated DNA Technologies. MCL1 was obtained as a double-stranded gene fragment with the corresponding NdeI and EcoRI restriction sites and introduced into the pLKO.1 vector. Three different shRNA target sequences were tested for BAK and BAX and two for MCL1.

The lentiviral-encoded MCL1 vector was obtained as a kind gift from Scott J Dixon [45]. The Bcl-xL construct used in this study was generated using pLYS1-FLAG-MitoGFP-HA (Addgene#50057) and the ORF of the Bcl-xL plasmid (Addgene #140749) obtained from Scott J Dixon. The ORF of the Bcl-xL was obtained by PCR amplification with a forward primer containing Nhe1 and a reverse primer containing the BsrGI sites. Both the PCR-amplified products and the pLYS1-FLAG-MitoGFP-HA vector (referred to as mitoGFP) were digested followed by ligation. The final vector obtained was named pLV-Bcl-xL after sequence confirmation by restriction and sequencing. Similarly, lentiviral-encoded PINK1 was generated by PCR amplification of the PINK1 ORF from the pEYFP-N1-Pink1 (Addgene #101874) with the Nhe1 and BsrGI (NEB) sequences in the primers. The amplified product was inserted into the mitoGFP. The final plasmid lentiviral-encoded PINK1 plasmid was confirmed by sequencing and named pLV-PINK1.

### 2.7. Preparation of Lentiviral Particles

To produce lentiviruses, the plasmid-encoding gene of interest (transfer plasmid) was co-transfected with the packaging vector plasmid (pCMV-dR8.2 dvpr; Addgene #8455) and envelope-encoding plasmid (pCMV-VSV-G; Addgene # 8454) in HEK 293T cells at a ratio of 4:4:2, respectively (total 10 µg of DNA), in T25 flasks. Cells were transfected using 20 µL of LTX (Invitrogen) and 5 ml of complete DMEM media (Gibco). After 48 h of transfection, the media containing the lentiviral particles was collected and centrifuged at 500× *g* for 10min. Then, the clarified supernatant was incubated with Lenti-X (Lenti-X™ Concentrator, Takara, SHG, Japan) in a 3:1 ratio. This supernatant–LentiX mixture was incubated for 48 h at 4 °C. After 48 h, the mixture was centrifuged at 1500× *g* for 45 min at 4 °C. The concentrated pellet was resuspended in 100 µL of complete media and used immediately at MOI of 5 or stored at −80 °C as aliquots. The MOI was calculated by real-time quantitative PCR using a lentivirus titration kit (Lenti-X RT-qPCR Titration Kit, Takara, SHG, Japan). All transduction experiments in A549 cells with various SARS-CoV-2-encoded plasmids or plasmids encoding shRNA for BAK, BAX, and MCL1 were carried out at MOI 5 for 48 h or 72 h unless specified. Similarly, for the generation of genetically engineered MSC^shBAK+MCL1^, cells were transduced with the respective lentiviral particles for 48 h before the co-culture experiment. To evaluate the expression of BAK or MCL1 in MSCs, the cells were fixed, followed by immunofluorescence with the respective antibodies.

### 2.8. SYTOX GREEN Assay

Flow cytometry analysis was performed to measure cell death by staining the cells with SYTOX Green Nucleic Acid Stain (Thermo Fisher Scientific, Boston, MA, USA). The assay was performed according to the manufacturer’s instructions. Briefly, cells were stained with the dye for 20 min and washed before acquisition. About 10,000 cells were acquired per sample and the samples were run in triplicate. Flow cytometry analysis was performed using a BD Accuri C6 Plus, and the data were analyzed using FlowJo™ v.10 Software.

### 2.9. TUNEL Assay

TUNEL assays were performed by us as described previously and following the manufacturer’s instructions (Promega) [35]. NHBE cells were first stained with CellTracker Green (CTG) before co-culture for identification. After 48 h, the media were replaced with fresh media containing SARS-CoV-2 viruses at MOI of 0.2. After 48 h of transduction, the virus-containing medium was removed and the cells were washed with 1× PBS four times and fixed with 4% PFA. The fixed cells were used for further immunofluorescence analysis. All infections were performed in the BSL3 facility at the Regional Centre for Biotechnology, Faridabad, Haryana.

### 2.10. SARS-CoV-2 Infection Model

A549 cells were transduced with lentiviral particles derived from the ACE2/TMPRSS2-expressing vector (Addgene #154987), and cells stably expressing ACE2/TMPRSS2 were selected using puromycin selection. Around 30,000 ACE2/TMPRSS2-expressing A549 cells were seeded on coverslips in 24-well plates. After 48 h, the media were replaced with fresh media containing SARS-CoV-2 viruses at MOI of 0.2. After 48 h of transduction, the virus-containing media were removed and the cells were washed with 1× PBS four times then fixed with 4% PFA. The fixed cells were used further to perform immunofluorescence. All the infections were carried out in the BSL3 facility at the Regional Centre for Biotechnology, Faridabad, Haryana.

### 2.11. Flow Cytometry Analysis

As described by us previously [46], the mitochondrial reactive oxygen species (mtROS) were measured by staining the cells with MitoSOX Red (Thermo Fisher Scientific, Boston, MA, USA) and the mitochondrial membrane potential (ΔΨm) was measured with TMRE (Sigma-Aldrich, St. Louis, MO, USA); 50,000 cells/well were seeded in 24-well plates. After 24 h of seeding, the cells were transfected with different plasmid constructs in triplicate and allowed to grow for another 24 h. The cells were then washed using 1× PBS, stained with MitoSOX or TMRE as per the recommended concentrations, and incubated for 15 min, followed by washing and analysis by flow cytometry using a BD Accuri C6 plus or BD Melody, respectively. Similarly, cells were stained with Mito-Tracker Green for 20 min and analyzed using a BD Accuri C6. Ten thousand events were acquired for each experiment and the data were analyzed using FlowJo™ v.10 Software.

### 2.12. Drug Treatments

To induce mitochondrial DNA release from A549 cells, 30,000 cells were seeded in 24-well plates with coverslips. After 48 h of seeding, cells were transduced/transfected with mitoGFP lentivirus/respective plasmids. After 24 h of transduction/transfection, one set of cells was treated with a combination of three different drugs, namely, ABT737 (10 µM, Calbiochem, CA, USA), Actinomycin D (1 µM, Calbiochem), and zVAD (20 µM, Sigma-Aldrich, St. Louis, Mo, USA) for 2 h and the other set of the cells was used as a control. The treated cells were processed for immunofluorescence.

Similarly, cells were treated with Antimycin A at a concentration of 100 nM (Sigma) for 24 h to induce mtROS and FCCP at 10 µM for 4 h to induce a decrease in ΔΨm. For the co-culture experiments, A549 cells were treated with rotenone at 100 nM for 24 h before co-culture with MSCs.

### 2.13. Transmission Electron Microscopy

TEM imaging was performed to investigate the ultrastructural changes in the mitochondria. Briefly, lentivirus-transduced A549 cells were fixed in 4% PFA and 2.5% glutaraldehyde. Excess fixative was removed using a 0.1M sodium cacodylate buffer and processed for block preparation in 2% agar. Another round of fixation was conducted in 2% osmium tetra oxide for 1 hr. Dehydration of the samples was carried out using 30%, 50%, 70%, and 100% ethanol, then mounted upon resin and polymerized at 60 °C for 72 h. The ultramicrotome (Leica EM UC7, Leica, Wetzlar, Germany) was used for cutting the ultrathin sample sections (63 nm) placed on the copper grids and stained with 5% uranyl acetate and 0.2% lead citrate. The analysis of the chopped sections was carried out in a 200 KVA transmission electron microscope (Tecnai G2 20 twin, FEI, Hillsboro, OR, USA). The condition of the mitochondrial cristae was analyzed manually, and mitochondria with no clear double membrane and imperfect cristae were labeled as disrupted.

### 2.14. Chromatin Immunoprecipitation

The conditioned media were collected from cultured A549 cells transduced with ORF9b and NSP4 or empty vector lentivirus particles. These were concentrated using 10 kDa Amicon filters. For each sample, 20 mL of the media was collected and processed. The concentrated media were then used for chromatin immunoprecipitation. An amount of 5 µL anti-TFAM (CST) antibody was added to 500 µL media supernatant and incubated overnight at 4 °C on a rotatory shaker. The following day, 50 µL of Protein A/G Sepharose (Abcam, Boston, MA, USA) was added and incubated for 4 h at RT. The beads were then spun down and the supernatant was discarded. The bead pellets were then washed with a 50 µL lysis buffer twice. Half of the samples were processed for immunoblotting (described later) and the other half were used for PCR (as mentioned above).

### 2.15. Immunofluorescence

Cells seeded on coverslips with a confluency of 60–70% were used for immunofluorescence according to pre-established protocols [46]. In brief, the 4% PFA fixed cells were washed with 1X PBS for 10 min, then permeabilized in a blocking solution containing 1X PBS with 0.1% Triton X-100 and 5% goat serum for 1 h at RT. Cells were incubated with primary antibodies (2 µg/mL) overnight in 1X PBS containing 0.01% Triton X-100 and 2% goat serum at 4 °C. The next day, the cells were washed thrice with 1x PBS and incubated with fluorophore-tagged secondary antibodies (1 µg/mL) in a buffer (same as the primary antibody) for 1 h at RT. Cells were washed five times with 1X PBS for 5 min each at RT after secondary antibody incubation. Finally, the coverslip was mounted on a frosted slide with DAPI-containing mounting media.

### 2.16. Preparation of Mitochondrial and Cytosolic Fractions

Mitochondrial and cytosolic (mito-cyto) fractions were separated using a mitochondrial isolation kit (Sigma) according to the manufacturer’s instructions. Briefly, cells from two T75 flasks were harvested when they reached a confluence of 70–80% after a PBS wash, followed by trypsinization. Cells were pelleted at 1200 rpm for 5 min. Pellets were then resuspended in 400 μL of extraction buffer mixed with a protease inhibitor cocktail (Sigma) at a ratio of 1:100 and kept for 20 m on ice. To lyse the cells, each sample was passed 15–20 times through a 26-gauge syringe. Cells were analyzed for 50% lysis by live cell count using trypan blue. This was followed by centrifugation at 600× *g* for 10 min at 4 °C. The supernatant was then processed further by high-speed centrifugation at 11,000× *g* for 10 min at 4 °C. The obtained supernatant was the cytoplasmic fraction, whereas the pellet constituted the mitochondrial fraction. The extracted mitochondrial fractions were resuspended in 50 µL cell lysate mixed with a protease inhibitor cocktail at a 1:100 ratio and quantified. The cytosolic fractions were used for probing with an anti-TFAM antibody (1:1000, Abcam, Boston, MA, USA). For immunoblotting of TFAM in the cell supernatant, the sample was subjected to filtration through a 0.2 μm filter followed by concentration with 10 kDa Amicon filters. Protein estimation was conducted and 30 μg of the concentrated supernatant protein was used and resolved in 10% gel.

### 2.17. Immunoblotting

**Figure 1:**
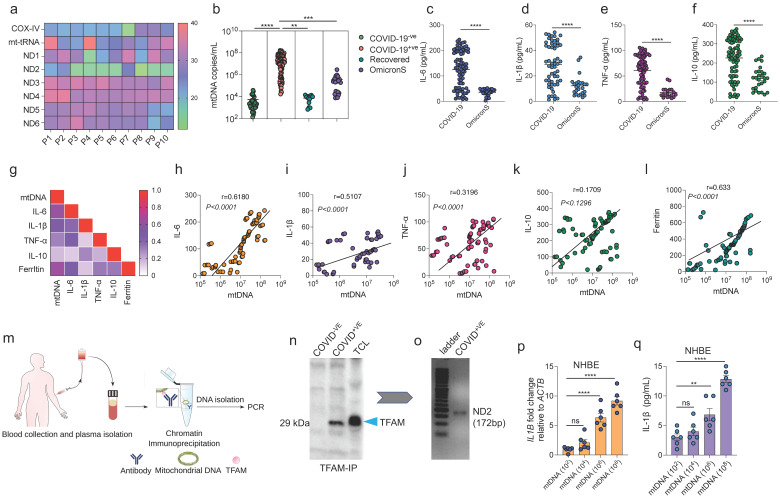
Mitochondrial DNA (mtDNA) release is positively correlated with the induction of a pro-inflammatory response in patients with coronavirus disease (COVID-19). (**a**) Heatmap showing the sensitivity of the detection of various mitochondrial genes for the reliable detection of mtDNA in the clinical plasma samples of patients with COVID-19. P1–P10 indicate plasma samples from 10 different patients. The range scale indicates the threshold cycle (Ct) values obtained from polymerase chain reaction (PCR) analysis. (**b**) Quantification of the mtDNA copy number in the plasma samples of severe acute respiratory syndrome coronavirus 2 (SARS-CoV-2)-negative (n = 35), SARS-CoV-2-positive (n = 88), recovered (n = 13), and suspected Omicron variant-positive (OmicronS) (n = 24) cases using PCR analysis. The results are expressed as copies/mL. (**c**–**f**) Quantitative analysis of IL-6, IL-1β, TNF-α, and IL-10 levels in the plasma of the indicated groups, as measured by ELISA. Data are represented in picograms/mL. (**g**) Spearman correlation matrix showing correlation of mtDNA levels with IL-6, IL-1β, TNF-α, IL-10, and ferritin. (**h**–**l**) Scatter plots showing correlation of mtDNA with IL-6, IL-1β, TNF-α, IL-10, respectively. A higher positive ‘r’ value between 0 and 1 indicates a higher positive correlation. (**m**) Schematic representation of the experimental approach used to isolate mtDNA from COVID-19 patient plasma. Patient blood was collected to isolate plasma, followed by chromatin immunoprecipitation (ChIP) with anti-TFAM antibody. DNA was isolated from the ChIP sample and ND2 PCR was performed to confirm isolation of mtDNA. (**n**) Representative immunoblot showing expression of TFAM in ChIP samples. The ChIP assay was performed in the plasma samples of COVID-19^-ve^, COVID-19^+ve^ using anti-TFAM antibody, followed by immunoblotting with anti-TFAM. Total cell lysate prepared from A549 cells (TCL) was used as a positive control for the detection of TFAM. (**o**) Representative PCR gel showing expression of ND2 gene (used as marker for mtDNA) in DNA extracted from ChIP samples. (**p**) Quantitative analysis of relative mRNA expression of *IL1B* with respect to *ACTB* in NHBE cells incubated with various doses of mtDNA obtained from COVID-19^+ve^ patients (as shown in *panel o*) (n = 6). (**q**) Quantitative analysis of IL-1β expression in the total protein extracted from NHBE cells, as determined by ELISA (n = 6). All data are represented as mean ± SEM of three independent experiments, analyzed by one-way ANOVA (**b**,**p**,**q**) and unpaired *t*-tests (**c**–**f**) using Graphpad Prism software. ** *p*  <  0.01; *** *p*  <  0.001; **** *p*  <  0.0001; ns: not significant.

For the immunoblotting experiments, cells were cultured in the appropriate media and transduced with the respective lentiviral-encoded plasmids or drugs. For total cell lysate (TCL) preparation, cells were washed once with PBS, followed by trypsinization and cell lysis using a RIPA buffer (Thermo Fisher Scientific, Boston, MA, USA) mixed with a protease inhibitor cocktail (Sigma) and DTT 0.1M (Sigma) at a ratio of 100:1:1. For cell lysis, the mixture was vortexed for 30 min (15 s each at 5 min intervals) at 4 °C. Isolated proteins were then quantified using the BCA method. An amount of 30 µg of protein was resolved in a 10% SDS-PAGE gel (unless mentioned otherwise elsewhere), then transferred on a PVDF membrane. After blocking of the PVDF membrane in 5% BSA in PBS, the respective primary antibodies were used (Figure 1n: anti-TFAM; Figure 3k: anti-GAPDH and anti-Cyt c; Figure 3l anti-GAPDH and anti-TFAM; Figure 3m: anti-E-cad and anti-TFAM; Figure 3o anti-TFAM; Figure 4d: anti-PINK1, anti-Parkin, and anti-VDAC, Figure 4L: anti-BAK, anti-BAX, and anti-VDAC; Figure 4n: anti-MCL1, anti-Bcl-xL, and anti-VDAC; Figure 4o: anti-BAK and anti-VDAC; Figure 4p: anti-BAX and anti-VDAC; Figure 4q: anti-MCL1 and anti-VDAC; Figure 5i: anti-GFP and anti-E-Cad; Figure 6a: anti-Strep II; Figure 6b: anti-BAK and anti-MCL1; Figure 6c: anti-BAK, anti-BAX, anti-Bcl-xL, anti-MCL1, and anti-GAPDH; Figure S4: anti-cGAS, anti-NLRP3, and anti-GAPDH) for overnight primary antibody incubation at 4 °C. All the primary antibodies were used in 1:1000 dilutions in PBST/TBST + 0.1% Tween20 along with 5% BSA. The full blots are shown in Figure S9.

After overnight incubation, the blots were washed thrice with PBST/TBST and probed with the corresponding secondary antibodies (1:10,000) conjugated with HRP and incubated for 1 h at RT. After washing the blots with PBST/TBST, they were incubated with chemiluminescence reagent (Invitrogen/Thermo Fisher Scientific, Boston, MA, USA) for 30 s–5 min according to band intensity and visualized using a ChemiDoc instrument (Bio-Rad Laboratories, Hercules, CA, USA). The image signals were quantified by performing image analysis using the Image J software.

Chromatin immunoprecipitated samples for TFAM immunoblotting were prepared as described above under the heading “*Chromatin immunoprecipitation*”. An amount of 30 µg of the extracted protein was used. Blots were incubated with anti-TFAM antibody overnight at 4 °C. Anti-rabbit secondary conjugated with HRP was used at a dilution of 1:10,000 and incubated for 1 h at RT. For anti-GFP immunoblotting, the cell supernatant was collected from scrambled or MCL1 shRNA-transduced cells after 48 h. Similarly, supernatants from NSP4 and ORF9b or VEC-transduced cells were collected after 48 h. The supernatant was subjected to filtration through 0.2 μm filters, then concentrated using 10 kDa Amicon filters, followed by protein estimation. An amount of 20 µg of protein was used and resolved in 10% gel. Anti-rabbit GFP and E-Cadherin antibodies were together used overnight at 4 °C. Anti-rabbit secondary conjugated with HRP was used at a dilution of 1:10,000 and incubated for 1 h at RT. Blots were developed using chemiluminescence substrate (Invitrogen/Thermo Fisher Scientific, Boston, MA, USA) at different exposure times on a ChemiDoc Imaging System (Bio-Rad Laboratories, Hercules, CA, USA).

### 2.18. Immunoprecipitation

Immunoprecipitation of Strep-II-tagged NSP4 and ORF9b was performed using 500 μg of total cell lysate with 5 μg of anti-Strep-II antibody (Abcam, Boston, MA, USA) or rabbit anti-IgG (Abcam, Boston, MA, USA) as a control. The cell lysate–antibody mix was incubated for 12 h at 4 °C. Immunoprecipitation was performed as mentioned in the commercially available immunoprecipitation kit (Abcam, Boston, MA, USA). Briefly, Protein A/G Sepharose (Abcam, Boston, MA, USA) blocked with BSA-skimmed milk (2:3 ratio) was added to the lysate and incubated for 1 h at room temperature. The antibody–protein lysate complex was eluted in 40 μL of 2X SDS-PAGE loading buffer and boiled for 5 min, followed by centrifugation to collect the eluent. SDS-PAGE was performed and the respective antibodies (anti-BAK, anti-BAK, anti-MCL1, and anti-Bcl-xL) were used. The VeriBlot Detection Reagent conjugated with HRP was used to probe the blots.

### 2.19. Co-Culture Studies and Intercellular Mitochondrial Transfer Assay

For the co-culture experiments, A549 or NHBE cells were transduced with NSP4 and ORF9b lentiviral-encoded particles for 24 h or with rotenone as previously described [41]. Simultaneously, MSCs were transduced with mitoGFP lentiviral particles. For the co-culture experiments, A549 cells were stained with Cell Tracker Deep Red (CTDR; Thermo Fisher Scientific, Boston, MA, USA) for 20 min and washed thrice with PBS. After this, MSC and A549 were co-cultured at a 1:1 ratio for another 24 h. Following co-culture, cells were visualized under live conditions. Images were obtained using a Nikon confocal Ti2E or Zeiss microscope integrated with an Apotome 2 (ZEISS Axio Observer 7). Mitochondrial donation from MSCs to A549 or NHBE was determined by specifically examining the mitoGFP signal in CTDR-positive cells and analyzed using Image J software. Similarly, to check the expression of BAK or MCL1 in A549 cells after co-culture, cells were stained with the respective antibodies. The signal from CTDR-positive A549 cells was quantitated, which represented the expression of BAK or MCL1 in these cells.

### 2.20. Pro-Inflammatory Cytokine, Cytochrome c in NHBE Cells

Patient plasma samples were used to measure the different cytokines by ELISA. The assay was performed in the hospital according to the manufacturer’s protocols. The cytokines profiled in the patient plasma samples included IL-6 (Krishgen Biosystems, Mumbai, MH, India), TNF-α (Krishgen Biosystems, Mumbai, MH, India), and IL-10 (Krishgen Biosystems, Mumbai, MH, India). The ferritin assay was performed according to the manufacturer’s protocols (Calbiotech, El Cajon, CA, USA). The human cytochrome c immunoassay was performed according to the manufacturer’s instructions (R&D Systems, Minneapolis, Min). Briefly, 20 µL of patient plasma sample was used for the assay and the input volume was adjusted to 100 µL using an assay diluent. The OD was measured at 450 nm using a microplate reader (Thermo Fisher Scientific, Boston, MA, USA).

To measure the pro-inflammatory response in NHBE cells, total levels of IL6 (BioLegend, San Diego, CA, USA) and IFN-γ (BioLegend, San Diego, CA, USA) were measured in total cell lysates. ELISA was performed according to the manufacturer’s protocols. Briefly, 10 µg of the total cell lysate was used for the measurement. The readings were obtained by the spectrophotometer (Multiskan SkyHigh Microplate Spectrophotometer, Thermo Fisher Scientific, Boston, MA, USA). Calculations were performed as previously described [35].

### 2.21. Image Acquisition and Analysis

**Figure 2:**
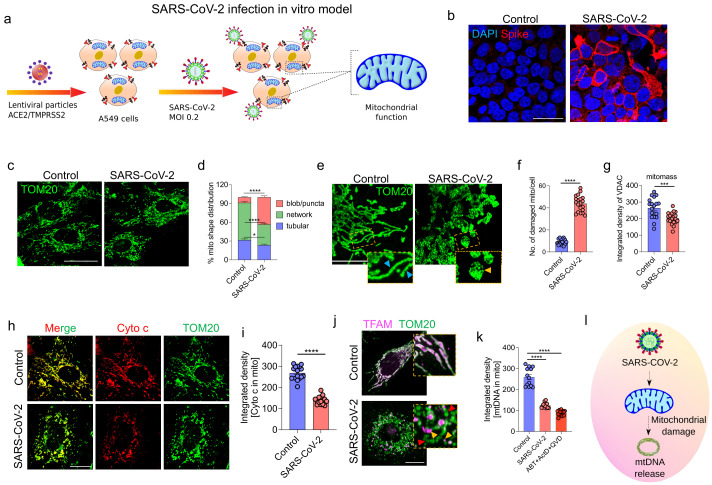
Severe acute respiratory syndrome coronavirus 2 (SARS-CoV-2) infection promotes mitochondrial damage and extrusion of mitochondrial DNA (mtDNA) from the mitochondria. (**a**) Schematic representation of the SARS-CoV-2 infection model. Analysis of mitochondrial morphology and function in SARS-CoV-2 (multiplicity of infection = 0.2)-infected A549 cells stably expressing ACE2 and TMPRSS2 (modified A549). (**b**) Representative immunofluorescence images of SARS-CoV-2-infected A549 cells probed with the anti-spike (red) antibodies. Nuclei were labeled with 4′,6-diamidino-2-phenylindole (blue). (**c**) Representative images of cells probed with the anti-TOM20 (green) antibodies to examine mitochondrial morphology. (**d**) Quantitative analysis of different mitochondrial shapes (n = 10 images). The following three mitochondrial morphologies were predominantly observed: blob/puncta, network, and tubular. Healthy cells exhibited increased proportions of tubular-shaped mitochondria, whereas SARS-CoV-2-infected cells exhibited increased proportions of blob/puncta-shaped mitochondria. Data are plotted as the percentage distribution of mitochondrial morphology. (**e**) Representative high-resolution LIGHTNING microscopy images of cells probed with the anti-TOM20 antibodies (green). Mitochondrial morphology was markedly different between the uninfected and infected groups. The insets show healthy tubular mitochondria (*blue arrowheads*) in the control cells and puncta/blob-shaped mitochondria with disrupted inner membranes (*yellow arrowheads*) (damaged mitochondria) in the SARS-CoV-2-infected cells. (**f**) Quantification of the number of damaged mitochondria (mito) per cell (n = 20) in the images shown in (**e**). (**g**) Quantitative analysis of mitochondrial mass (mitomass) in the control and infected groups using the anti-VDAC antibodies (n = 19). Data are plotted as integrated density. (**h**) Representative immunofluorescence images showing the localization of Cyt c (red) with the mitochondria (stained with the anti-TOM20 antibodies; green). (**i**) Quantification of the downregulated Cyt c signal (red) associated with the anti-TOM20 antibody-stained mitochondria (green) in SARS-CoV-2-infected cells from the images (n = 16). Data are plotted as integrated density. (**j**) Representative LIGHTNING microscopy images showing the colocalization of anti-TFAM antibody-stained mtDNA (magenta) within the anti-TOM20 antibody-stained mitochondria (green). Insets show that in the control group, mtDNA appears within the mitochondria. In the SARS-CoV-2-infected group, most mitochondria are devoid of mtDNA (*red arrowheads*) and in some instances, the mtDNA is localized outside the mitochondria (*yellow arrowheads*). (**k**) Quantitative analysis of the downregulation of mtDNA associated with the mitochondria in SARS-CoV-2-infected cells from images shown in (**j**) (n = 13). Data are plotted as integrated density. (**l**) Schematic representation of SARS-CoV-2 infection-induced mitochondrial damage and mtDNA release. All data are represented as mean ± standard error of mean from three independent experiments. Statistical analyses (unpaired *t*-tests) were performed using Graphpad Prism software. * *p*  <  0.05; *** *p*  <  0.001; **** *p*  <  0.0001; ns: not significant. Scale bars: 50 (**b**), 10 (**c**,**h**,**j**), or 5 μm (**e**).

**Figure 3:**
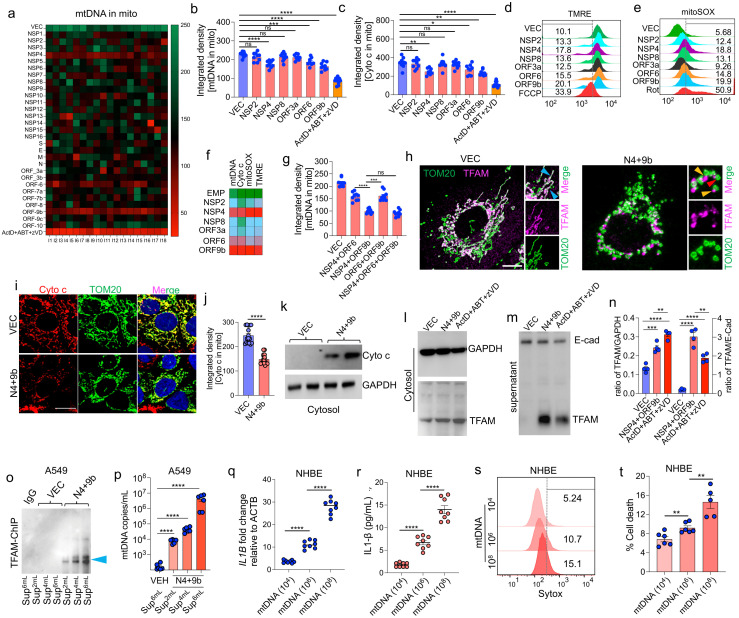
Severe acute respiratory syndrome coronavirus 2 (SARS-CoV-2) proteins NSP4 and ORF9b synergistically induce the extracellular release of mitochondrial DNA (mtDNA). (**a**) Heatmap showing the integrated density of mtDNA associated with the mitochondria in A549 cells transfected with 29 SARS-CoV-2 proteins. A549 cells were individually transfected with the constructs encoding the proteins and probed with the anti-TFAM antibodies. Images were acquired at 24 h post-transfection and subjected to densitometric analysis. The combination of actinomycin D, ABT-737, and zVD (ActD + ABT + zVD) (treatment for 2 h) was used as a positive control for the mtDNA release experiments. (**b**) Quantitative analysis of images of the indicated groups probed with the anti-TFAM (representing mtDNA inside mitochondria) antibodies (n = 9). A549 cells were transduced (48 h) with lentiviral-encoded shortlisted SARS-CoV-2 protein plasmids (as shown in *(***a**)) and probed with the anti-TFAM antibodies. Images were subjected to densitometric analysis. Corresponding representative images are shown in Figure S3d. Data are plotted as integrated density. (**c**) Quantification of Cyt c levels in the mitochondria. A549 cells transduced with the selected plasmids were subsequently probed with the anti-Cyt c antibodies and imaged after 48 h. Images were subjected to densitometric analysis (n = 9). Data are plotted as integrated density. (**d**,**e**) Representative flow cytometry histograms of cells stained with tetramethylrhodamine, ethyl ester (TMRE) (representing ΔΨm), and mitoSOX (representing mitochondrial reactive oxygen species (mtROS)) under the conditions mentioned in (**b**,**c**). (**f**) Heatmap of mtDNA and Cyt c release in A549 cells transduced with the selected protein-encoding constructs, which upregulated mtROS and downregulated ΔΨm. The values were calculated from the analysis data shown in (**b**–**e**). (**g**) Quantification of mtDNA release (n = 9) from cells co-transduced with various plasmids in the images. The combination of NSP4 and ORF9b robustly promoted the release of mtDNA. Data are plotted as integrated density. (**h**) Representative confocal images showing the localization of mtDNA (probed with anti-TFAM antibodies; magenta) with the mitochondria (probed with the anti-TOM20 antibodies; green) in cells transduced with the combination of NSP4 and ORF9b (N4 + 9b). Insets show the association of mtDNA with the mitochondria in vector (VEC)-transduced cells (*blue arrowheads*). In infected cells, mtDNA is mostly localized outside the mitochondria (*yellow arrowheads*) or the mitochondria are devoid of mtDNA (*red arrowheads*). (**i**) Representative immunofluorescence images showing Cyt c (red) localization with the mitochondria (probed with the anti-TOM20 antibodies; green) in cells transduced with VEC or N4 + 9b. (**j**) Quantitative analysis of images shown in (**i**) (n = 10). Data are plotted as integrated density. (**k**) Immunoblot of Cyto c in the cytosolic extract prepared from cells after transduction with N4+9b or VEC. GAPDH was used as a loading control. (**l**) Representative immunoblot showing the expression of TFAM in the cytosol. GAPDH served as the reference control. The cytosolic extract was prepared from cells transduced with VEC or N4 + 9b (48 h). The combination of ActD + ABT + zVD (treatment for 2 h) was used as the positive control for the experiments evaluating mtDNA release. (**m**) Representative immunoblots of concentrated cell supernatant from the cells treated as indicated in (**k**). E-Cadherin (E-Cad) was used as the reference control. (**n**) Densitometry analysis of proteins in the immunoblots shown in (**k**,**l**) (n = 4). Data are plotted as the ratio of TFAM and the reference control. (**o**) Representative immunoblot showing TFAM (pulled down using the anti-TFAM antibodies; mtDNA marker) expression in the immunoprecipitated samples. Chromatin immunoprecipitation (ChIP) was performed using the concentrated cell supernatant (Sup) with three different volumes as the starting materials (2, 4, and 6 mL). IgG was used as the isotype control and tested only at a higher concentration (6 mL). (**p**) Quantification of mtDNA copy number (calculated using polymerase chain reaction and represented as copies/mL). mtDNA was extracted from various supernatant volumes and subjected to ChIP with the anti-TFAM antibodies (n = 6). (**q**) Quantitative analysis of *IL1B* expression in normal human bronchial epithelial (NHBE) cells incubated with various mtDNA concentrations (24 h). Total RNA was extracted from the cells and subjected to quantitative real-time PCR (n = 8). (**r**) Quantitative analysis of IL-1β levels in the total protein samples extracted from NHBE cells, which were treated as described in (**p**). (**s**,**t**) Representative flow cytometry histogram plots showing NHBE cell death (determined using Sytox dye) induced by different mtDNA concentrations (n = 6). Data are plotted as percentage of cell death. All data are represented as mean ± standard error of mean from three independent experiments. Statistical analyses (one-way analysis of variance (**b**,**c**,**h**,**n**,**p**) and unpaired *t*-tests (**j**,**q**,**r**,**t**)) were performed using Graphpad Prism software. * *p* < 0.05, ** *p*  <  0.01; *** *p*  <  0.001; **** *p*  <  0.0001; ns: not significant. Scale bar: 10 μm.

**Figure 4:**
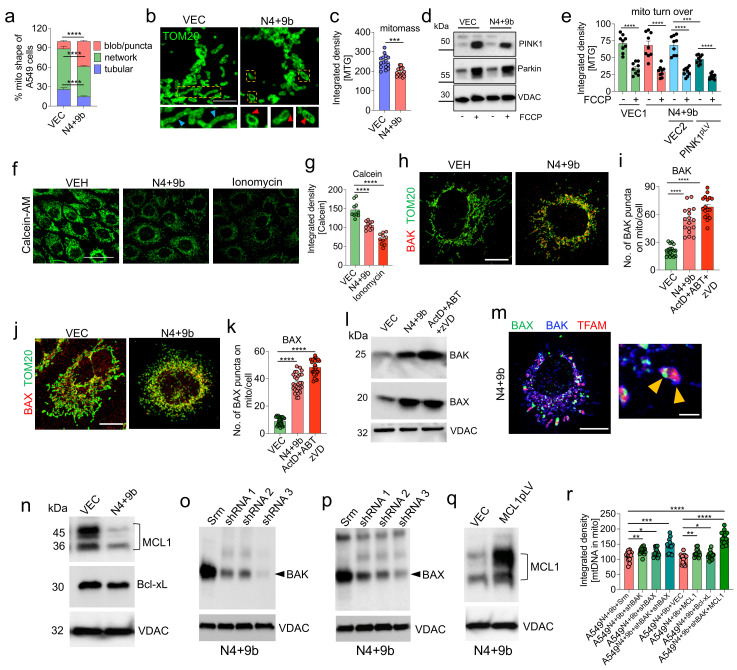
NSP4 and ORF9b induce mitochondrial macropore formation and consequently promote mitochondrial DNA (mtDNA) release. (**a**) Quantitative analysis of mitochondrial morphology changes in cells transduced with vector (VEC) or NSP4 + ORF9b (N4 + 9B) constructs and stained with MitoTracker Red (MTR) (n = 10 images). Representative images are shown in Figure S5a. (**b**) Representative LIGHTNING microscopy images showing healthy tubular mitochondrial morphology in VEC-transduced (*insets with blue arrowheads*) cells and puncta-shaped mitochondria (indicating mitochondrial damage) in N4 + 9b (*insets with red arrowheads*) construct-transduced cells. (**c**) Quantitative analysis of confocal images of cells transduced with VEC or N4 + 9b constructs and stained with MitoTracker Green (MTG) (representing mitochondrial mass (mitomass)) (n = 15). Data are plotted as integrated density. (**d**) Representative immunoblots showing the expression of PINK1 and Parkin in cells transduced with VEC or N4 + 9b and treated with carbonyl cyanide 4-(trifluoromethoxy)phenylhydrazone (FCCP) for 2 h. VDAC was used as the reference control. (**e**) Quantitative analysis of mitochondrial turnover indicated by the loss of the MTG signal under the conditions mentioned in (**d**) (n = 9). Data are plotted as integrated density. VEC1 refers to the backbone vector of NSP4 and ORF9b, whereas VEC2 refers to the vector in which MCL1 was cloned (**f**) Representative confocal images showing calcein-AM-stained cells to indicate mitochondrial permeability transition pore opening. Ionomycin (treatment for 2 h) was used as a positive control. (**g**) Quantitative analysis of images (n = 10) for the experiment described in (**f**). Data are plotted as integrated density. (**h**) Representative confocal images of cells treated as indicated and subjected to BAK (red) and TOM20 (green) immunostaining. (**i**) Analysis of images (n = 17) in (**h**) to quantify the BAK puncta associated with the mitochondria. The combination of actinomycin D, ABT-737, and zVD (ActD + ABT + zVD; reported to upregulate BAX and BAK levels on the mitochondria) was used as a positive control. (**j**) Representative confocal images of cells treated as indicated and subjected to BAX (red) and TOM20 (green) immunostaining. (**k**) Analysis of images (n = 27) in (**j**) to quantify the BAK puncta associated with the mitochondria. The combination of ActD + ABT + zVD was used as a positive control. (**l**) Representative immunoblots showing the expression of BAK and BAX in the mitochondrial extracts of cells transduced with VEC or N4 + 9b constructs. VDAC was used as the reference control. The combination of ActD + ABT + zVD was used as the positive control. (**m**) Representative confocal images of N4 + 9b construct-transduced cells showing mtDNA (TFAM; red) associated with the BAK (blue) and BAX (green) puncta, representing macropore formation. In the insets, yellow arrowheads show the colocalization of mtDNA with the BAK/BAX macropore. (**n**) Representative immunoblots showing MCL1 and Bcl-xL expression in cells transduced with VEC or N4 + 9b constructs. VDAC was used as the reference control. (**o**,**p**) Representative immunoblots showing the expression of BAK (**o**) and BAX (**p**) in cells co-transduced with the N4 + 9b construct and shRNA as indicated. VDAC was used as the reference control. (**q**) Representative immunoblots showing MCL1 expression in cells co-transduced with the N4 + 9b construct and vector (VEC) or MCL1. VDAC was used as the reference control. (**r**) Quantitative analysis of images (n = 15) of cells treated as indicated and immunostained with the anti-TFAM (representing mtDNA) antibodies. Data show signals associated with the anti-TOM20 antibody-stained mitochondria. Data are plotted as integrated density. All data are represented as mean ± standard error of mean from three independent experiments. Statistical analyses (unpaired *t*-tests (**a**,**c**,**g**,**l**,**k**) and one-way analysis of variance (**e**,**r**)) were performed using Graphpad Prism software. * *p* < 0.05, ** *p*  <  0.01; *** *p*  <  0.001; **** *p*  <  0.0001; ns: not significant. Scale bars: 5 (**b**), 50 (**f**), or 10 μm (**h**,**i**,**m**).

**Figure 5:**
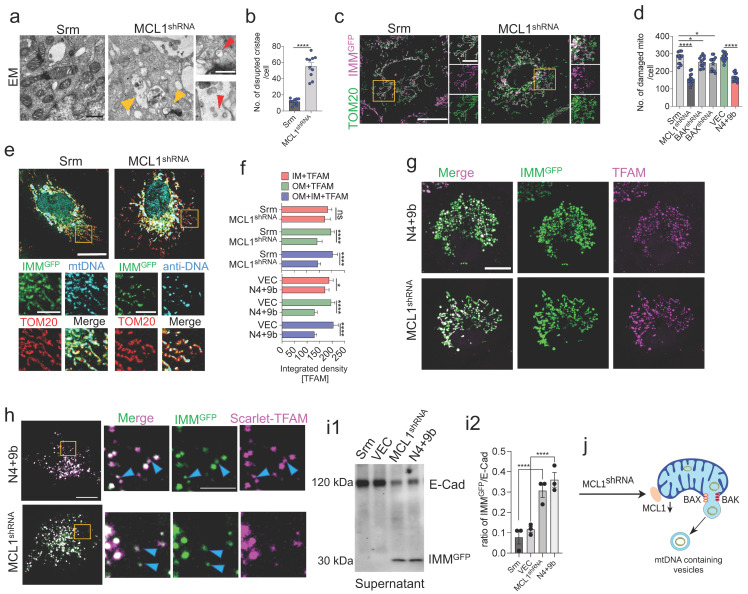
MCL1 regulates inner membrane integrity and mitochondrial DNA (mtDNA) release via inner mitochondrial membrane (IMM)-derived vesicles. (**a**) Representative electron microscopy (EM) images showing the disruption of cristae (yellow arrowheads) and the formation of vesicle-like structures (insets with red arrowheads) in cells transfected with sh-MCL1. (**b**) Quantitative analysis of results shown in (**a**) representing the disrupted inner membrane cristae (n = 10). (**c**) Representative LIGHTNING microscopy images showing the loss of inner membrane integrity. Cells were co-transduced with the non-targeted scramble control (Srm) or sh-MCL1 and IMM-targeted green fluorescent protein (GFP) (IMM^GFP^) and probed with the anti-TOM20 antibodies. (**d**) Quantitative analysis of results shown in (**c**) representing damaged mitochondria (n = 13). (**e**) Representative images of cells triple-stained with IMM^GFP^ (green), anti-DNA antibodies (representing mtDNA; cyan), and anti-TOM20 antibodies (red). Cells were transduced with Srm or sh-MCL1. (**f**) Quantification of images of cells co-transduced with VEC or NSP4 + ORF9b and Srm or sh-MCL1 and immunostained with anti-TFAM antibodies. Data are plotted as integrated densities of TFAM associated with IMM, outer mitochondrial membrane (OMM), or both (n = 9). (**g**) Representative images of TFAM staining (representing mtDNA) in cells co-transduced with NSP4 + ORF9B or MCL1 shRNA and IMM^GFP^. (**h**) Representative live-cell images showing the appearance of vesicles containing mtDNA (magenta) and IMM (green) in cells co-transfected with IMM^GFP^ and Scarlet-TFAM. Small vesicles containing both mtDNA and IMM are seen (blue arrowheads) near the large vesicles (usually mitochondria). (**i1**) Representative immunoblot showing the presence of IMM^GFP^ in cell supernatants collected 24 h after transfection with IMM^GFP^, concentrated, and probed with the anti-GFP antibodies. E-Cad was used as the reference control. (**i2**) Densitometry analysis of the blots shown in (**i1**). (**j**) Summary of the results showing mtDNA derived from the inner membrane vesicles extruding from the BAK/BAX-mediated macropores. These IMM pores are regulated by MCL1. All data are represented as mean ± standard error of mean from three independent experiments. Statistical analyses (unpaired *t*-tests (**b**,**f**) and one-way analysis of variance (**d**)) were performed using Graphpad Prism software. * *p* < 0.05; **** *p*  <  0.0001; ns: not significant (non-parametric *t*-test). Scale bars: 0.5 (**a**), 0.2 (ROI), or 10 μm (**c**,**e**,**g**,**h**).

**Figure 6:**
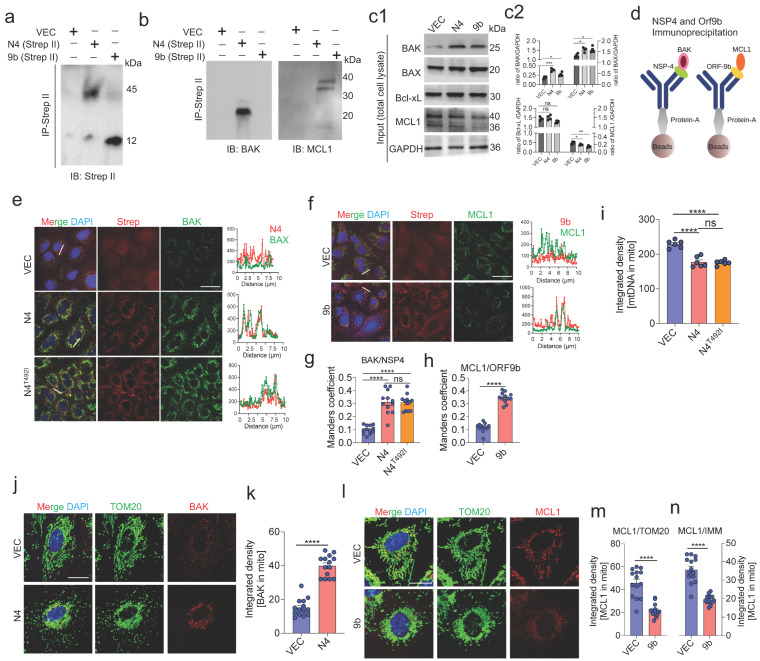
NSP4 directly binds and stabilizes BAK, whereas ORF9b binds and decreases the MCL1 levels on the mitochondrial surface. (**a**) Representative immunoblot showing the expression of NSP4 and ORF9b (using anti-Strep-II antibodies) in A549 cells transduced with Strep-II-tagged NSP4 or ORF9b (24 h) and subjected to immunoprecipitation and immunoblotting. (**b**) The immunoprecipitated samples in (**a**) were further probed with anti-BAK and anti-MCL1 antibodies. Representative immunoblot showing the expression of BAK and MCL1. (**c**) Representative immunoblot showing the expression of BAK, BAX, BCL-xL, and MCL1 in the total input protein, which was used as a control in the immunoprecipitation experiment shown in (**a**,**b**). GAPDH was used as the reference control. (**d**) Schematic illustration showing the immunoprecipitation workflow. (**e**) Representative images showing the expression of Strep-II (**red**) and BAK (green) in cells transduced with VEC, wild-type NSP4 (N4), or mutant NSP4 (detected in the Omicron variant (N4^T92I^)). Nuclei were labeled with 4′,6-diamidino-2-phenylindole (blue). *Right panels* show the line scans of the area as shown in (**e**). (**f**) Representative images showing the expression of MCL1 (green) and Strep-II (red) in cells transduced with ORF9b and subjected to immunostaining. Corresponding line scans are shown in *the right panel.* (**g**,**h**) Data are plotted as the Mander’s overlap coefficient between BAK and NSP4, as well as between MCL1 and ORF9b (n = 12). (**i**) Data are plotted as integrated densities of mitochondrial DNA (mtDNA) in cells transduced with VEC, N4, or N4^T92I^ (n = 10). (**j**) Representative images show the BAK signal (red) associated with anti-TOM20 antibody-stained mitochondria (green) in cells transduced with VEC or NSP4. (**k**) Quantitative analysis of data from (**j**). Data are plotted as integrated densities (n = 14). (**i**) Representative images showing the expression of MCL1 (red) and TOM20 (green) in cells transduced with VEC or ORF9b. (**m**) Quantitative analysis of data from (**l**). Data are plotted as integrated densities (n = 14). (**n**) Quantitative analysis of images in which cells were treated as indicated in (**l**,**m**). However, instead of staining the cells with the anti-TOM20 antibody, the cells were stained with the anti-COX-IV antibody to locate the MCL1 signal associated with the mitochondrial inner membrane. All data are represented as mean ± standard error of mean from three independent experiments. Statistical analyses (one-way analysis of variance (**g**,**h**,**i**) and unpaired *t*-tests (**k**,**m**,**n**)) were performed using Graphpad Prism software. * *p* < 0.05, ** *p*  <  0.01; *** *p*  <  0.001; **** *p*  <  0.0001; ns: not significant (non-parametric *t*-test). Scale bars: 20 (**e**,**f**) or 10 μm (**j**,**l**).

**Figure 7:**
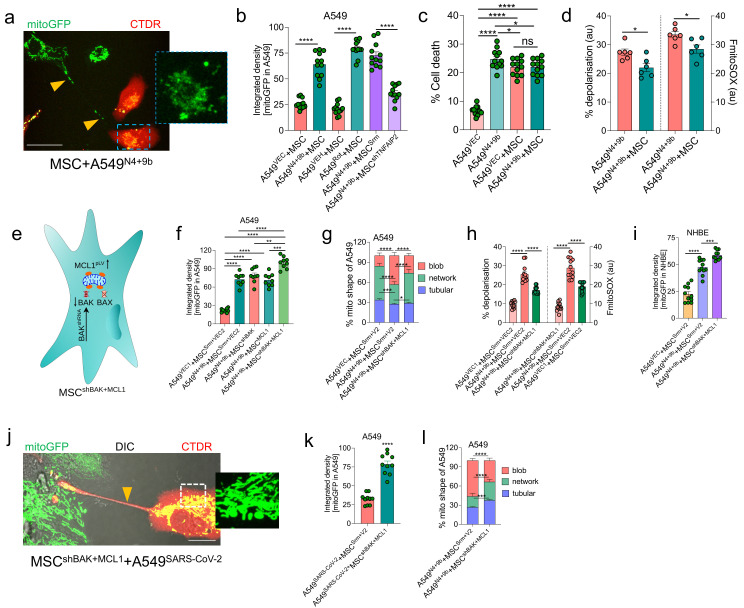
Mesenchymal stem cells (MSCs) engineered to exhibit BAK downregulation and MCL1 overexpression promoted functional mitochondrial transfer to stressed airway epithelial cells. (**a**) Representative image showing CellTracker Red (CTDR)-stained A549 cells (red) and MSCs expressing mito-GFP (green) in a co-culture setup. The mito-GFP signal is observed in A549 cells after 24 h of co-culture (yellow arrowheads), which suggests intracellular mitochondrial transfer (IMT) from MSCs. The cells were transduced with NSP4 + ORF9b (A549^N4+9b^). (**b**) Quantitative analysis of data from the experiment in (**a**). Data are plotted as integrated densities (n = 12). A549 cells were transduced as shown in (**a**) or with VEC, whereas MSCs were transduced with the non-targeted scramble control (Srm) or shRNA targeting *TNFAPI2*. Rotenone (Rot)-treated A549 cells served as positive controls. Rotenone induces mitochondrial stress for IMT. Dimethyl sulfoxide (DMSO) was used as the vehicle control (VEH). (**c**) Quantitative analysis of percentage (%) cell death in A549^N4+9b^ cells 72 h after co-culture. Cell death was analyzed using flow cytometry with specific gating on Sytox-positive and CTDR-stained A549 cells (n = 12). (**d**) Quantitative analysis of ΔΨm (represented as % depolarization) and mitochondrial reactive oxygen species (mtROS) (as FmitoSOX) in CTDR-gated A549 cells in a co-culture setup of CTDR-stained A549 cells and mito-GFP-expressing MSCs. ΔΨm and mtROS were measured by staining the co-culture with tetramethylrhodamine, ethyl ester (TMRE), and mitoSOX, respectively (n = 6). (**e**) Schematic illustration showing the experimental design in which the MSCs were double-engineered to knock down BAK using shRNA and overexpress MCL1 (MCL1^pLV^) using lentivirus-encoded plasmids (MSC^shBAK+MCL1^). (**f**) Quantitative analysis of mito-GFP in CTDR-gated A549 in a co-culture setup. As shown in (**b**,**e**), engineered MSCs were co-cultured with A549 cells in different combinations as indicated. Data are plotted as integrated densities (n = 9). (**g**) Quantitative analysis of mitochondrial morphological changes by specifically quantifying the signal in the A549 cells (n = 10). The co-culture conditions are similar to those shown in (**f**). (**h**) Quantitative analysis of ΔΨm (represented as % depolarization) and mtROS (as FmitoSOX) in CTDR-gated A549 cells in a co-culture setup of CTDR-stained A549 cells and mito-GFP-expressing MSCs. ΔΨm and mtROS were measured by staining the co-culture with TMRE and mitoSOX, respectively (n = 12). (**i**) Quantitative analysis of mito-GFP in normal human bronchial epithelial (NHBE) cells co-cultured with MSC^shBAK+MCL1^. Data are plotted as integrated densities (n = 11). (**j**) Representative image showing CTDR-stained and severe acute respiratory syndrome coronavirus 2 (SARS-CoV-2)-infected A549 cells co-cultured with mito-GFP-expressing MSC^shBAK+MCL1^. Mito-GFP signal is observed in SARS-CoV-2-infected A549 cells after 24 h of co-culture, indicating intracellular mitochondrial transfer (IMT) from MSCs. Yellow arrowhead shows tunneling nanotubes (TNTs) containing mitochondria. Inset shows that MSCs donated mitochondria (green) to A549 cells. (**k**) Quantitative analysis of mitochondrial transfer from MSC^shBAK+MCL1^ to SARS-CoV-2-infected A549 cells by calculating the mito-GFP signal in A549 cells. Data are plotted as integrated densities (n = 10). (**l**) Quantitative analysis of mitochondrial morphological changes in A549 cells transduced with VEC or NSP4 + ORF9b and co-cultured with MSCs transduced with Srm or shBAK + MCL1. All data are represented as mean ± standard error of mean from three independent experiments. Statistical analyses were performed (unpaired *t*-tests (**b**,**d**,**g**,**i**,**k**,**l**) and one-way analysis of variance (**c**,**f**,**h**)) using Graphpad Prism software. * *p* < 0.05, ** *p*  <  0.01; *** *p*  <  0.001; **** *p*  <  0.0001; ns: not significant (non-parametric *t*-test). Scale bars: 50 μm.

**Figure 8:**
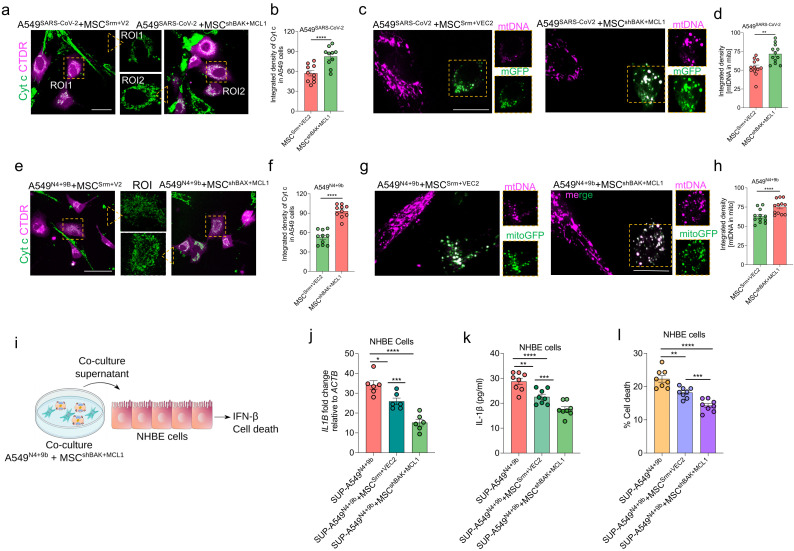
BAK knockdown and MCL-overexpressing mesenchymal stem cells (MSC^shBAK+MCL1^) prevent pro-inflammatory mitochondrial DNA (mtDNA) release from severe acute respiratory syndrome coronavirus 2 (SARS-CoV-2)-infected or NSP4 + ORF9b-transduced airway epithelial cells. (**a**) Representative confocal images showing the CellTracker Red (CTDR)-stained A549 cells co-cultured with unlabeled MSC^shBAK+MCL1^. Before co-culture, A549 cells were infected with SARS-CoV-2 for 24 h and stained with CTDR (magenta). After 24 h of co-culture, cells were probed with the anti-Cyt c antibodies (green). ROI indicates the representative region of interest used for image analysis. (**b**) Quantitative analysis of Cyt c signal (green) in CTDR-stained A549 cells in (**a**). Data are plotted as integrated densities (n = 10). (**c**) Representative confocal images showing anti-TFAM (representing mtDNA, magenta) antibody signals in SARS-CoV-2-infected A549 co-cultured with the indicated construct-transduced MSCs. (**d**). Quantitative analysis of mtDNA signal associated with mitochondria in (**c**) (n = 12). (**e**) Representative confocal images showing CTDR-stained A549 cells co-cultured with unlabeled MSC^shBAK+MCL1^. Before co-culture, A549 cells were transduced with the NSP4 + ORF9b construct or vector (VEC) for 24 h and stained with CTDR (magenta). After 24 h of co-culture, cells were probed with anti-Cyt c (green) antibodies. (**f**) Quantitative analysis of Cyt c signal (green) in CTDR-stained A549 cells in (**e**). Data are plotted as integrated densities (n = 10). Mito-GFP signal obtained from CTDR-stained A549 cells. (**g**) Representative confocal images of NSP4 + ORF9b-transduced A549 cells co-cultured with the indicated construct-transduced MSCs and probed with the anti-TFAM (representing mtDNA, magenta) antibodies. (**h**). Quantitative analysis of mtDNA signal associated with mitochondria in (**g**) (n = 12). (**i**) Schematic representation of the experimental procedure for co-culturing A549^N4+9b^ cells with MSC^shBAK+MCL1^ and transferring the supernatant to normal human bronchial epithelial (NHBE) cells. The pro-inflammatory response and cell death were analyzed in NHBE cells treated with the supernatant for 24 h. (**j**) Quantitative analysis of *IL1B* expression in NHBE cells incubated with co-culture supernatant as shown in (**i**). (**k**) Quantitative analysis of IL-1β expression in NHBE cells incubated with co-culture supernatant (treatment was performed as shown in (**i**)) using an enzyme-linked immunosorbent assay. (**l**) Cell death was quantified by staining NHBE cells with Sytox and subjecting them to flow cytometry. Cell death in NHBE cells incubated with supernatant for 48 h (treatment was performed as shown in (**i**)) and plotted as a percentage of cell death. All data are represented as mean ± standard error of mean from three independent experiments. Statistical analyses (unpaired *t*-tests (**b**,**d**,**f**,**h**) and one-way analysis of variance (**j**,**k**,**l**) were performed using Graphpad Prism software. * *p*  <  0.05, ** *p*  <  0.01, *** *p*  <  0.001, and **** *p*  <  0.0001. Scale bar: 50 μm.

Four different fluorescence microscopy instruments were used for the image acquisition. The images shown in Figures 2c,h; 4f; 6e,f,j,l; 7a,j; 8a,c,e,j; and Figure S7d were acquired using a Nikon confocal Ti2E microscope. The images were acquired with a 60× objective with 1.49 NA. The images shown in Figures 2e,j; 3h; 4b,m; Figures S2a,b and S6b were acquired using the LIGHTNING module of the TCS SP8 LSCM. The images shown in Figures 2b,3i and Figure S2c were acquired using a Zeiss microscope integrated with an Apotome 2 (ZEISS Axio Observer 7). The images were acquired with 63 × oil objectives with 1.4 NA. For the live-cell imaging, as shown in Figure 5h, the images were acquired in cell culture media without phenol red. Similarly, for the live-cell co-culture imaging (Figure 7 and Figure S7), the images were acquired in media without phenol red. The images shown in Figure S8a were acquired with the EVOS FLoid Imaging System (Thermo Fisher Scientific, Boston, MA, USA) using a 20 × objective. All other images shown were acquired with a TCS SP8 LSCM (Leica) using a 63 × oil objective with 1.4 NA.

Post-image acquisition and image processing were performed using Image J software. Mitochondrial shape classification was performed following the previously described protocols [47]. For the integrated density calculation, the image was first processed to mask the signal outside by specifically focusing on the mitochondrial stain, which was stained with either TOM20 or MitoTracker Red. The signal outside the mitochondria was eliminated after processing in all channels. Next, images were split into three different channels as previously mentioned [46]. To specifically measure the signal associated with the mitochondria, only channel images with corresponding signals of the TFAM signal or PicoGreen or anti-DNA stained (representing mtDNA) images were used. The channel with the mtDNA signal and mitochondrial signals was selected and the threshold was set independently using the OTSU threshold method. Finally, the mask of the segmented image of the mitochondria was generated and applied to the TFAM segmented image, thus clearing the signal outside of the mitochondria. Finally, the image was redirected to measure the integrated density parameters. Similarly, for the calculation of the BAX and BAK puncta (Figure 4i,k), the signal outside the mitochondria was masked and the number of puncta associated with the mitochondria was calculated. The number of puncta was calculated per image and represented as puncta per cell.

For colocalization analysis, the ‘EzColocalization’ plugin [48] was used, as previously described [46]. The Mander’s overlap coefficient (or Mander’s coefficient) was calculated between BAK and NSP4, and MCL1 and ORF9b, as shown in Figure 6g,h. The Mander’s coefficient measures the percentage colocalization as described by us previously [46].

### 2.22. Statistical Analyses

*P*-values for single comparisons were calculated using an unpaired *t*-test, whereas one-way ANOVA with Tukey’s post hoc test was used for independent multiple comparisons using GraphPad Prism software (Prism v.9). All the experiments were repeated a minimum of three times, and the figures shown in this manuscript were prepared using GraphPad Prism.

## 3. Results

### 3.1. Positive Correlation between mtDNA Release and Pro-Inflammatory Immune Response in Patients with COVID-19

This study aimed to establish the correlation between mtDNA release and the SARS-CoV-2-induced pro-inflammatory responses in patients with COVID-19. A set of mitochondrial genes was examined to identify a marker that can consistently and reliably detect mtDNA in the cell-free (cf) plasma samples of patients. The initial screening of 10 randomly selected patient plasma samples revealed that *ND2* provided the most consistent results compared with the other tested mitochondrial genes (Figure 1a). Using *ND2* as an mtDNA marker, the plasma samples of 89 SARS-CoV-2-positive cases, 35 SARS-CoV-2-negative cases, 14 convalescent cases (recovered; previously infected cases confirmed to be SARS-CoV-2-negative using quantitative real-time polymerase chain reaction (RT-qPCR)), and 24 new cases suspected to be infected with the Omicron variant were profiled (Figure S1a). Compared with those in the SARS-CoV-2-negative and COVID-19-recovered (hereafter referred to as recovered) groups, the mtDNA copy number was significantly higher in the SARS-CoV-2-positive group (Figure 1b). The mtDNA levels in patients suspected to be infected with the Omicron variant (samples collected between January 2022 and March 2022; hereafter referred to as OmicronS) were lower than those in patients infected with other variants (samples collected between June 2020 and October 2020; hereafter referred to as COVID-19), which are reported to yield S gene-positive results [41].

To examine the correlation between the cf-mtDNA levels and pro-inflammatory response, the levels of cytokine storm-associated key inflammatory markers were analyzed using an enzyme-linked immunosorbent assay. The IL-6, IL-1β, TNF-α, and IL-10 levels were consistently upregulated in patients with COVID-19 (Figure 1c–f). Similar to the mtDNA levels, the levels of PICs (IL-6, IL-1β, and TNF-α) and IL-10 were consistently downregulated in patients with OmicronS infection. Correlation analysis revealed that the IL-1β, IL-6, TNF-α, and ferritin levels were positively correlated with the mtDNA levels (Figure 1g–l). These results suggest that the plasma mtDNA levels are closely correlated with the PIC response in patients with COVID-19.

To examine if cf-mtDNA directly elicits the inflammatory response, an in vitro model of normal human bronchial epithelial (NHBE) cells was used. The cells were incubated directly with the plasma samples of the SARS-CoV-2-positive (serologically positive) and SARS-CoV-2-negative (serologically negative) cases [41]. Cells were incubated with plasma for 24 h(h) and total RNA was extracted and subjected to RT-qPCR analysis. The *IL1B* mRNA levels were significantly upregulated in the cells incubated with the plasma of the SARS-CoV-2-positive cases (Figure S1b). Compared with that of the SARS-CoV-2-negative cases, the plasma of the SARS-CoV-2-positive cases induced cell death at 48 h post-incubation (Figure S1c,d).

The plasma of patients with COVID-19 comprises various pro-inflammatory factors and DAMPs hence, the ability of mtDNA to induce the pro-inflammatory response was examined. Chromatin immunoprecipitation (ChIP) of TFAM (a mitochondrial DNA nucleoid-associated protein) was performed using 10 pooled patient samples divided into eight groups each (Figure 1m). The immunoprecipitated mtDNA was subjected to DNA extraction. The presence of *ND2* in the extracted DNA was verified using PCR (Figure 1n and Figure S1e). Total protein and mRNA were extracted from NHBE cells incubated with various copy numbers of the extracted mtDNA. Consistent with the results obtained using the SARS-CoV-2-positive plasma samples (Figure S1B), purified mtDNA from these samples dose-dependently upregulated the *IL1B and IL6* mRNA levels in NHBE cells (Figure 1p and Figure S1f). Additionally, purified mtDNA upregulated the IL-1β and IL-6 levels (Figure 1q and Figure S1g). Consistent with the data shown in Figure S1c,d, mtDNA isolated from the SARS-CoV-2-positive plasma samples increased NHBE cell death (Figure S1h,i). These findings indicate that mtDNA isolated from the plasma of patients with COVID-19 induces pro-inflammatory responses and cell death.

Next, this study aimed to determine if mtDNA release is a general phenomenon associated with SARS-CoV-2 infection-induced mitochondrial damage. As mitochondrial damage is often associated with the release of pro-apoptotic Cytochrome c (Cyt c) [49], the plasma levels of Cyt c were measured in patients with COVID-19. Consistent with the findings of mtDNA release, the plasma Cyt c levels in the SARS-CoV-2-positive cases were upregulated compared with those in the SARS-CoV-2-negative cases (Figure S1j). These findings indicated that cf-mtDNA is a robust indicator of mitochondrial damage in patients with COVID-19 and that it can be used as a potential biomarker along with other pro-inflammatory markers to determine COVID-19 severity.

### 3.2. SARS-CoV-2 Infection Induces Epithelial Cell Mitochondrial Dysfunction and mtDNA Release

To evaluate the effect of SARS-CoV-2 on the morphology and function of the mitochondria in airway epithelial cells, SARS-CoV-2 infection-permissible A549 cells were generated by stably expressing ACE2/TMPRSS2 and infected with SARS-CoV-2 (strain USA-WA1/2020) for 48h (Figure 2a). Next, the cells were fixed and immunostained with an anti-spike protein antibody to confirm SARS-CoV-2 infection (Figure 2b). The mitochondrial structural changes in the infected cells were examined using immunofluorescence analysis with the anti-TOM20 (OMM protein) antibody. SARS-CoV-2 infection induced robust mitochondrial structural changes. The infected cells exhibited punctate/blob mitochondrial morphology, whereas the control cells exhibited an elongated tubular network of mitochondria (Figure 2c). These changes were also confirmed using image analysis (Figure 2d). High-resolution LIGHTNING microscopy imaging analysis revealed swollen cristae and disrupted inner mitochondrial membranes (IMM; represented as damaged mitochondria; 8.85 ± 1.63 (control group) vs. 43.9 ± 1.63 (SARS-CoV-2-infected group)) (Figure 2e,f, and Figure S2a) and decreased mitochondrial mass in the SARS-CoV-2-positive samples (Figure 2g). To investigate the functional impact of these mitochondrial structural changes, the mitochondrial pro-apoptotic Cyt c levels were determined using immunostaining. The Cyt c signal associated with the mitochondria was markedly downregulated (269 ± 8 (control group) vs. 140.1 ± 4.7 (SARS-CoV-2-infected group)) in the SARS-CoV-2 infected cells, indicating a robust release of Cyt c into the cytosol (Figure 2h,i). Next, the effect of SARS-CoV-2 infection on pro-inflammatory mtDNA release was examined using LIGHTNING microscopy with the anti-TFAM (to detect mtDNA) and anti-TOM20 (to visualize OMM) antibodies. In the control group, TFAM was associated with the mitochondria, especially within the mitochondria. However, the mitochondria were largely devoid of mtDNA with mtDNA visualized just outside the mitochondria in the SARS-CoV-2-infected cells, indicating the release of mtDNA (Figure 2j and Figure S2b). As a positive control for the mtDNA release experiments, cells were treated for 2h with a combination of actinomycin D, ABT-737, and zVD, which are known to induce mtDNA release. The TFAM signal intensity was significantly downregulated in the SARS-CoV-2-infected cells (259.3 ± 10.8, 124.7 ± 3.15, and 88.7 ± 3.2 in the control, SARS-CoV-2-infected, and positive control groups, respectively) (Figure 2k). We hypothesized that the extrusion of mtDNA into the cytoplasm may have activated the cytosolic DNA sensors, such as the cGAS-STING1 and/or NLRP3 inflammasome. However, the expression levels of cGAS or NLRP3 were not significantly different between the control and SARS-CoV-2-infected groups (Figure S2c–e). These findings indicate that SARS-CoV-2 infection induces robust mitochondrial structural changes and mtDNA release in lung epithelial cells (Figure 2l), which were consistent with the mtDNA release observed in patients with COVID-19. In contrast to the findings of previous studies on endothelial cells, mtDNA extruded from airway epithelial cells did not induce the expression of canonical DNA sensors in the cytosol in this study [50].

### 3.3. SARS-CoV-2 Proteins NSP4 and ORF9b Induce Mitochondrial Dysfunction and mtDNA Release

The presence of mtDNA in the circulation is attributed to robust mitochondrial damage extending from OMM to IMM. To determine the SARS-CoV-2 proteins involved in mitochondrial dysfunction and mtDNA release, the effects of all 29 SARS-CoV-2 proteins on mtDNA release were examined using immunofluorescence analysis. A549 cells were individually transfected with plasmids encoding viral proteins. The release of mtDNA in the transfected cells was examined using immunofluorescence analysis with the anti-TFAM (to label mtDNA) and anti-TOM20 (to label mitochondria) antibodies to detect the levels of mtDNA associated with the mitochondria. Compared with that in the empty vector (VEC)-transfected cells, the amount of mtDNA associated with the mitochondria was significantly lower in cells transfected with NSP4, ORF6, and ORF9b (Figure 3a). The combination of actinomycin D, ABT-737, and zVD) was used as a positive control. Transfection with NSP2, ORF6, and ORF9b decreased the mtDNA signal intensity associated with the mitochondria (Figure 3b). Similarly, the levels of Cyt c associated with the mitochondria were examined using immunofluorescence. Transfection with NSP4 and ORF9b significantly decreased the Cyt c signal associated with the mitochondria (Figure 3i,j). Next, the following two major mitochondrial functional parameters were measured using flow cytometry: mitochondrial reactive oxygen species (mtROS) and mitochondrial membrane potential (ΔΨm). Transfection with NSP2, NSP4, NSP6, NSP8, ORF3a, ORF6, and ORF9b upregulated the mtROS levels and significantly downregulated ΔΨm. Antimycin A and carbonyl cyanide 4-(trifluoromethoxy)phenylhydrazone (FCCP) were used as the positive controls for the mtROS and ΔΨm experiments, respectively (Figure S3b,c). To increase the efficiency of ectopic expression, lentiviral particles encoding the selected SARS-CoV-2 proteins with a marked adverse effect on the mitochondria were generated. The effect of these lentiviral particles was evaluated individually or in combination with mtDNA and the mitochondrial function parameters. Cells transduced with NSP4, ORF6, or ORF9b exhibited the most significant downregulation of the mtDNA signals associated with the mitochondria, Cyt c signals associated with the mitochondria (Figure 3c), and ΔΨm (Figure 3d) and upregulation of mtROS levels (Figure 3e). A similar trend was observed in the transduced cells stained with PicoGreen, which has been used previously to measure mtDNA release [24] (Figure S3d). These results indicate that compared with other SARS-CoV-2-encoded proteins, NSP4, ORF6, and ORF9b have the most prominent effect in inducing mitochondrial dysfunction and mtDNA release in airway epithelial cells (Figure 3f). Next, the combinatorial effect of NSP4, ORF6, and ORF9b on mtDNA release was evaluated. Transduction with the combination of NSP4 and ORF9b (hereafter referred to as N4 + 9b) resulted in the most marked downregulation of mtDNA signals associated with the mitochondria (Figure 3g); mtDNA appeared to be present outside the mitochondria in some of the transduced cells (Figure 3h). Additionally, transduction of cells with N4 + 9b resulted in the robust downregulation of the Cyt c signals associated with the mitochondria (Figure 3i,j). Immunoblotting of the cytosolic fraction revealed the appearance of the released Cyt c in the cytoplasm (Figure 3k). Based on these findings, NSP4 and ORF9b were selected for further experiments.

### 3.4. NSP4 and ORF9b Induce Pro-Inflammatory mtDNA Extrusion from the Cells

To investigate the effect of the released mtDNA on the induction of cytosolic DNA sensors and inflammasome formation, the expression levels of cGAS and NLRP3 were examined. Consistent with the results obtained in the SARS-CoV-2 infection experiments, the expression of these sensor proteins was not induced (Figure S4). Based on these results, the levels of mtDNA in the cytoplasm and culture supernatant were examined. The expression of TFAM (mtDNA marker) was localized to the cytosol in the N4 + 9b-transduced cells. However, the cytosolic TFAM level in the N4 + 9b-transduced cells was significantly lower than that in positive control-treated cells (Figure 3k,m). The mtDNA levels in the culture supernatant of the N4 + 9b-transduced cells were higher than those in the culture supernatant of the positive control-treated cells (Figure 3l,m). These results explain the failure of released mtDNA to induce cytosolic pathogen recognition receptors (PRRs) as most of the mtDNA appears to be secreted outside the cells.

To further examine if the TFAM observed in Figure 3i is associated with mtDNA released from the mitochondria, the culture supernatant of N4 + 9b-transduced cells was subjected to TFAM ChIP analysis (as described in Figure 1m), followed by immunoblotting analysis (Figure 3n). DNA was isolated from the immunoprecipitated complex and subjected to RT-qPCR. The number of mtDNA copies was significantly upregulated in the culture medium of the N4 + 9b-transduced cells (Figure 3o,p). This is consistent with the increased mtDNA release observed in patients with COVID-19 (Figure 1b). To examine the immunogenic response of the released mtDNA, NHBE cells were treated with various copy numbers of isolated mtDNA and the expression of *IL1B* was examined. mtDNA from N4 + 9b-transduced cells dose-dependently upregulated the *IL1B* mRNA (Figure 3q) and total IL-1β (Figure 3r) levels. Further, mtDNA-induced cell death in NHBE cells was examined using flow cytometric analysis with Sytox green. Treatment with mtDNA from the N4 + 9b-transduced cells significantly increased cell death (Figure 3s,t). These findings indicate that NSP4 and ORF9b induce the release of pro-inflammatory mtDNA, which subsequently induces the expression of pro-inflammatory IL-1β and cell death in the surrounding cells.

### 3.5. NSP4 and ORF9b Induce Mitochondrial Damage and BAX/BAK-Dependent Mitochondrial Macropore Formation

As SARS-CoV-2-infected cells exhibited robust mitochondrial structural changes and decreased mitochondrial mass (Figure 2c–g), the effect of transduction with N4 + 9b on mitochondrial morphology and mitochondrial mass was examined. N4 + 9b-transduced A549 cells were stained with MitoTracker Red (MTR) and MitoTracker Green (MTG) to analyze the mitochondrial shape and mass, respectively. At 48 h post-transduction, confocal microscopy and flow cytometry analyses were performed. Compared with the healthy elongated network’s mitochondrial morphology, in the VEC-transduced cells, robust changes in mitochondrial morphology, especially increased numbers of punctate and blob mitochondria, were observed in the N4 + 9b-transduced cells. Rotenone was used as a positive control for the experiments to evaluate the changes in mitochondrial shape (Figure S5a). Image analysis confirmed the presence of a decreased mitochondrial network in the N4 + 9b-transduced cells (Figure 4a). To visualize the changes in mitochondrial shape at a higher resolution, the transduced cells stained with the anti-TOM20 antibodies were subjected to LIGHTNING microscopy. Consistent with the previous results, the mitochondria mostly exhibited a round/donut-shaped morphology with extensive inner membrane damage (Figure 4b). The appearance of round-shaped mitochondria was accompanied by a decreased mitochondrial mass (Figure 4c).

Mitochondrial structural changes with deceased ΔΨm are often associated with mitochondrial clearance through mitophagy [51]. To determine if the decreased ΔΨm in the N4 + 9b-transduced cells was due to increased mitophagy, the expression levels of PINK1 and PARKIN in the mitochondrial fraction and the mitochondrial mass (an indicator of mitochondrial turnover) were examined. However, PINK1 accumulation and Parkin translocation to the mitochondria were not significantly affected in the N4 + 9b-transduced cells (Figure 4d). Next, a lentiviral construct of PINK1 was generated to evaluate the effect of PINK1 overexpression on mitophagy. Mitochondrial mass markedly declined in the PINK1-expressing N4 + 9b-transduced A549 cells owing to the induction of mitophagy (Figure 4e). However, the cells could not survive beyond 72 h in the culture, suggesting that PINK1 overexpression-induced mitophagy promotes cell death rather than cell survival (Figure S5b,c). Thus, PINK1 overexpression in the N4 + 9b-transduced A549 cells led to excessive mitophagy, which may not be therapeutically viable.

Mitochondrial damage is often associated with mitochondrial permeability transition pore (mPTP) opening. To investigate the opening of mPTP in the N4 + 9b-transduced cells, an mPTP assay was performed using the fluorescent probe calcein-AM. Ionomycin was used as a positive control. The calcein-AM intensity in the N4 + 9b-transduced cells was significantly lower than that in the VEC-transduced cells (Figure 4f,g). mPTP is the result of mitochondrial inner membrane permeabilization (MIMP) and mitochondrial outer membrane permeabilization (MOMP). Recent studies have demonstrated that BAX and BAK-mediated MOMP facilitates mtDNA release and is preceded by mPTP formation [24,25]. Furthermore, BAX/BAK pore formation and the subsequent mtDNA release activate the pro-inflammatory response in the cell [25]. Thus, the stabilization of mitochondrial BAK and the translocation of cytosolic BAX were examined in the N4 + 9b-transduced cells using confocal microscopy. The mitochondrial levels of BAK and BAX were upregulated in N4 + 9b-transduced cells. A similar expression pattern was observed in cells treated with macropore formation inducers (Figure 4h–k and Figure S5d,e). The upregulated BAX and BAK levels on the mitochondrial surface were further confirmed by subjecting the mitochondrial extracts to immunoblotting analysis (Figure 4l). Apoptotic stimuli promote the association between BAX and BAK on the mitochondria and consequently induce BAX/BAK macropore formation [25]. Thus, the formation of similar structures was examined in the N4 + 9b-transduced cells. BAK and BAX puncta colocalized with the mitochondrial TFAM, suggesting the formation of macropores as characterized earlier [25] (Figure 4m). The prerequisite for macropore formation is the Bcl-xL and MCL1-mediated inhibition of the apoptotic pathway. Thus, the expression levels of Bcl-xL and MCL1 (anti-apoptotic proteins) were examined. The MCL1 levels were significantly downregulated in the mitochondrial fractions, whereas the Bcl-xL levels were not affected in the N4 + 9b-transduced cells, suggesting that MCL1 is involved in macropore formation (Figure 4n).

To understand whether the mitochondrial translocation of BAX, stabilization of BAK, and MCL1 downregulation were prerequisites for mtDNA release, the expressions of BAK and BAX were knocked down using short hairpin RNA (shRNA) (shBAK and shBAX, respectively) (Figure 4o,p), and MCL1 (anti-apoptotic protein involved in mtDNA release) was upregulated using the overexpression vector [25,52] (Figure 4q). Simultaneous BAK knockdown and MCL1 overexpression markedly decreased mtDNA release from the mitochondria compared with other conditions (Figure 4r). This indicates that MCL1 has an essential role in mtDNA release along with BAX/BAK-induced macropores.

### 3.6. MCL1 Induces Inner Mitochondrial Membrane Vesicle Formation

The critical involvement of MCL1 in mtDNA release encouraged us to explore the roles of MCL1 in processes other than those involved in promoting BAX/BAK macropore formation. The effect of MCL1 knockdown (Figure S6a) on IMM/mitochondrial cristae was examined (Figure S5a). Electron microscopy (EM) imaging revealed that MCL1 knockdown resulted in extensive IMM/cristae disruption (Figure 5a,b). The mitochondrial matrix comprised vesicular structures, which were observed in most of the imaged damaged mitochondria. Next, the inner membrane stability was examined using LIGHTNING microscopy. Cells were stained with anti-TOM20 antibodies and co-transfected with an inner membrane-targeted mitochondrial green fluorescent protein (GFP) (IMM^GFP^; inner membrane reporter). MCL1 knockdown downregulated the IMM^GFP^ signal, suggesting inner membrane rupture (Figure 5c,d). The IMM^GFP^ signal was also downregulated in the healthy tubular network of the mitochondria. Additionally, the IMM^GFP^ signal in the N4 + 9b-transduced cells was lower than that in the VEC-transduced cells (Figure S6b). However, BAX/BAK shRNA-transfected groups did not exhibit marked downregulation in the IMM^GFP^ signal (Figure 5d). Cells were transfected with IMM^GFP^ and probed with the anti-DNA and anti-TOM20 antibodies to simultaneously visualize both the inner and outer membranes and mtDNA. mtDNA was always co-associated with the inner membrane-like vesicles (Figure 5e,f). The association of mtDNA with the inner membrane was further confirmed by the colocalization of IMM^GFP^ with TFAM in both the N4 + 9b-transduced and MCL1 knockdown cells (Figure 5g). Next, cells co-transfected with IMM^GFP^ and Scarlet-TFAM were subjected to live-cell imaging, which revealed small vesicular structures enclosing the mtDNA (Figure 5h). A similar degree of mtDNA association with COX-IV (inner membrane protein) was observed in the N4 + 9b-transduced and shMCL1-transduced groups (Figure S6c,d). To further evaluate the impact of these inner membrane vesicular structures, the expression of IMM^GFP^ was examined in the culture supernatant. The expression of IMM^GFP^ was detected in the culture supernatant of MCL1 knockdown and the N4 + 9b-transduced cells (Figure 5i). These results suggest that NSP4 and ORF9b mitigate the MCL1-mediated suppression of inner membrane destabilization and mtDNA release into the inner membrane vesicles (Figure 5j) in addition to facilitating BAX/BAK macropore formation on the OMM.

### 3.7. NSP4 Binds to BAK and ORF9b Interacts with MCL1 to Regulate mtDNA Release

The above-mentioned results indicate that NSP4 and ORF9b regulate macropore formation and inner membrane vesicle formation through BAX/BAK and MCL1. To elucidate the direct interaction of NSP4 and/or ORF9b with the key proteins inducing MOMP, A549 cells were individually transfected with NSP4 or ORF9b containing a Strep-II tag. The total cell lysate was prepared and subjected to NSP4 and ORF9b co-immunoprecipitation using the anti-Strep-II antibodies, followed by immunoblotting with anti-BAX, anti-BAK, anti-MCL1, and anti-Bcl-xL antibodies. BAK co-immunoprecipitated with NSP4, whereas MCL1 co-immunoprecipitated with ORF9b (Figure 6a–d). These results were further confirmed using immunofluorescence and colocalization analyses, which revealed enhanced BAK signals and the colocalization of BAK with NSP4 (Figure 6e,g). The overall MCL1 signal was downregulated with the remaining signal colocalizing with ORF9b, which was consistent with the immunoprecipitation results (Figure 6f,h). Additionally, the interaction of the NSP4 protein (N4^T492I^) found in the recently identified Omicron variant of SARS-CoV-2 with BAK was examined. However, no significant changes were observed in the association of BAK with N4^T492I^ (Figure 6e,g). Similarly, the mtDNA release was not significantly different between the N4^T492I^-transduced cells and wild-type NSP4-transduced cells (Figure 6i). NSP4 transduction significantly increased the BAK levels on the mitochondrial surface (Figure 6j,k). In contrast, ORF9b transduction significantly downregulated MCL1 expression as evidenced by the decreased signal associated with mitochondria (Figure 6l,m). Furthermore, the expression of MCL1 associated with IMM was examined. Transduction with ORF9b downregulated IMM-associated MCL1 (Figure 6n). These results suggest that the NSP4 and ORF9b of SARS-CoV-2 promote mitochondrial macropore formation and that MCL1 regulates inner membrane stability and vesicle formation. NSP4 recruits BAK to the mitochondria by directly interacting with it to form the BAX/BAK macropore (Figure 4m). Transduction of ORF9b mitigated the inhibitory effect of MCL1 on BAX/BAK macropore formation and inner membrane stability most likely by hindering the MCL1 turnover.

### 3.8. BAK Knockdown and MCL1-Overexpressing MSCs Rescue Cell Death through Functional IMT

Previously, we demonstrated that IMT rescues epithelial cell death through the donation of healthy mitochondria via TNTs [35]. To evaluate if the donated healthy mitochondria can rescue mtDNA release and cell death, A549 cells were transduced with VEC (A549^VEC^) or N4 + 9b (A549^N4+9b^) for 72 h. The transduced A549 cells were then stained with CellTracker Red (CTDR) immediately before co-culturing with MSCs expressing mito-GFP. After 24 h of co-culture, the GFP signal was detected in A549^N4+9b^ cells (indicated by the presence of both mito-GFP and CTDR (yellow)) (Figure 7a), suggesting mitochondrial uptake from MSCs. Image analysis revealed a significant increase in the mitochondrial transfer from the MSCs to A549^N4+9b^ cells (Figure 7b). Analysis of mito-GFP signals in A549 cells revealed that TNFAIP2 (which regulates TNT-mediated mitochondrial transfer) knockdown significantly inhibited IMT (Figure 7b). This indicated that mitochondrial donation from MSCs to A549 cells was dependent on TNTs. To examine whether IMT could rescue cell death, unlabeled MSCs were co-cultured with CTDR-stained A549 cells. After 24 h of co-culture, cells were stained with Sytox green and subjected to flow cytometry analysis. Compared with that in the A549^VEC^–MSC co-culture, cell death was not markedly rescued in the A549^N4+9b^–MSC co-culture (Figure 7c). Next, the effect of IMT on the metabolic function of recipient A549^N4+9b^ cells was examined. The donated mitochondria were not sufficient to decrease the mtROS levels or attenuate mitochondrial depolarization in recipient cells (Figure 7d). These results encouraged us to generate genetically engineered MSCs that can markedly rescue the metabolic defect and attenuate cell death in A549^N4+9b^ cells. MSCs were genetically engineered by targeting BAK and MCL1 (Figure 7e). BAK was knocked down (Figure S7a,b) and MCL1 was overexpressed (Figure S7c,d) in MSCs using a similar approach to the A549 cells (Figure 4o,q). These engineered MSCs (MSC^shBAK+MCL1^) retained the stem cell markers similar to the control cells (MSC^Con^). This suggests that the simultaneous BAK knockdown and MCL1 overexpression did not affect the stem cell phenotype of MSCs (Figure S7e). MSC^shBAK+MCL1^ co-cultured with A549^N4+9b^ cells exhibited enhanced mitochondrial donation to the recipient cells (Figure 7f). Representative images of IMT by MSC^shBAK+MCL1^ are shown in Figure S7f. The donated mitochondria exhibited healthy tubular morphology (Figure 7g), attenuated mtROS, and restored ΔΨm in A549^N4+9b^ cells (Figure 7h). Similarly, MSC^shBAK+MCL1^ restored mitochondrial function in NHBE cells by donating functional mitochondria (Figure 7i). Notably, the BAK expression was consistently lower in A549^N4+9b^ and MCL1 levels were increased after the co-culture with MSC^shBAK+MCL1^ (Figure 7g,h).

To validate the IMT potential of MSC^shBAK+MCL1^ in a more holistic model, SARS-CoV-2 infected cells were used (as shown in Figure 2). MSC^shBAK+MCL1^ exhibited increased mitochondrial donation to the SARS-CoV-2-infected A549 cells (Figure 7j,k). IMT from MSC^shBAK+MCL1^ restored healthy mitochondrial morphology in SARS-CoV-2-infected cells (Figure 7l). These results suggest that MSC^shBAK+MCL1^ retain their functional IMT potential and rescue SARS-CoV-2-mediated mitochondrial dysfunction in the recipient airway epithelial cells.

### 3.9. MSC^shBAK+MCL1^ Attenuate Inflammatory mtDNA Release from Airway Epithelial Cells and Rescue Cell Death

Next, the effects of the donated mitochondria on the apoptotic response and pro-inflammatory mtDNA release were examined. The co-cultures of MSCs (unlabeled) and SARS-CoV-2-infected A549 (CTDR-stained) cells were subjected to Cyt c immunostaining. The intracellular Cyt c levels in CTDR-stained SARS-CoV-2-infected A549 cells (indicating decreased Cyt c release) co-cultured with MSC^shBAK+MCL1^ were significantly upregulated compared with those in CTDR-stained SARS-CoV-2-infected A549 cells co-cultured with the non-targeted scrambled control (Srm) and VEC2 (overexpression empty vector for MCL1)-transfected MSCs (MSC^Srm+VEC2^) (Figure 8a,b). Next, the effect of MSC^shBAK+MCL1^ on mtDNA release from SARS-CoV-2-infected A549 cells was examined. Upon co-culture with MSC^shBAK+MCL1^, the levels of mtDNA associated with the mitochondria were significantly upregulated in the recipient A549^N4+9b^ cells, suggesting decreased mtDNA release from the mitochondria (Figure 8c,d). MSC^shBAK+MCL1^ mitigated A549 cell death, which was also verified using the terminal deoxynucleotidyl transferase dUTP nick-end labeling assay (TUNEL) (Figure S8a,b). To verify these findings in the A549^N4+9b^ cells, MSC^shBAK+MCL1^ were co-cultured with A549^N4+9b^. Compared with SARS-CoV-2 infection (Figure 8a,b), the MSC^shBAK+MCL1^ co-culture decreased Cyt c release (Figure 8e,f), mitigated mtDNA release (Figure 8g,h), and markedly decreased cell death (Figure S8c) in the A549^N4+9b^ cells. Similar therapeutic effects of MSC^shBAK+MCL1^ were observed upon quantification of mtDNA copies in the recipient A549^N4+9b^ cells in co-culture (Figure S8d).

To explore whether the therapeutic effect of MSC^shBAK+MCL1^ involves the mitigation of the pro-inflammatory effect on the A549^N4+9b^ cells, the pro-inflammatory and anti-apoptotic effects of the co-culture supernatant were examined (Figure 8i). As shown in Figure 8j,k, the co-culture supernatant downregulated the *IL1B* mRNA and the secreted IL-1β levels in NHBE cells. Additionally, the A549^N4+9b^–MSC^shBAX+MCL1^ co-culture supernatant attenuated NHBE cell death compared with the A549^N4+9b^–MSC^Srm+VEC2^ co-culture supernatant (Figure 8l). Thus, these results provide the first comprehensive evidence that the modulation of mitochondrial pore formation by targeting BAK activation and enhancing anti-apoptotic MCL1 expression potentiates the therapeutic efficacy of MSCs. Additionally, the donation of functional mitochondria from human MSC^shBAK+MCL1^ prevented macropore formation in the recipient SARS-CoV-2-infected or NSP4/ORF9b-expressing airway epithelial cells, which can be a potential therapeutic strategy for COVID-19. Based on their enhanced therapeutic efficacy, we named these genetically modified cells IMAT-MSCs (intercellular mitochondrial transfer-assisted therapeutic MSCs).

## 4. Discussion

SARS-CoV-2-induced mitochondrial damage is an emerging pathological determinant in COVID-19 [50,53,54,55,56,57]. Extensive mitochondrial ultrastructural changes and functional impairments are primarily observed in infected airway epithelial cells [31,58] in addition to endothelial cells [50], monocytes [54,57], and T cells [55,59]. Under some conditions, mitochondrial damage is accompanied by pathogenic mtDNA release. Several studies have demonstrated that the circulating mtDNA levels in patients with COVID-19 are positively correlated with disease severity [12,13]. The findings of this study further demonstrated the value of circulating mtDNA as a potential biomarker to predict COVID-19 severity and the pro-inflammatory response. In this study, mtDNA extracted from patients with COVID-19 elicited a pro-inflammatory response and promoted cell death in primary human airway epithelial cells. These novel findings encouraged us to elucidate the molecular mechanism of SARS-CoV-2-induced mtDNA release and develop a rational stem cell-based approach with potential clinical applications for the treatment of other diseases in addition to COVID-19.

The extrusion of mtDNA from the mitochondria is reported in various inflammatory and infectious diseases [10,11]. However, the molecular mechanism underlying mtDNA release under pathological conditions, especially SARS-CoV-2 infection, has not yet been elucidated. This study, for the first time, elucidated the molecular pathway involved in SARS-CoV-2 infection-induced mtDNA release. Mechanistically, the SARS-CoV-2-encoded proteins NSP4 and ORF9b synergistically induce mtDNA release. NSP4 stabilizes BAK on the outer membrane through direct interaction. Subsequently, BAK recruits BAX to induce outer membrane macropore formation. These BAX/BAK macropores were previously reported to be formed upon exposure to apoptotic stimuli, usually under caspase inhibition conditions [24,25]. Similarly, infections, such as severe fever with thrombocytopenia syndrome virus infections, can induce BAX/BAK-dependent mtDNA release [26]. This is the first study to demonstrate that SARS-CoV-2 mediates the formation of these macropores in target airway epithelial cells. Other have also shown an intricate relationship between SARS-CoV-2 and mitochondrial function. Notably, a comprehensive study from the Madesh lab has elucidated the molecular basis of SARS-CoV-2-mediated autophagy in cardiomyocytes [22]. The authors have generated a comprehensive map of the SARS-CoV-2-encoded proteins interacting with various mitochondrial resident proteins and thereby inducing mitochondrial fragmentation, calcium imbalance, and cell death. Flynn et al. performed a screening using an integrated system of clustered regularly interspaced palindrome repeat-caspase (CRISPR-Cas) and the comprehensive identification of RNA-binding proteins by mass spectrometry and reported the localization of SARS-CoV-2 RNA with mitochondrial proteins. In particular, MRM2 was highly enriched with the SARS-CoV-2 RNA fractions [60]. Other studies have also reported the mitochondrial localization of SARS-CoV-2 RNA [23], which was proposed to be mediated by TOM20. These findings suggest that SARS-CoV-2 infection is associated with extensive mitochondrial dysfunction.

In addition to outer membrane macropore formation, mtDNA release from the mitochondrial matrix is dependent on overcoming the inner membrane barrier. MIMP was speculated to be a passive event that is initiated after macropore formation. This theory postulated that the inner membrane ruptures upon exposure to the cytosol after outer membrane macropore formation, leading to the release of mtDNA into the cytosol [24,25]. This study provided the first molecular evidence that mtDNA release from the inner membrane is not a passive event and that it is a well-coordinated event regulated by MCL1. EM, immunofluorescence, and biochemical analyses revealed that *MCL1* inhibition induces inner membrane vesicle formation. Genetically overexpressing MCL1 prevents inner membrane damage and mtDNA release. The effect of MCL1 on inner membrane vesicle formation was further validated in cells expressing ORF9b. ORF9b induced inner membrane vesicle formation in transfected airway epithelial cells by directly interacting with MCL1, which prevented the accumulation of MCL1. The distinct roles of MCL1 have been previously reported. MCL1 localized to the outer membrane suppresses the apoptotic signal, whereas that localized to the matrix aids in the maintenance of inner membrane integrity and the cristae ultrastructure and prevents mPTP formation [52,61]. However, this study proposed a novel mechanism through which MCL1 regulates inner membrane vesicle formation and subsequent mtDNA packaging into these vesicles. Mitochondrion-derived vesicles were previously characterized as comprising only the outer membrane or both inner and outer membranes [62]. However, the findings of this study suggest that vesicles enclosing mtDNA are predominantly derived from the inner membrane and are subsequently extruded via BAX/BAK macropores on the outer membrane. A comprehensive characterization of these vesicles and their excision mechanism will provide useful insights into this phenomenon, which must be explored in future studies.

The release of mtDNA into the cytosol or the extracellular space is intricately associated with the activation of different PRRs [63,64]. Upon release into the cytosol, mtDNA activates the cGAS-STING1 pathway, which activates the type I interferon response [29,30]. The activation of the cGAS-STING pathway may be beneficial during viral infections and potentiate the anti-viral activity of immune cells [29,30] or result in pathological consequences in conditions, such as autoimmune diseases [65]. In SARS-CoV-2 infection, mtDNA released from endothelial cells activates the pro-inflammatory response through the cGAS-STING pathway [50]. However, the findings of this study indicate that in the airway epithelial cells, mtDNA release is not coupled with intrinsic cGAS-STING activation or NLRP3 inflammasome formation. This study demonstrated that a large pool of released mtDNA is secreted extracellularly via inner membrane vesicles. Thus, mtDNA extruded from the mitochondria is protected from cytosolic DNA sensors, leading to the suppression of an immediate anti-viral interferon response. This may explain the impaired interferon response reported in patients with COVID-19 [32,66]. The presence of a minor percentage of mtDNA released without encapsulation in IMM vesicles cannot be ruled out, although this minor population may not be adequate to activate cytosolic DNA sensors. These effects of mtDNA release and the underlying mechanisms may be cell-type-specific. SARS-CoV-2-infected endothelial cells are reported to activate the cGAS/STING pathway [50]. The immediate activation of the NLRP3 pathway in the infected cells can also be ruled out. This supports the notion that the SARS-CoV-2-infected cells do not undergo immediate cell death, which may otherwise limit the propagation of the virus in the target cells.

The extracellularly released mtDNA can exert its effect on the neighboring cells by activating the TLR9 signaling pathway [13,67,68], which can trigger downstream signaling pathways, leading to the induction of PICs and chemokines. cf-mtDNA derived from the plasma of patients with COVID-19 induced a robust pro-inflammatory response and subsequently promoted cell death in primary airway epithelial cells. Furthermore, mtDNA extracted from N4 + 9b-transduced cells exerted similar pro-inflammatory and pro-apoptotic effects on airway epithelial cells. Based on these findings, we propose a new model of mtDNA release during SARS-CoV-2 infection. According to this model, the mtDNA avoids immediate exposure to cytosolic DNA sensors, leading to the inhibition of the protective anti-viral signaling of host cells. During mtDNA packaging into the inner membrane vesicles, a robust systemic pro-inflammatory response is elicited. Consistent with the findings of this study, previous studies [12,13] have reported a strong positive correlation between cf-mtDNA release and pro-inflammatory molecules in clinical serum samples of patients with COVID-19. Thus, the kinetics of these events must be evaluated to establish a correlation between the release of mtDNA (both from the mitochondria and extracellularly) and cell death during SARS-CoV-2 infection in a time-dependent manner. The effect of extracellularly released mtDNA on bystander cells and a similar phenomenon during infection by other viruses must be investigated in the future. Further studies are needed to explore whether the canonical TLR9 signaling or other pathways are activated by the extracellular mtDNA.

To devise a novel approach for therapeutic intervention based on the findings of this study, a clinically approved approach was utilized to mitigate SARS-CoV-2-induced mitochondrial damage and mtDNA release. According to the strategy employed in this study, MSCs were engineered to become resilient to macropore formation and inner membrane vesicle excision. Based on the results in airway epithelial cells, *BAK* was knocked down and MCL1 was overexpressed in MSCs using the lentiviral-based gene delivery system. Upon co-culture with SARS-CoV-2-infected or N4 + 9b-transduced cells, genetically engineered MSCs (IMAT-MSCs) exhibited enhanced therapeutic potential by mitigating mitochondrial damage and pro-inflammatory mtDNA release in the recipient cells. Mechanistic studies revealed that IMAT-MSCs donated functional mitochondria to the recipient cells via TNTs. Previously, we had reported that TNTs act as molecular connecting bridges for IMT between MSCs and airway epithelial cells [35]. This finding has also been reported in different cell types by other studies [40]. The IMT ability of MSCs is a relatively new paradigm that explains the beneficial effects of MSC transplantation. Recent studies have demonstrated that the phenomenon of IMT is conserved across different cell types both in vitro and in vivo, which provides an opportunity to devise new-generation targeted stem cell-based therapies. Our mechanistic study of the therapeutic effect of MSCs was confined to an in vitro model system which sometimes may not recapitulate the in vivo disease condition and the physiological response. Thus, to strengthen these findings, the therapeutic effect of these engineered MSCs should be evaluated in appropriate in vivo models.

MSCs have exhibited enormous translational potential for the treatment of various respiratory conditions. Successful clinical outcomes of MSC transplantation in H7N9-induced acute respiratory distress syndrome [69,70] and other respiratory diseases [70] have encouraged the therapeutic application of these cells for COVID-19, which is associated with similar complications. Currently, more than 100 clinical trials evaluating the efficacy of MSCs for the treatment of COVID-19 are enlisted (ClinicalTrials.gov). Recently, several groups have published clinical trial results evaluating the safety and efficacy of MSCs for COVID-19. The results of phase 1 clinical trials, which were published in early 2020, demonstrated the safety of MSC transplantation and reported that the mortality rate was significantly low among patients with COVID-19 receiving MSC transplantation [71] (and reviewed in [72]). Consistently, phase II trial results were also promising [73] with a recent 1-year follow-up showing long-term benefits [72]. These clinical results indicate that MSCs will provide a viable long-term alternative therapeutic option to decrease COVID-19-associated mortality. Therefore, the genetic modulation of these cells (as demonstrated in this study) to develop next-generation MSC therapy will have applications for treating other diseases in addition to COVID-19.

In summary, the findings of this study have immediate clinical applications, especially considering the ability of new emerging SARS-CoV-2 variants to evade vaccine-mediated immunity. Based on the beneficial effects of MSCs, the Food and Drug Administration (FDA) has recently approved the first MSC (modulated to express TACR1)-based therapy for COVID-19.

## 5. Limitations of Study and Future Directions

The major limitation of this study was that the safety and efficacy of IMAT-MSCs were not evaluated in preclinical or clinical settings. However, similar lentiviral-based cellular therapies are reported to be safe and have yielded promising clinical outcomes. One such approach is the chimeric antigen receptor (CAR) T-cell therapy. The currently FDA-approved CAR T-cell products are generated using a similar third-generation lentiviral system to deliver the CAR genes into the target cells. Thus, we believe that IMAT-MSCs should not have safety concerns. However, comprehensive preclinical and clinical assessments are warranted, which is beyond the scope of this study. The other limitation of this study was the limited availability of SARS-CoV-2 samples for detailed molecular studies and the limited number of Omicron variant samples for evaluation of mtDNA levels owing to the low number of hospitalizations during the third wave (January 2022 to March 2022) of COVID-19 in India. Further, most of the study has been conducted using an imaging-based approach such as immunofluorescence with a selected cell population. We used multiple replicates and the experiments were performed more than three times to avoid any bias in image selection and quantitation. Even then, biochemical assays and other sensitive assays, such as flow cytometry where a large population of cells can be evaluated, will complement some of our experimental results.

## Data Availability

There is no data associated with the manuscript as source data for the repository.

## Supplementary Materials

The following supporting information can be downloaded at: https://www.mdpi.com/article/10.3390/cells11192969/s1, Figure S1: COVID-19 patient plasma contains pro-inflammatory mtDNA. (a) Heat map showing RT-qPCR results of S gene to be absent in swab samples from patients suspected to be infected with omicron variant. The samples were positive for other SARS-CoV-2 genes like N and RNA-dependent RNA polymerase (RdRP) with RNAseP as internal control. PC indicates positive control, which are S gene positive samples (b) Relative mRNA expression of IL1B with respect to ACTB in NHBE cells incubated with plasma from COVID-19-ve and COVID-19+ve patients, respectively (n = 8). (c) Representative flow cytometry histograms showing cell death in plasma treated NHBE cells, as observed with SYTOX green staining. Numbers indicate % cell death. (d) Representation of the flow cytometry data in panel c as % cell death. (e) Representative gel picture showing the expression of ND2 gene (representing mtDNA), as observed after performing PCR of ChIP samples. G1-G8 represents groups, with each group containing pooled plasma from 10 patients. (f) Relative mRNA expression of IL6 with respect to ACTB in NHBE cells incubated with various concentrations of mtDNA (n = 6). (g) Quantitative analysis of IL-6 in the total protein extracted from NHBE cells treated with increasing concentrations of mtDNA, as measured by ELISA. Data is represented in picograms/mL (n = 6). (h) Representative flow cytometry histograms showing cell death in mtDNA treated NHBE cells, as observed with SYTOX green staining. Numbers indicate % cell death. (i) Representation of the flow cytometry data in panel c as % cell death (n = 4). (j) Quantitative analysis of cytochrome c (Cyt c) in the plasma of COVID-19-VE (20) and COVID-19+VE (30) patients, as measured by ELISA. Data is represented in nanograms/mL. All data are represented as mean ± SEM of three independent experiments, analyzed by non-parametric *t* test using Graphpad Prism software. *** *p* < 0.001; **** *p* < 0.0001; ns: not significant. Figure S2: SARS-CoV-2 causes mitochondrial damage. (a) Representative LIGHTNING microscopy images showing changes in mitochondrial morphology, as visualized by anti-TOM20 staining in SARS-CoV-2 infected A549 cells. The insets of these images are shown in Figure 2e. (b) Complete image panels of the representative images shown in Figure 2j. (c,d) Representative images showing cGAS (green) and NLRP3 (red) expression in A549 cells infected with SARS-CoV-2. DAPI (blue) was used to label nuclei. (e) Quantitative analysis of images in panel c and d (n = 14). Data is represented as the integrated density of the signal of respective proteins. All data are represented as mean ± SEM of three independent experiments, analyzed by non-parametric t test using Graphpad Prism software. * *p* < 0.05. Scale bars: a, b: 10 μm; c, d: 50 μm. Figure S3: SARS-CoV-2 proteins NSP4 and ORF9b induce release of mtDNA. (a) Heat map showing the cytochrome c (Cyt c) levels associated with mitochondria when A549 cells were individually transfected with the 29 SARS-CoV-2 plasmids individually and stained with anti-Cyt c antibody. Images were taken 24 h post-transfection, followed by image analysis. A combination of ActD+ABT+zVD (2h) was used as positive control for mtDNA release. (b) Representative flow cytometry histograms showing measurement of mtROS and (c) ΔΨm by staining the cells with TMRE and mitoSOX, respectively. Antimycin C (AntC) was used as positive control for mtROS, and FCCP for ΔΨm. (d) Representative images showing co-localization of picogreen stained mtDNA and mitotracker red stained mitochondria (MTR; pseudocolored as green), indicating mtDNA associated with mitochondria in A549 cells transduced with lentivirus encoded SARS-CoV-2 proteins as shown in Figure 3b. Data is represented as mean ± SEM of three independent experiments. Scale bar: 10 μm. Figure S4: NSP4 and ORF9b of SARS-CoV-2 does not induce expression of cytosolic DNA sensors. Representative immunoblot showing cGAS and NLRP3 expression in A549 cells transduced with N4+9b for 48h, followed by cell lysis and total protein extraction. Anti-cGAS and anti-NLRP3 antibodies were used to detect cGAS and NLRP3 respectively. A combination of ActD+ABT+zVD (2h) was used as positive control to induce the expression of these DNA sensors. Figure S5: NSP4 and ORF9b lead to mitochondrial structural changes and macropore formation. (a) Representative images showing mitochondrial shape changes in cells transduced with VEC or N4+9b, as observed with mitotracker red (MTR; pseudocolored as green) with images analysis shown in Figure 4a. Rotenone (Rot) was used as a positive control to induce mitochondrial shape changes. (b) Representative flow cytometry histograms showing cell death, as measured by SYTOX green staining. A549 cells were co-transduced with N4+9b with or without PINK1 lentiviral expression vector (PINK1pLV). VEC1 represents the backbone construct for N4 and 9b plasmids, while VEC2 represents the backbone construct for PINK1 plasmid. Numbers indicate cell death percentage. (c) Cell death represented as % cell death analyzed from the histograms shown in panel b (n = 6). (d) Representative images showing BAK and (e) BAX (red) associated with anti-TOM20 stained mitochondria (green) in A549 cells transduced either with VEC or N4+9b. A combination of ActD+ABT+zVD (2h) was used as positive control. These images represent all the panels of images shown in Figure 4h,j. Data is represented as mean ± SEM of three independent experiments, analyzed by non-parametric t test, * *p* < 0.05; *** *p* < 0.001. Scale bars: 10 μm. Figure S6: MCL1 downregulation and NSP4/ORF9b induce inner membrane disruption. (a) RT-qPCR data showing relative fold change expression of MCL1 upon transduction of A549 cells with Scramble (Srm) or shRNA targeting MCL1, followed by RNA extraction and RT-qPCR analysis (n = 6). (b) Representative images showing rupture of inner mitochondrial membrane of A549 cells transduced with N4+9b, stained with inner membrane targeted GFP (IMMGFP) and anti-TOM20 to probe outer membrane. (c) Representative images showing of COX-IV expression in cells transduced with N4+9b, or (d) MCL1 shRNA, and stained with anti-TOM20. All data are represented as mean ± SEM of three independent experiments, analyzed by non-parametric *t* test, **** *p* < 0.0001. Scale bars: 10 μm. Figure S7: BAK downregulation and MCL1 overexpression in MSCs increases intercellular mitochondrial transfer. (a) RT-qPCR data showing relative fold change expression of BAK upon transduction of A549 cells with Scramble (Srm) or shRNA targeting BAK. (b) Similarly, immunofluorescence was done followed by quantitive analysis of the images which indicate knockdown of BAK. (c,d) Lentiviral encoded VEC or MCL1 overexpression in MSCs (MCL1pLV), followed by either RNA extraction and RT-qPCR analysis (n = 6). (c) or immunofluorescence analysis of the images (d). (e) Representative flow cytometry histograms showing no change in the stem cell phenotype upon dual modification of MSCs by MCL1 overexpression and BAK knockdown by shRNA. (f) Representative images showing N4+9b transduced A549 stained with CTDR (red) and co-cultured with MSCs expressing mitoGFP (green). The images represent the quantitative data shown in Figure 7f. (g,h) Quantitive analysis of the images by specifically measuring the fluorescence signal in A549 cells after co-culture with MSCSrm+V2 or MSCshBAK+MCL1. The data represents the signal of BAK on mitochondria (g) or MCL1 on mitochondria (h). A549 cells were transduced with vector or N4+9b before co-culture. All data are represented as mean ± SEM of three independent experiments, analyzed by non-parametric t test, **** *p* < 0.0001. Scale bar: 20 μm. Figure S8: MSCs modulated with BAK shRNA and MCL1 overexpression plasmid attenuate cell death in A549 cells. (a) Representative images showing cell death by fluorescent TUNEL assay (red) in A549 cells. SARS-CoV-2 infected cells were co-cultured with MSCs expressing either scramble vector (Srm) and vector (V2) or dual transduction of BAK-shRNA and MCL1 overexpression plasmid, and stained with cell tracker green (CTG), followed by TUNEL staining (red). CTG stained dead cells appear yellow. (b) Quantitative analysis of TUNEL and CTG dual positive cells were counted and plotted as no. of A549 dead cells (n = 12). (c) Quantitative analysis of TUNEL and CTG dual positive cells done similar to panel b except that instead of SARS-CoV-2 infection, A549 cells were transduced with N4+9b (n = 14). (d) PCR analysis showing mtDNA copy number in the cell supernatant of co-culture of A549 cells and MSCs. A549 were transduced with N4+9b, whereas MSCs were modulated with BAK shRNA and MCL1 overexpression plasmid (n = 7). All data are represented as mean ± SEM of three independent experiments, analyzed by non-parametric *t* test, *** *p* < 0.001; **** *p* < 0.0001. Scale bar: 100 μm. Figure S9: Source data of all the blots. The original full blots are shown for the representative figures shown in Main and Supplementary [74,75].
