# Supplementary material for: NSP4 and ORF9b of SARS-CoV-2 Induce Pro-Inflammatory Mitochondrial DNA Release in Inner Membrane-Derived Vesicles
Source: Cells. 2022 Sep 23;11(19):2969. doi: 10.3390/cells11192969 (PMC9561960; doi:10.3390/cells11192969)
Supplement: Supplementary file 1 [file cells-11-02969-s001.zip › Figure S1.pptx]

## Slide 1
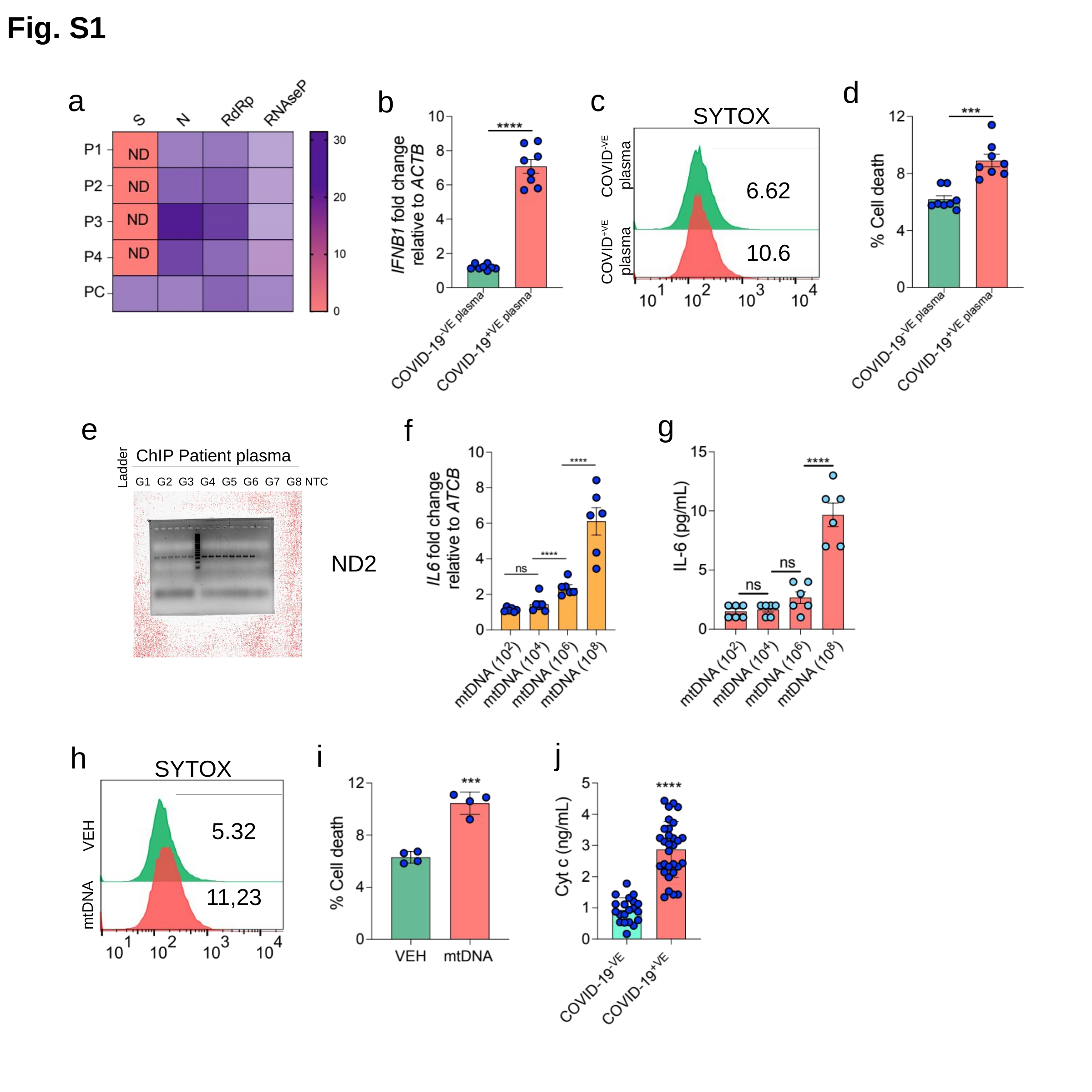

Fig. S1
d
a
c
b
SYTOX
6.62
10.6
COVID-VE
plasma
COVID+VE
plasma
g
e
f
ChIP Patient plasma
Ladder
G1 G2 G3 G4 G5 G6 G7 G8 NTC
ND2
j
i
h
SYTOX
5.32
11,23
VEH
mtDNA
