# Supplementary material for: NSP4 and ORF9b of SARS-CoV-2 Induce Pro-Inflammatory Mitochondrial DNA Release in Inner Membrane-Derived Vesicles
Source: Cells. 2022 Sep 23;11(19):2969. doi: 10.3390/cells11192969 (PMC9561960; doi:10.3390/cells11192969)
Supplement: Supplementary file 1 [file cells-11-02969-s001.zip › Figure S2.pptx]

## Slide 1
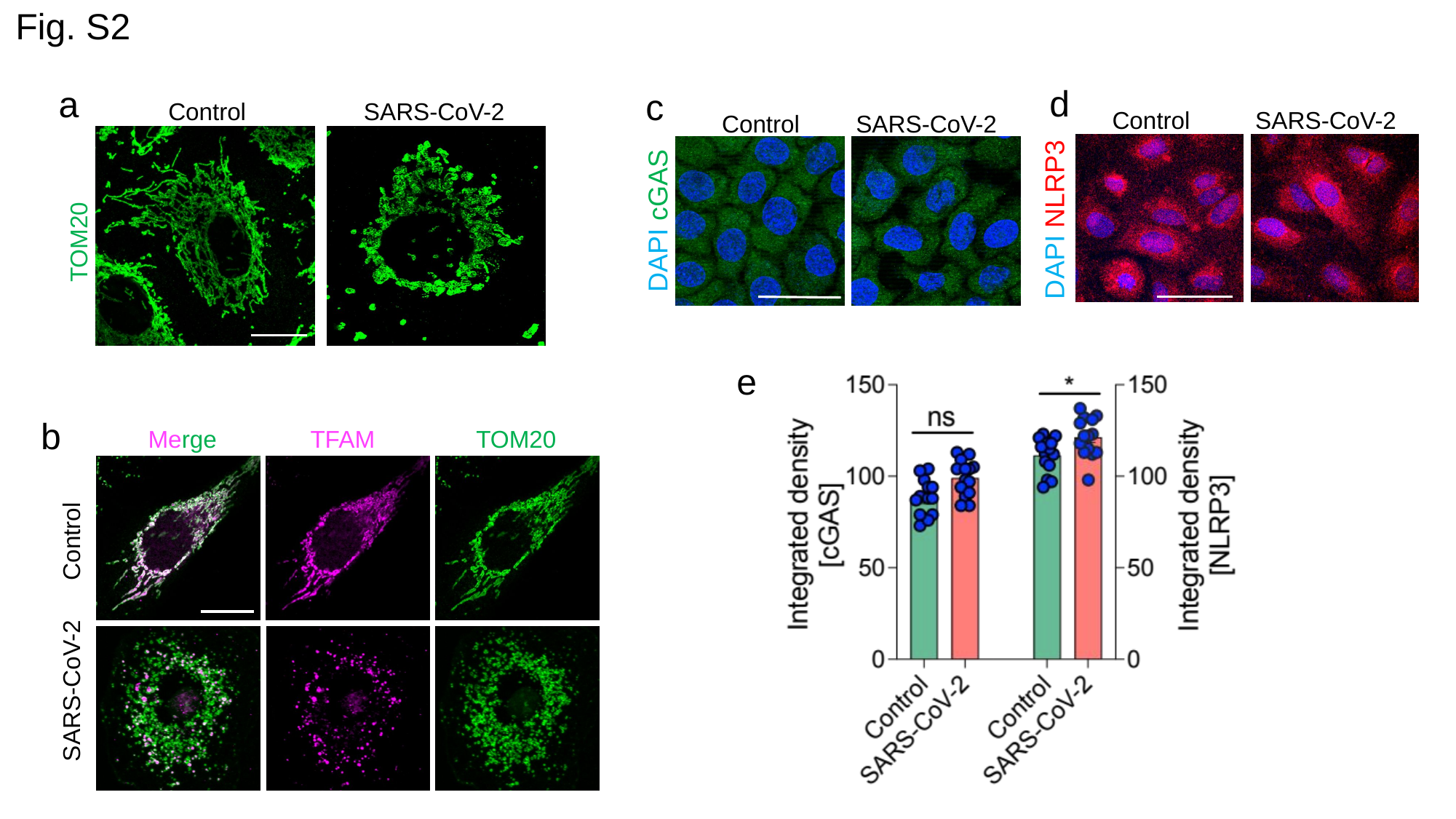

Fig. S2
d
a
c
Control
SARS-CoV-2
TOM20
Control
SARS-CoV-2
Control
SARS-CoV-2
DAPI NLRP3
DAPI cGAS
e
b
Merge TFAM TOM20
Control
SARS-CoV-2
