# Supplementary material for: NSP4 and ORF9b of SARS-CoV-2 Induce Pro-Inflammatory Mitochondrial DNA Release in Inner Membrane-Derived Vesicles
Source: Cells. 2022 Sep 23;11(19):2969. doi: 10.3390/cells11192969 (PMC9561960; doi:10.3390/cells11192969)
Supplement: Supplementary file 1 [file cells-11-02969-s001.zip › Figure S3.pptx]

## Slide 1
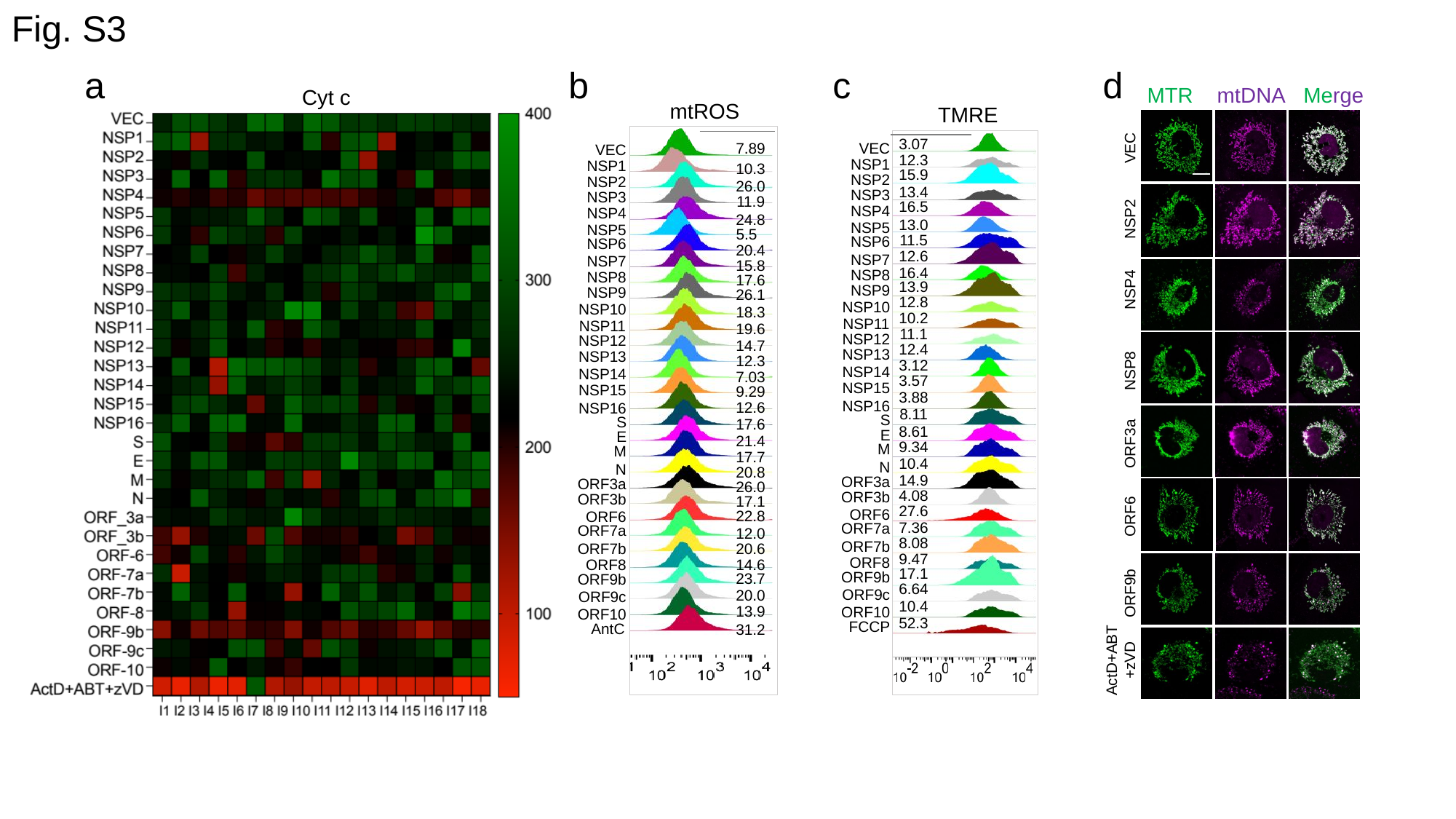

Fig. S3
a
b
c
d
MTR mtDNA Merge
VEC
NSP2
NSP4
NSP8
ORF3a
ORF6
ORF9b
ActD+ABT
+zVD
Cyt c
mtROS
7.89
10.3
26.0
11.9
24.8
5.5
20.4
15.8
17.6
26.1
18.3
19.6
14.7
12.3
7.03
9.29
12.6
17.6
21.4
17.7
20.8
26.0
17.1
22.8
12.0
20.6
14.6
23.7
20.0
13.9
31.2
VEC
NSP1
NSP2
NSP3
NSP4
NSP5
NSP6
NSP7
NSP8
NSP9
NSP10
NSP11
NSP12
NSP13
NSP14
NSP15
NSP16
S
E
M
N
ORF3a
ORF3b
ORF6
ORF7a
ORF7b
ORF8
ORF9b
ORF9c
ORF10
AntC
TMRE
VEC
NSP1
NSP2
NSP3
NSP4
NSP5
NSP6
NSP7
NSP8
NSP9
NSP10
NSP11
NSP12
NSP13
NSP14
NSP15
NSP16
S
E
M
N
ORF3a
ORF3b
ORF6
ORF7a
ORF7b
ORF8
ORF9b
ORF9c
ORF10
FCCP
3.07
12.3
15.9
13.4
16.5
13.0
11.5
12.6
16.4
13.9
12.8
10.2
11.1
12.4
3.12
3.57
3.88
8.11
8.61
9.34
10.4
14.9
4.08
27.6
7.36
8.08
9.47
17.1
6.64
10.4
52.3
