# Supplementary material for: NSP4 and ORF9b of SARS-CoV-2 Induce Pro-Inflammatory Mitochondrial DNA Release in Inner Membrane-Derived Vesicles
Source: Cells. 2022 Sep 23;11(19):2969. doi: 10.3390/cells11192969 (PMC9561960; doi:10.3390/cells11192969)
Supplement: Supplementary file 1 [file cells-11-02969-s001.zip › Figure S5.pptx]

## Slide 1
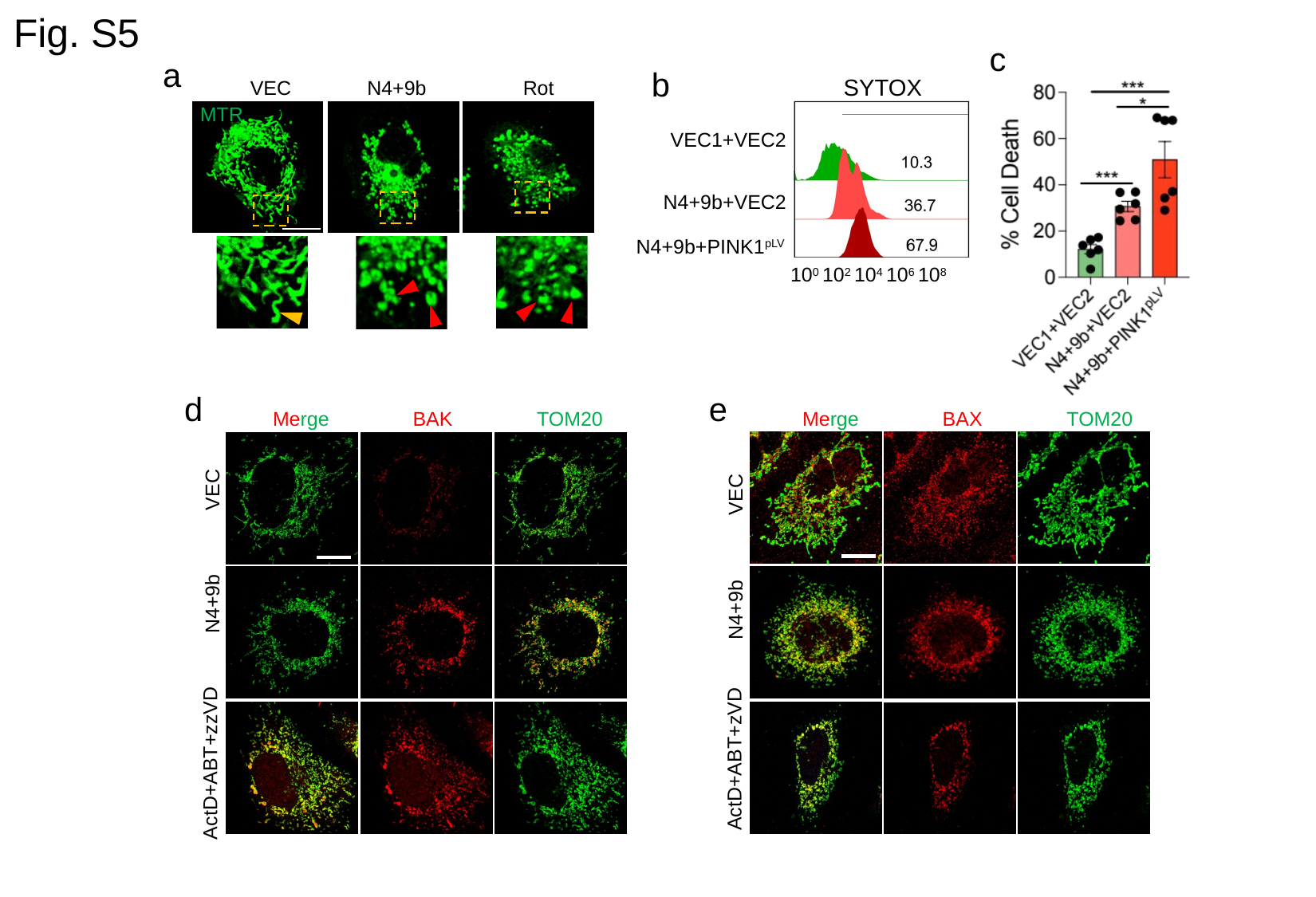

Fig. S5
c
a
b
SYTOX
100 102 104 106 108
VEC1+VEC2
N4+9b+VEC2
N4+9b+PINK1pLV
N4+9b
Rot
VEC
MTR
d
e
Merge BAK TOM20
VEC
 N4+9b
ActD+ABT+zzVD
Merge BAX TOM20
VEC
 N4+9b
ActD+ABT+zVD
