# Supplementary material for: NSP4 and ORF9b of SARS-CoV-2 Induce Pro-Inflammatory Mitochondrial DNA Release in Inner Membrane-Derived Vesicles
Source: Cells. 2022 Sep 23;11(19):2969. doi: 10.3390/cells11192969 (PMC9561960; doi:10.3390/cells11192969)
Supplement: Supplementary file 1 [file cells-11-02969-s001.zip › Figure S7.pptx]

## Slide 1
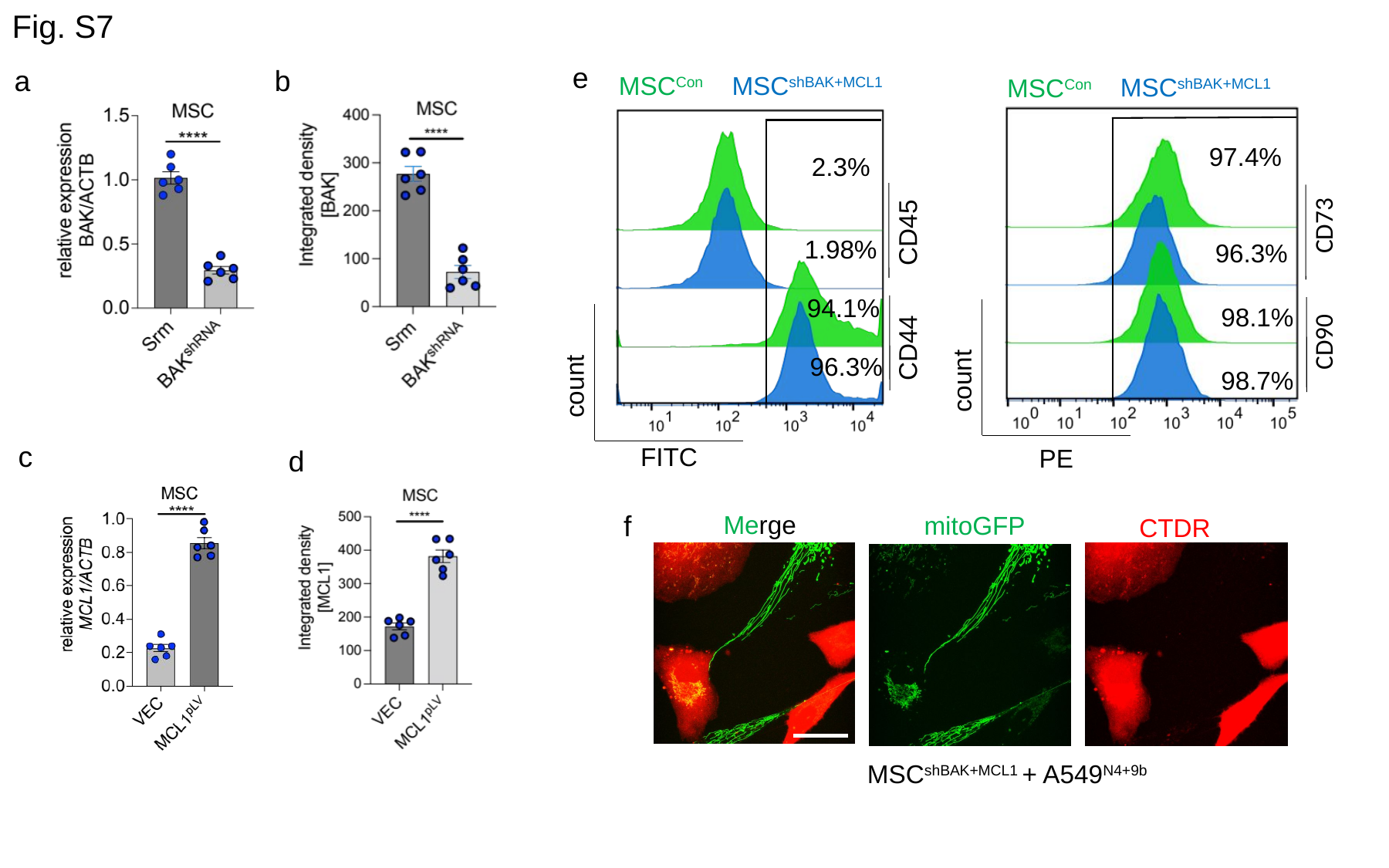

Fig. S7
e
a
b
MSCshBAK+MCL1
MSCCon
2.3%
CD45
1.98%
94.1%
CD44
96.3%
count
FITC
MSCshBAK+MCL1
MSCCon
97.4%
CD73
96.3%
98.1%
CD90
98.7%
count
PE
c
d
f
Merge
mitoGFP
CTDR
MSCshBAK+MCL1 + A549N4+9b
