# Supplementary material for: NSP4 and ORF9b of SARS-CoV-2 Induce Pro-Inflammatory Mitochondrial DNA Release in Inner Membrane-Derived Vesicles
Source: Cells. 2022 Sep 23;11(19):2969. doi: 10.3390/cells11192969 (PMC9561960; doi:10.3390/cells11192969)
Supplement: Supplementary file 1 [file cells-11-02969-s001.zip › Figure S9.pptx]

## Slide 1
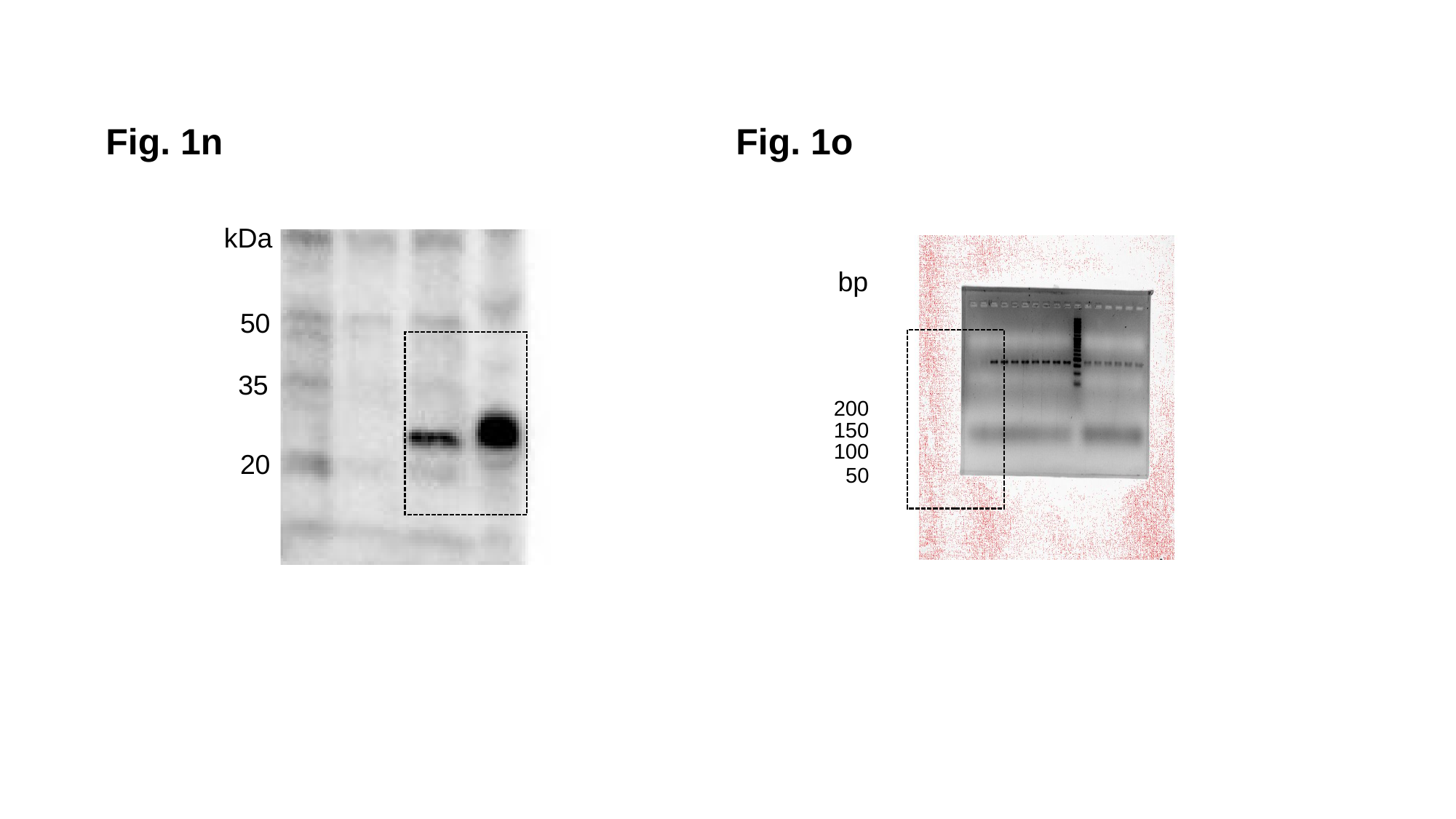

Fig. 1n
Fig. 1o
kDa
bp
50
35
200
150
100
20
50

## Slide 2
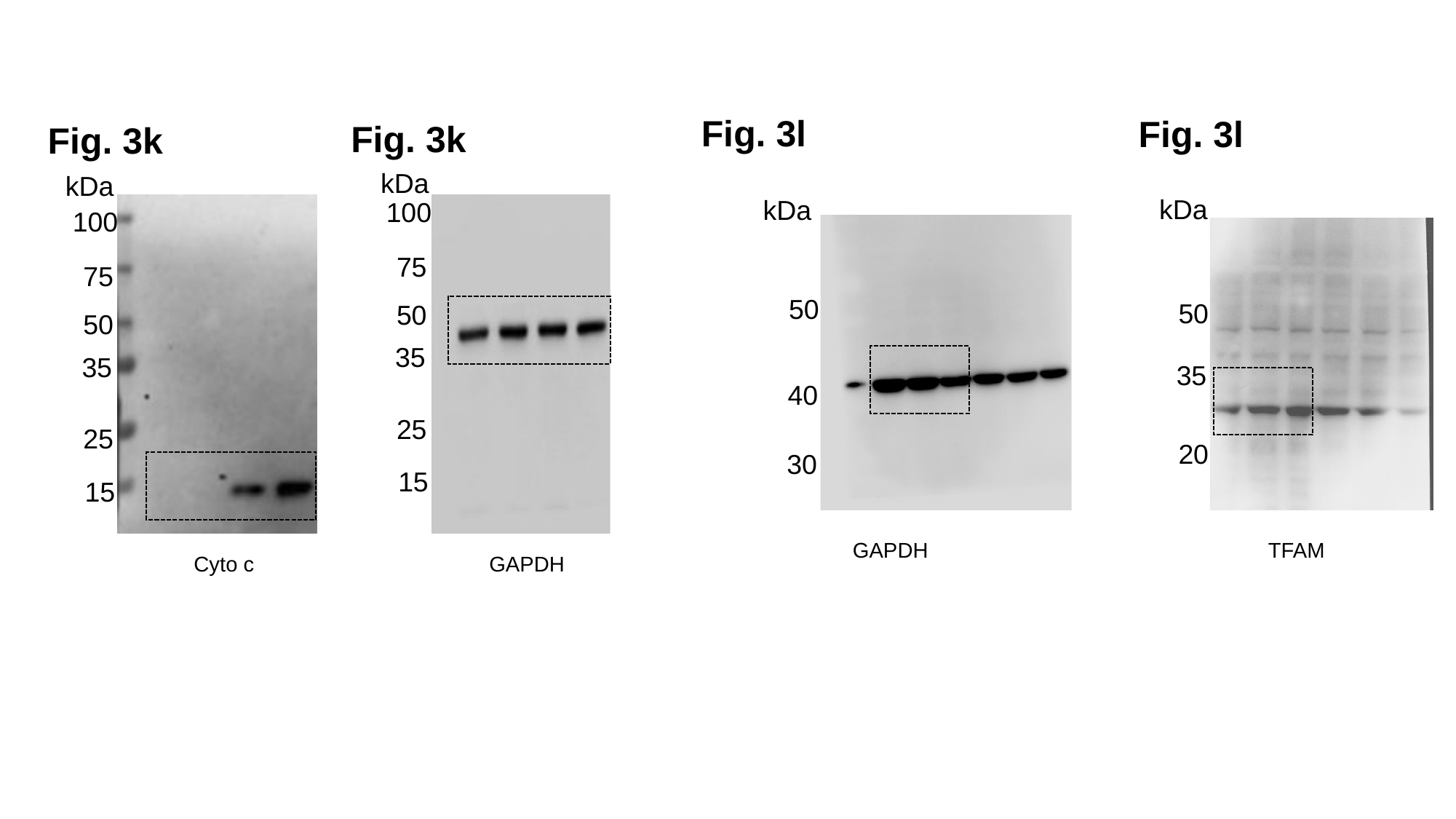

Fig. 3l
Fig. 3l
Fig. 3k
Fig. 3k
kDa
kDa
kDa
kDa
100
100
75
75
50
50
50
50
35
35
35
40
25
25
20
30
15
15
TFAM
GAPDH
Cyto c
GAPDH

## Slide 3
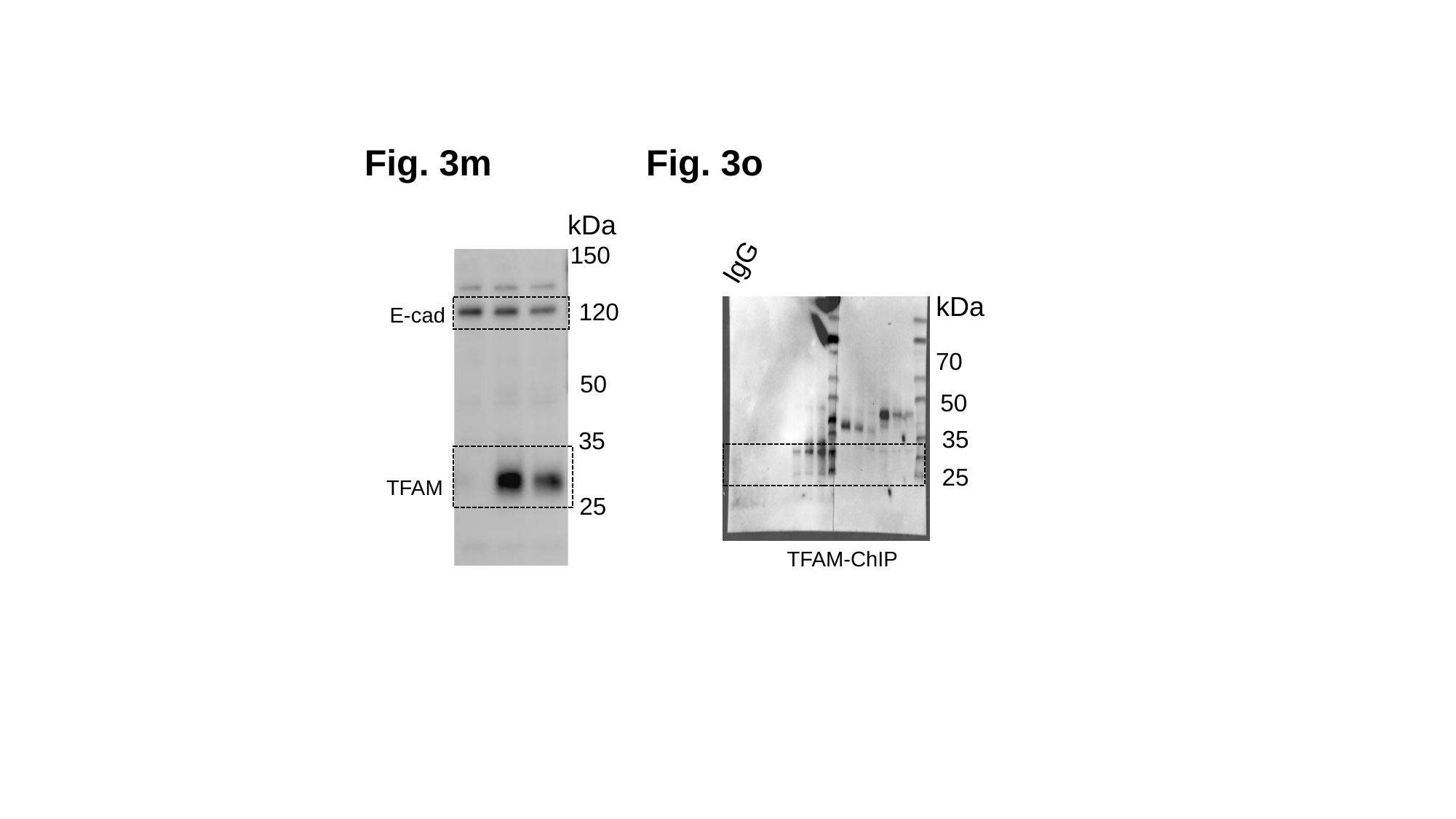

Fig. 3o
Fig. 3m
kDa
IgG
150
kDa
120
E-cad
70
50
50
35
35
25
TFAM
25
TFAM-ChIP

## Slide 4
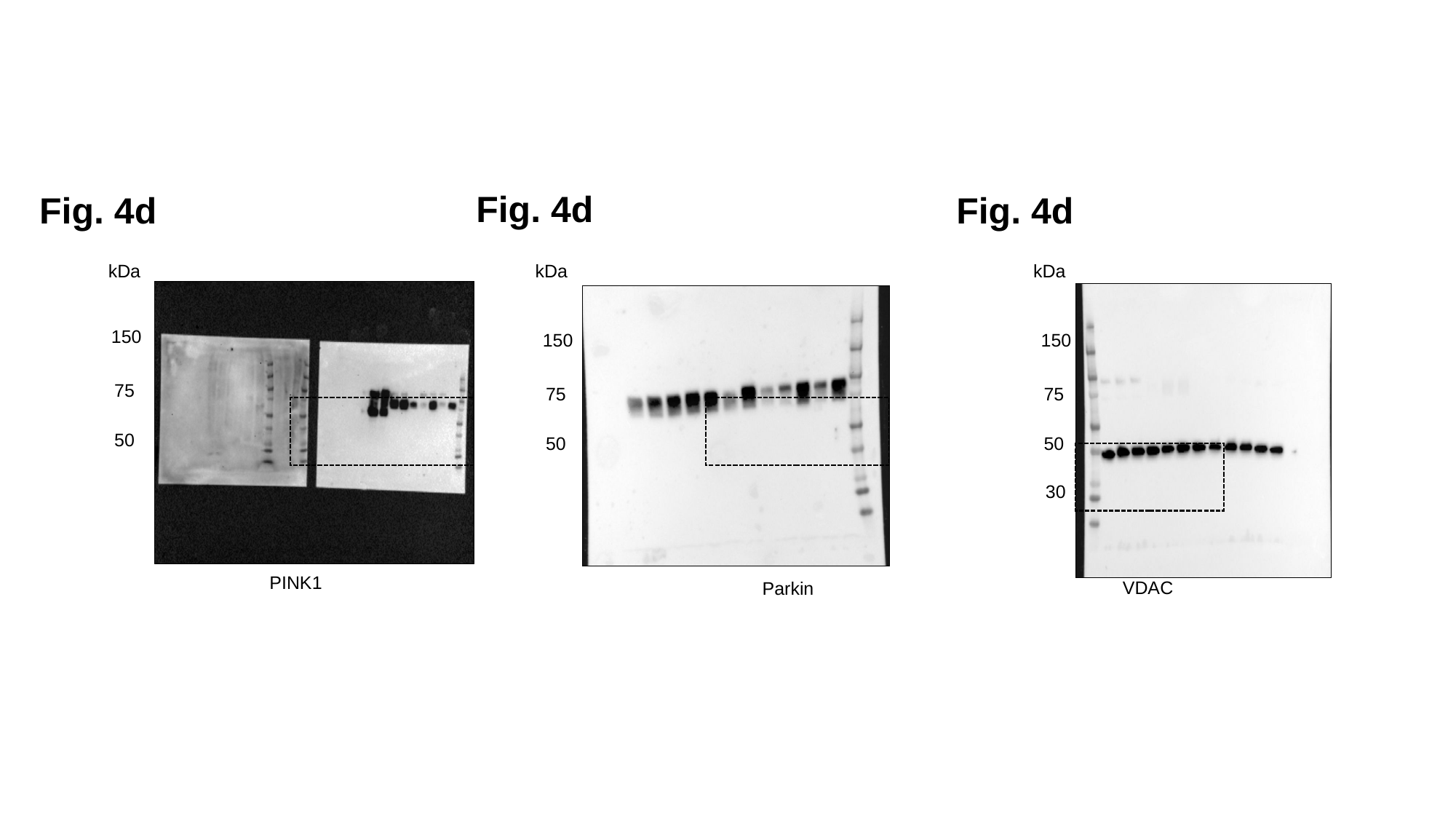

Fig. 4d
Fig. 4d
Fig. 4d
kDa
kDa
kDa
150
150
150
75
75
75
50
50
50
30
PINK1
VDAC
Parkin

## Slide 5
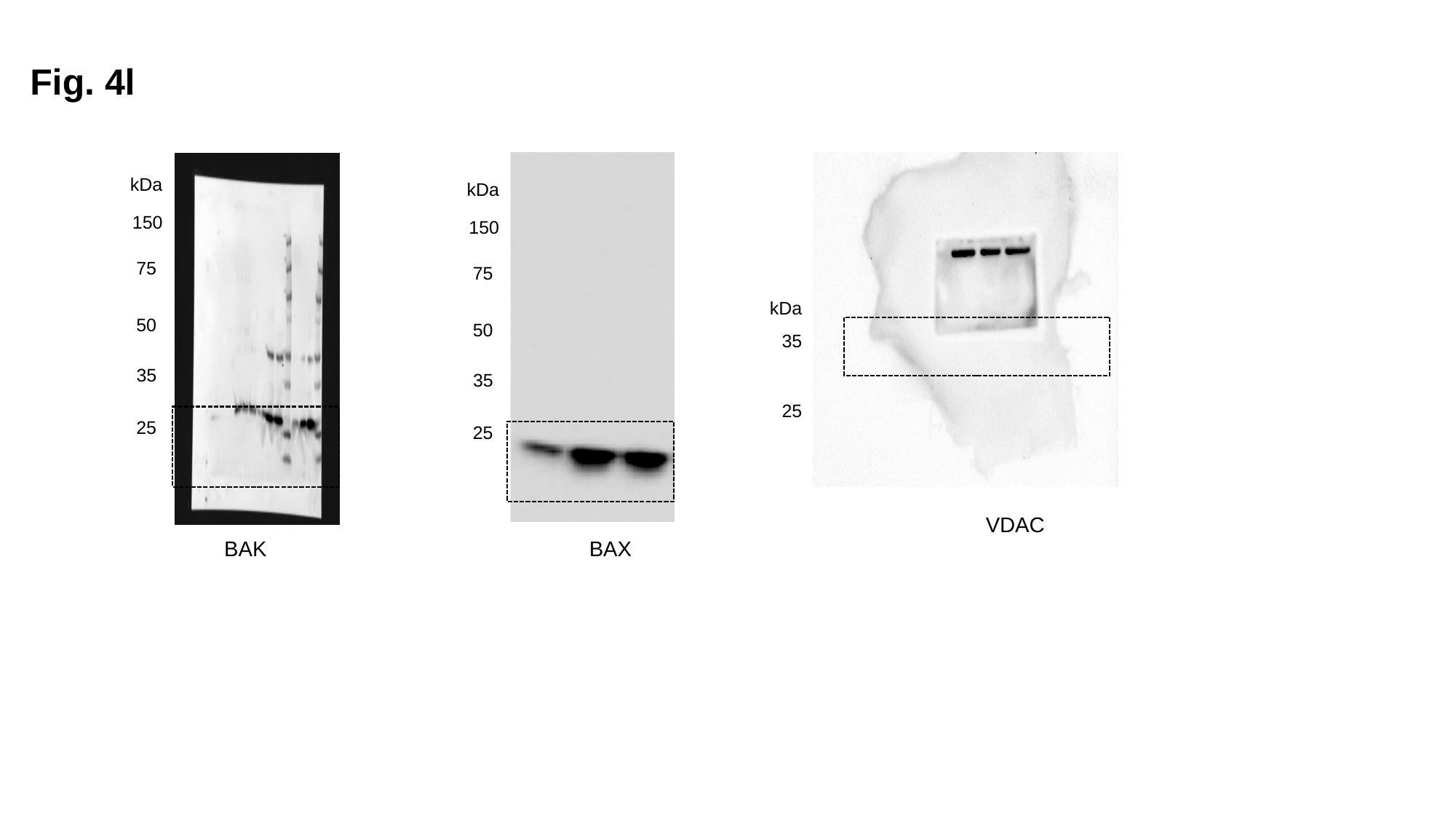

Fig. 4l
kDa
kDa
150
150
75
75
kDa
50
50
35
35
35
25
25
25
VDAC
BAX
BAK

## Slide 6
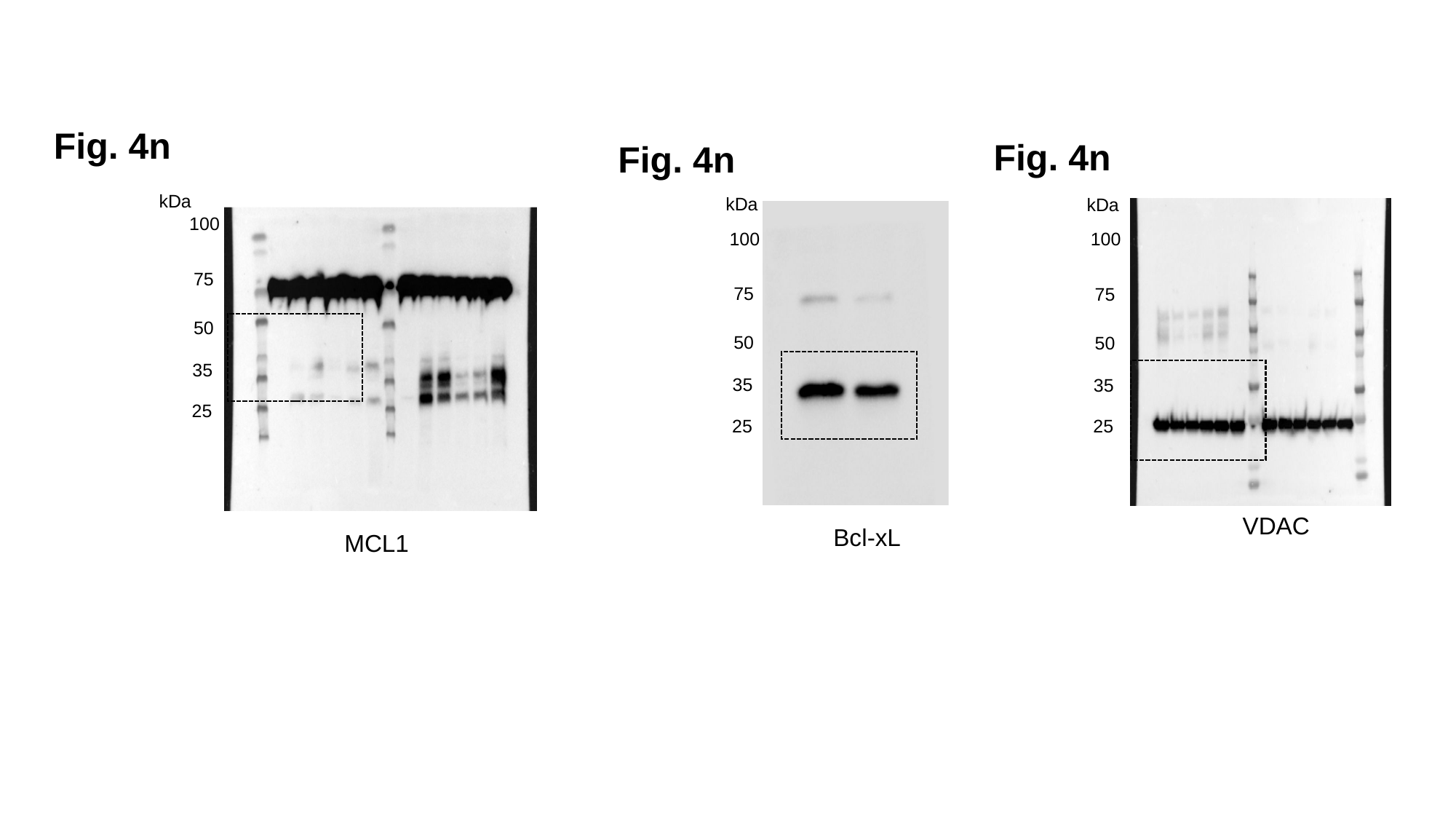

Fig. 4n
Fig. 4n
Fig. 4n
kDa
100
75
50
35
25
kDa
kDa
100
75
50
35
25
100
75
50
35
25
VDAC
Bcl-xL
MCL1

## Slide 7
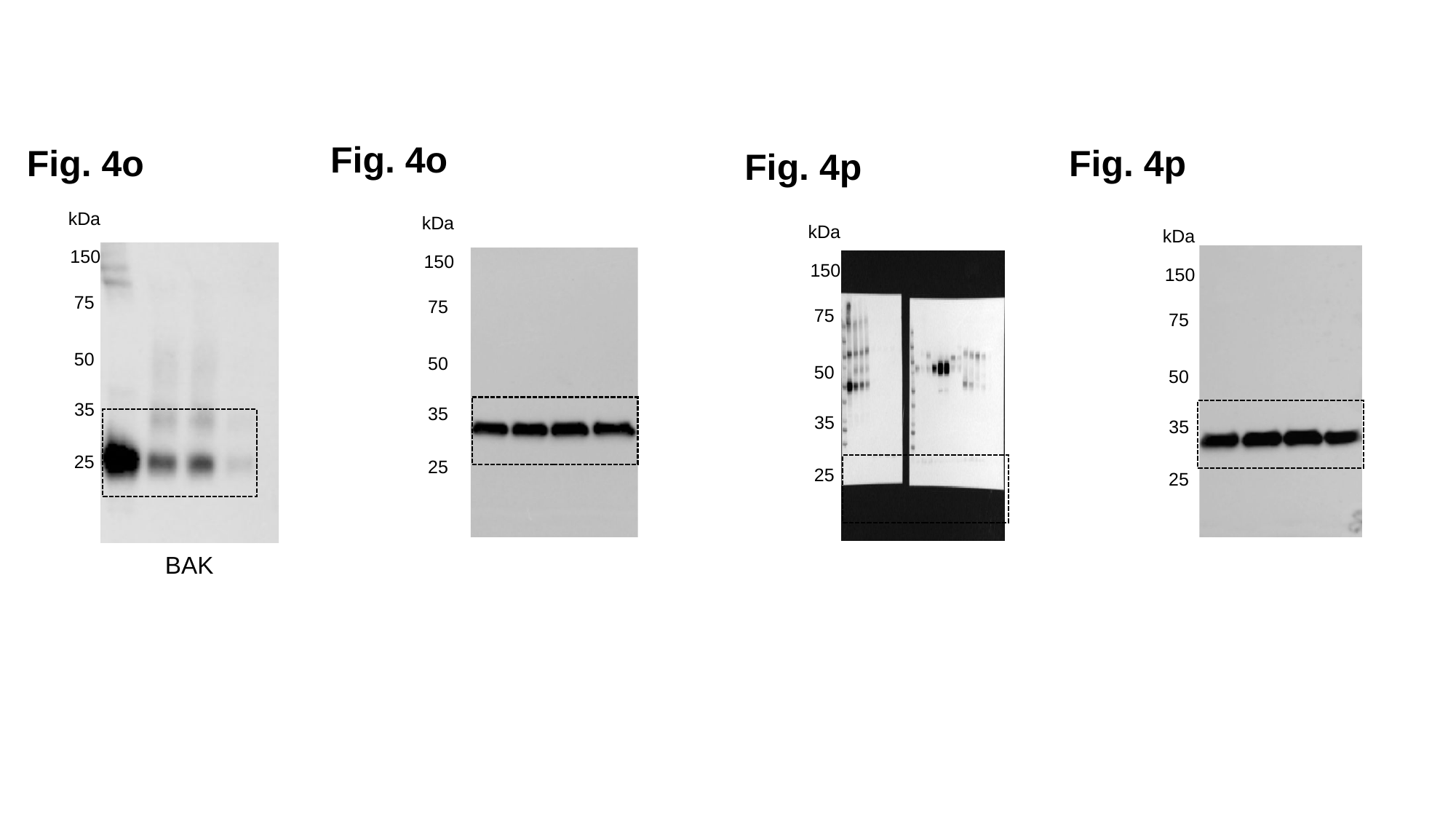

Fig. 4o
Fig. 4p
Fig. 4o
Fig. 4p
kDa
kDa
kDa
kDa
150
150
150
150
75
75
75
75
50
50
50
50
35
35
35
35
25
25
25
25
BAK

## Slide 8
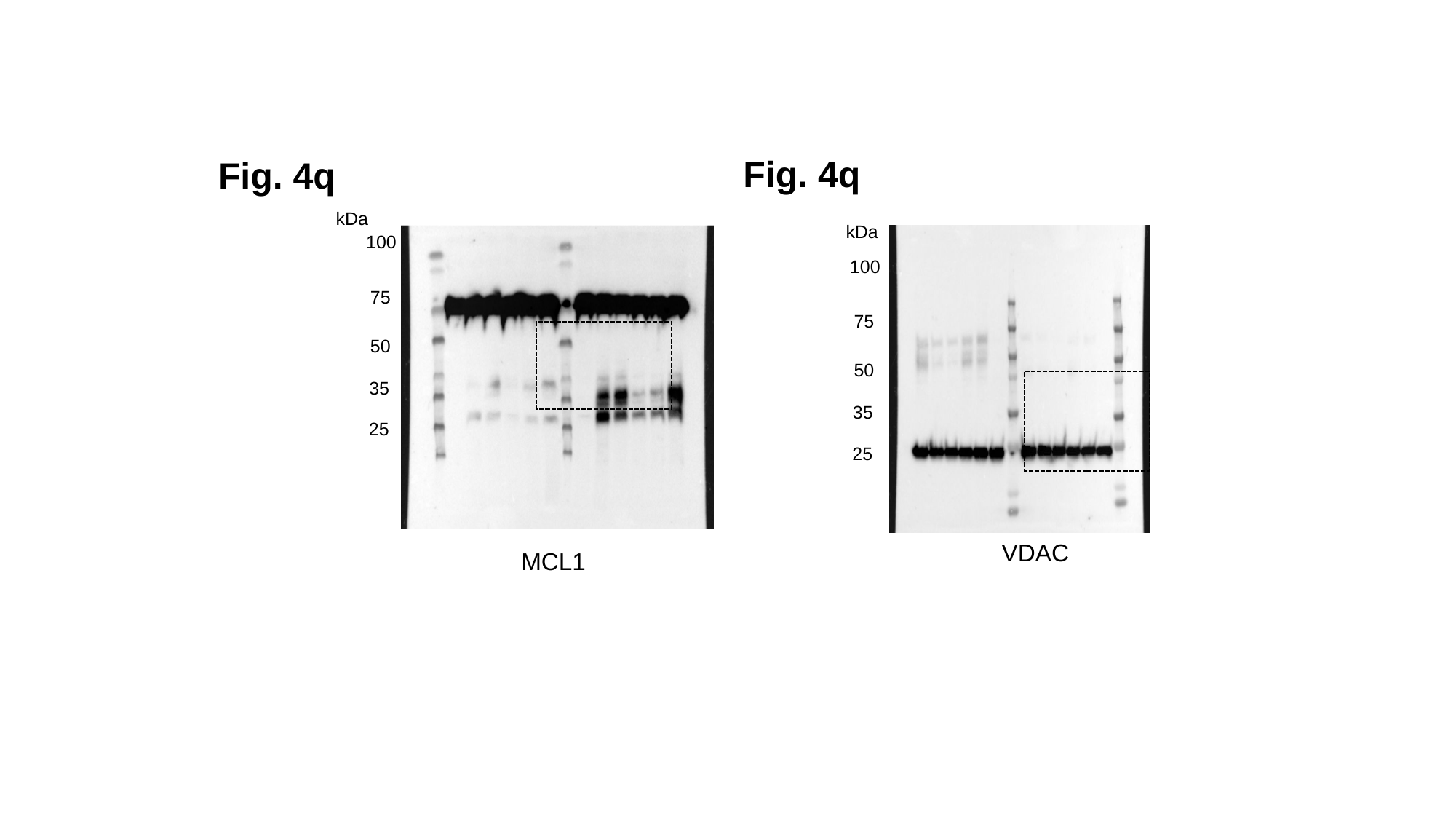

Fig. 4q
Fig. 4q
kDa
100
75
50
35
25
kDa
100
75
50
35
25
VDAC
MCL1

## Slide 9
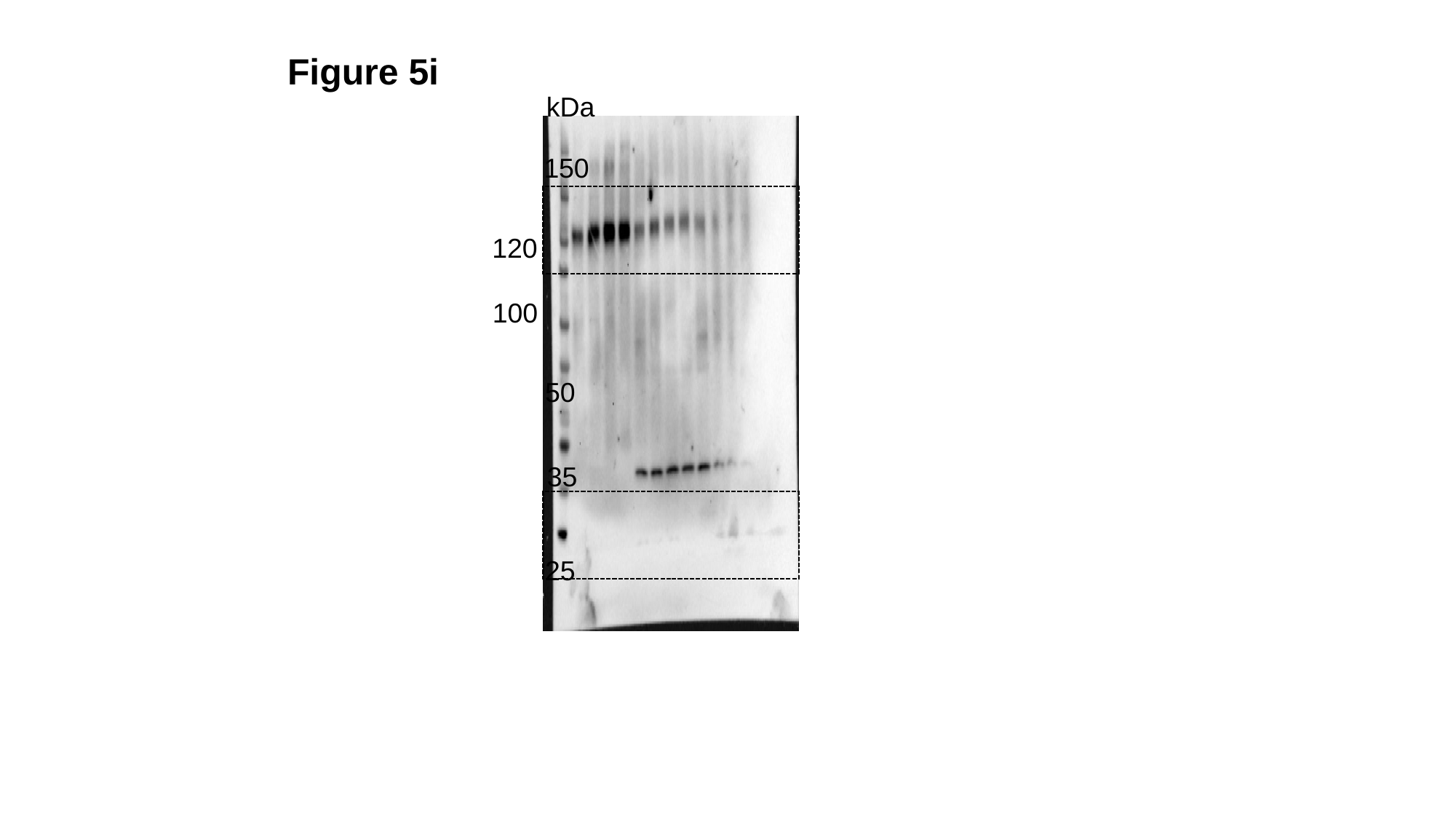

Figure 5i
kDa
150
50
35
25
120
100

## Slide 10
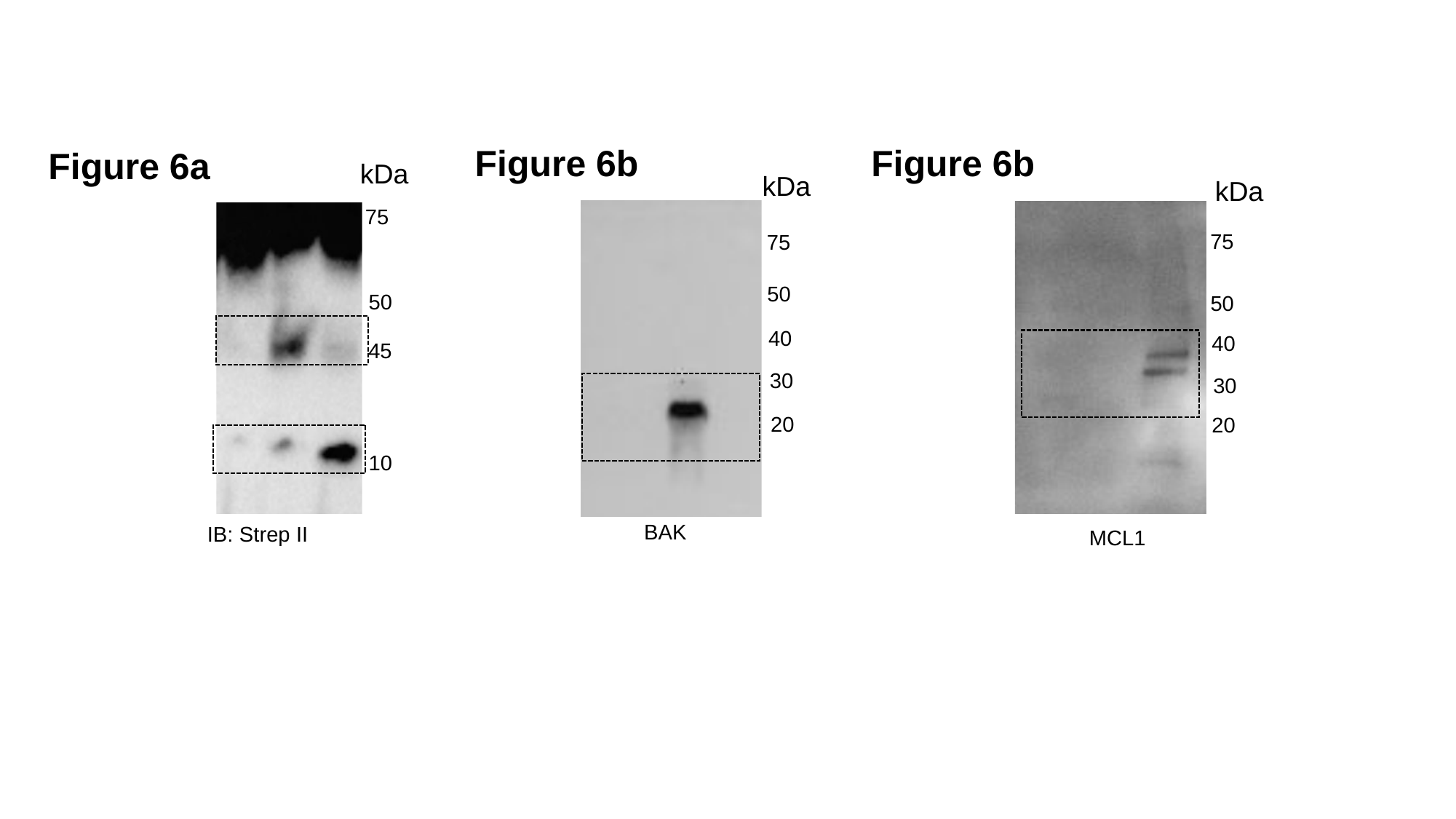

Figure 6b
Figure 6b
Figure 6a
kDa
kDa
kDa
75
 50
40
30
20
MCL1
75
75
 50
 50
40
 45
30
20
10
BAK
IB: Strep II

## Slide 11
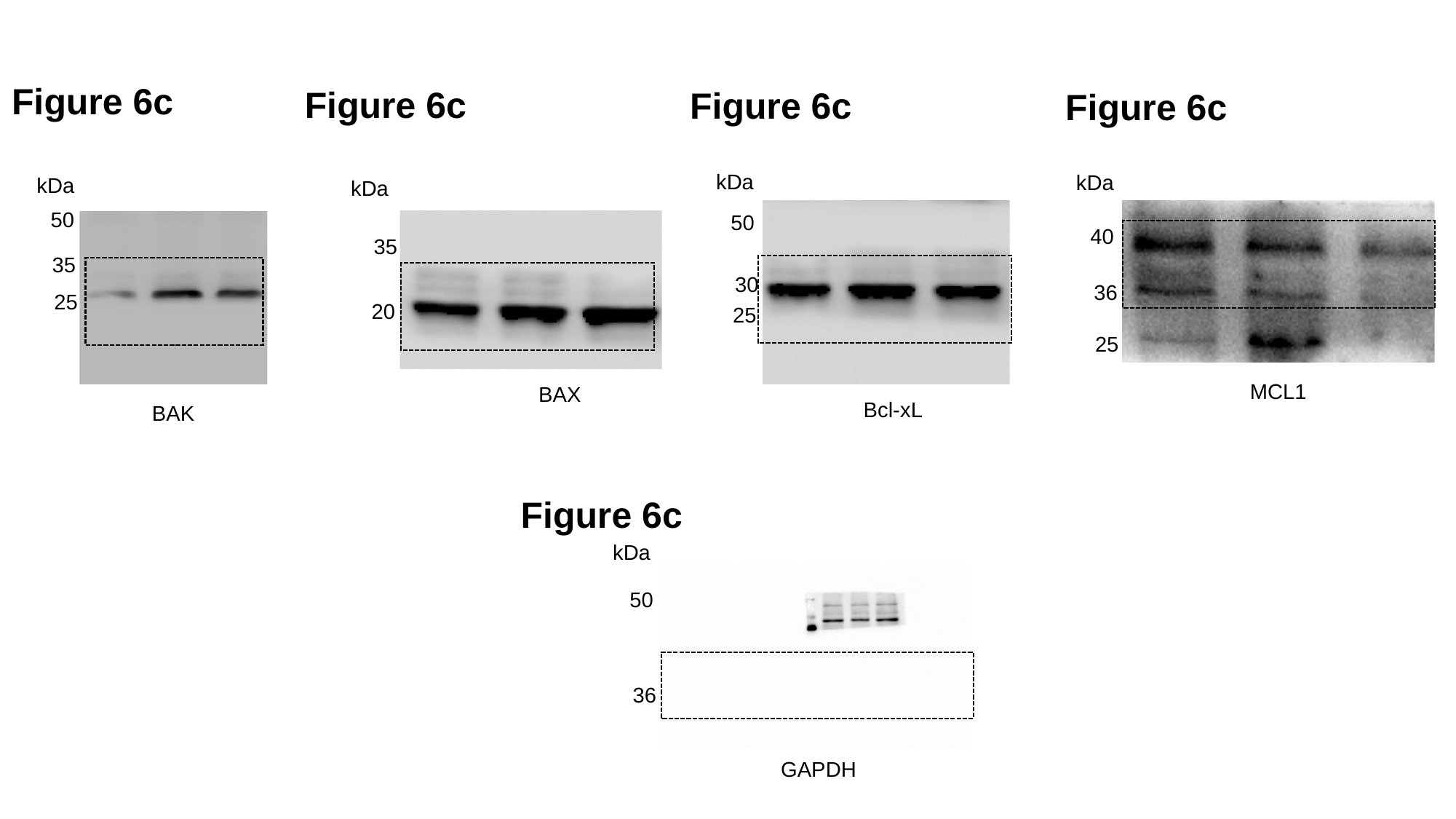

Figure 6c
Figure 6c
Figure 6c
Figure 6c
kDa
kDa
kDa
kDa
50
50
40
35
35
30
36
25
20
25
25
MCL1
BAX
Bcl-xL
BAK
Figure 6c
kDa
50
36
GAPDH

## Slide 12
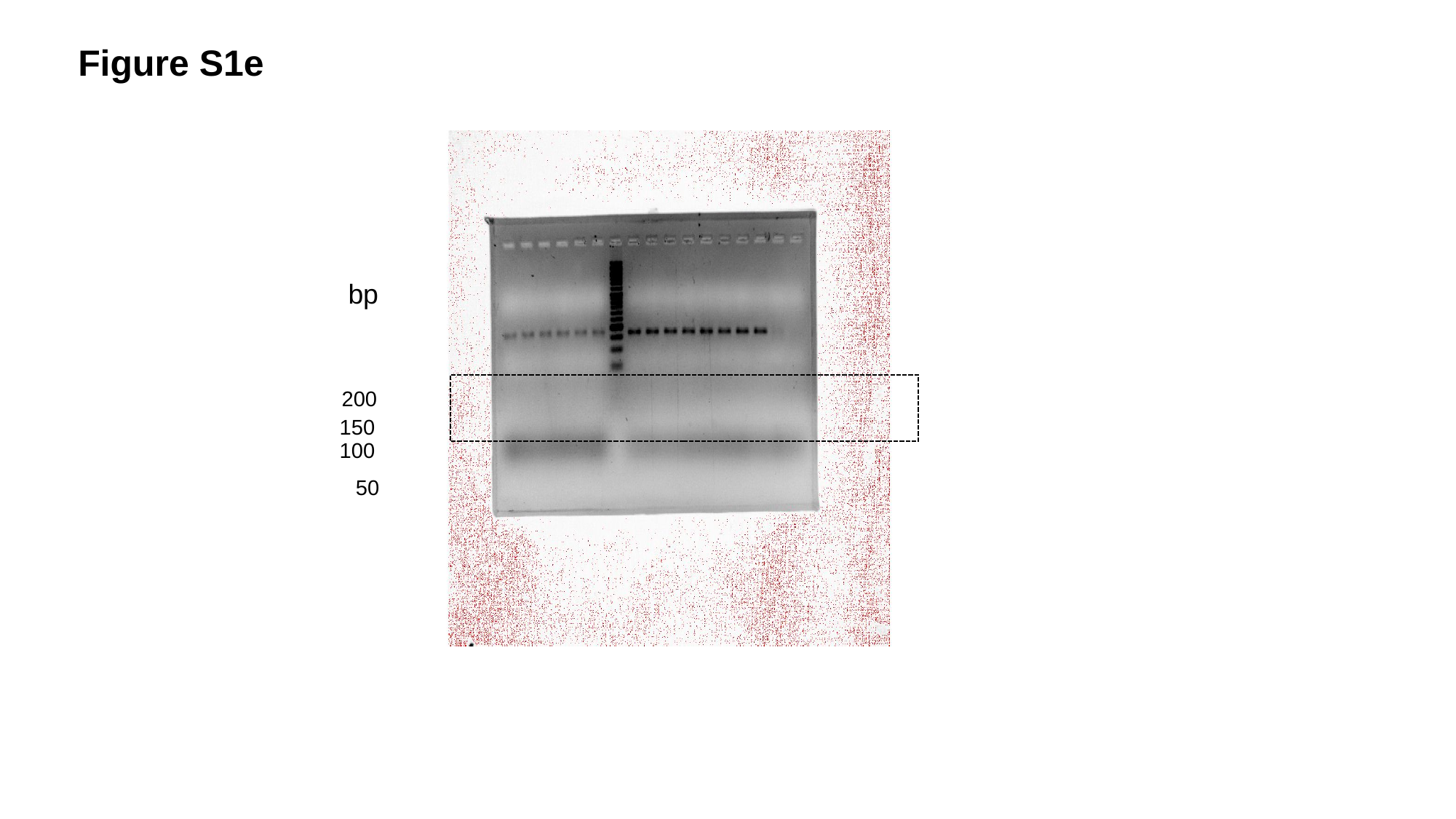

Figure S1e
bp
200
150
100
50

## Slide 13
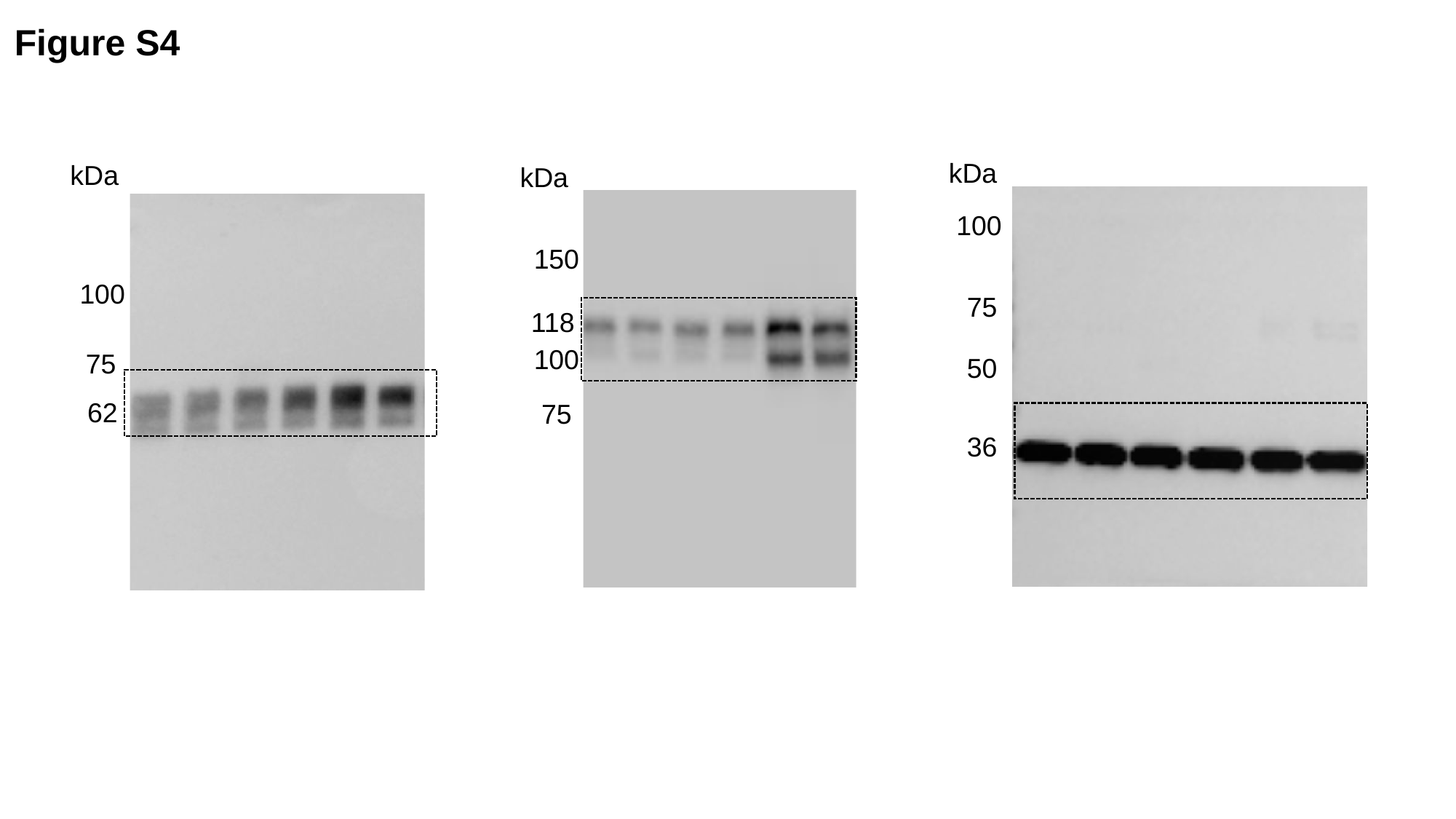

Figure S4
kDa
kDa
kDa
100
150
100
75
118
100
75
50
62
75
36
