# Supplementary material for: NSP4 and ORF9b of SARS-CoV-2 Induce Pro-Inflammatory Mitochondrial DNA Release in Inner Membrane-Derived Vesicles
Source: Cells. 2022 Sep 23;11(19):2969. doi: 10.3390/cells11192969 (PMC9561960; doi:10.3390/cells11192969)
Supplement: Supplementary file 1 [file cells-11-02969-s001.zip › Supplementary Figure legends 8-08-2022.pdf]

Figure legends

Figure S1:

COVID-19 patient plasma contains pro-inflammatory mtDNA

(a) Heat map showing RT-qPCR results of *S* gene to be absent in swab samples from patients suspected to be infected with omicron variant. The samples were positive for other SARS-CoV-2 genes like N and RNA-dependent RNA polymerase (RdRP) with RNAseP as internal control. PC indicates positive control, which are S gene positive samples **(b)** Relative mRNA expression of *IL1B* with respect to *ACTB* in NHBE cells incubated with plasma from COVID-19^{-ve} and COVID-19^{+ve} patients, respectively (n=8). **(c)** Representative flow cytometry histograms showing cell death in plasma treated NHBE cells, as observed with SYTOX green staining. Numbers indicate % cell death. **(d)** Representation of the flow cytometry data in *panel c* as % cell death. **(e)** Representative gel picture showing the expression of ND2 gene (representing mtDNA), as observed after performing PCR of ChIP samples. G1-G8 represents groups, with each group containing pooled plasma from 10 patients. **(f)** Relative mRNA expression of *IL6* with respect to *ACTB* in NHBE cells incubated with various concentrations of mtDNA (n=6). **(g)** Quantitative analysis of IL-6 in the total protein extracted from NHBE cells treated with increasing concentrations of mtDNA, as measured by ELISA. Data is represented in picograms/mL (n=6). **(h)** Representative flow cytometry histograms showing cell death in mtDNA treated NHBE cells, as observed with SYTOX green staining. Numbers indicate % cell death. **(i)** Representation of the flow cytometry data in *panel c* as % cell death (n=4). **(j)** Quantitative analysis of cytochrome c (Cyt c) in the plasma of COVID-19^{-VE} (20) and COVID-19^{+VE} (30) patients, as measured by ELISA. Data is represented in nanograms/mL. All data are represented as mean ± SEM of three independent experiments, analyzed by non-parametric *t* test using Graphpad Prism software. ****P* < 0.001; *****P* < 0.0001; ns: not significant.

Figure S2:

SARS-CoV-2 causes mitochondrial damage

(a) Representative LIGHTNING microscopy images showing changes in mitochondrial morphology, as visualized by anti-TOM20 staining in SARS-CoV-2 infected A549 cells. The insets of these images are shown in *Fig. 2e*. **(b)** Complete image panels of the representative images shown in *Fig. 2j.* **(c, d)** Representative images showing cGAS (green) and NLRP3 (red) expression in A549 cells infected with SARS-CoV-2. DAPI (blue) was used to label nuclei. **(e)** Quantitative analysis of images in panel *c* and *d* (n= 14). Data is represented as the integrated density of the signal of respective proteins. All data are represented as mean ± SEM of three independent experiments, analyzed by non-parametric *t* test using Graphpad Prism software. **P* < 0.05. Scale bars: a, b: 10 µm; c, d: 50 µm.

Figure S3

SARS-CoV-2 proteins NSP4 and ORF9b induce release of mtDNA

(a) Heat map showing the cytochrome c (Cyt c) levels associated with mitochondria when A549 cells were individually transfected with the 29 SARS-CoV-2 plasmids individually and stained with anti-Cyt c antibody. Images were taken 24 h post-transfection, followed by image analysis. A combination of ActD+ABT+zVD (2h) was used as positive control for mtDNA release. **(b)** Representative flow cytometry histograms showing measurement of mtROS and **(c)** $\Delta\Psi m$ by staining the cells with TMRE and mitoSOX, respectively. Antimycin C (AntC) was used as positive control for mtROS, and FCCP for $\Delta\Psi m$. **(d)** Representative images showing co-localization of picogreen stained mtDNA and mitotracker red stained mitochondria (MTR; pseudocolored as green), indicating mtDNA associated with mitochondria in A549 cells transduced with lentivirus encoded SARS-CoV-2 proteins as shown in *Fig. 3b.* Data is represented as mean ± SEM of three independent experiments. Scale bar: 10 µm.

Figure S4:

NSP4 and ORF9b of SARS-CoV-2 does not induce expression of cytosolic DNA sensors.

Representative immunoblot showing cGAS and NLRP3 expression in A549 cells transduced with N4+9b for 48h, followed by cell lysis and total protein extraction. Anti-cGAS and anti-NLRP3 antibodies were used to detect cGAS and NLRP3 respectively. A combination of ActD+ABT+zVD (2h) was used as positive control to induce the expression of these DNA sensors.

Figure S5

NSP4 and ORF9b lead to mitochondrial structural changes and macropore formation

(a) Representative images showing mitochondrial shape changes in cells transduced with VEC or N4+9b, as observed with mitotracker red (MTR; pseudocolored as green) with images analysis shown in *Fig. 4a*. Rotenone (Rot) was used as a positive control to induce mitochondrial shape changes. **(b)** Representative flow cytometry histograms showing cell death, as measured by SYTOX green staining. A549 cells were co-transduced with N4+9b with or without PINK1 lentiviral expression vector (PINK1^{pLV}). VEC1 represents the backbone construct for N4 and 9b plasmids, while VEC2 represents the backbone construct for PINK1 plasmid. Numbers indicate cell death percentage. **(c)** Cell death represented as % cell death analyzed from the histograms shown in *panel b* (n= 6). **(d)** Representative images showing BAK and **(e)** BAX (red) associated with anti-TOM20 stained mitochondria (green) in A549 cells transduced either with VEC or N4+9b. A combination of ActD+ABT+zVD (2h) was used as positive control. These images represent all the panels of images shown in *Fig.4h, j.* Data is represented as mean ± SEM of three independent experiments, analyzed by non-parametric *t* test, *$P < 0.05$; ***$P < 0.001$. Scale bars: 10 µm.

Figure S6

MCL1 downregulation and NSP4/ORF9b induce inner membrane disruption

(a) RT-qPCR data showing relative fold change expression of MCL1 upon transduction of A549 cells with Scramble (Srm) or shRNA targeting MCL1, followed by RNA extraction and RT-qPCR analysis (n= 6). **(b)** Representative images showing rupture of inner mitochondrial membrane of A549 cells transduced with N4+9b, stained with inner membrane targeted GFP (IMM^{GFP}) and anti-TOM20 to probe outer membrane. **(c)** Representative images showing of COX-IV expression in cells transduced with N4+9b, or **(d)** MCL1 shRNA, and stained with anti-TOM20. All data are represented as mean ± SEM of three independent experiments, analyzed by non-parametric *t* test, ****$P < 0.0001$. Scale bars: 10 µm.

Figure S7

BAK downregulation and MCL1 overexpression in MSCs increases intercellular mitochondrial transfer

(a) RT-qPCR data showing relative fold change expression of BAK upon transduction of A549 cells with Scramble (Srm) or shRNA targeting BAK. **(b)** Similarly, immunofluorescence was done followed by quantitive analysis of the images which indicate knockdown of BAK. (**c, d**) Lentiviral encoded VEC or MCL1 overexpression in MSCs ($MCL1^{pLV}$), followed by either RNA extraction and RT-qPCR analysis (n= 6). *(c)* or immunofluorescence analysis of the images *(d)*. **(e)** Representative flow cytometry histograms showing no change in the stem cell phenotype upon dual modification of MSCs by MCL1 overexpression and BAK knockdown by shRNA. **(f)** Representative images showing N4+9b transduced A549 stained with CTDR (red) and co-cultured with MSCs expressing mitoGFP (green). The images represent the quantitative data shown in *Fig7f*. **(g, h)** Quantitive analysis of the images by specifically measuring the fluorescence signal in A549 cells after co-culture with MSC^{Srm+V2} or $MSC^{shBAK+MCL1}$. The data represents the signal of BAK on mitochondria *(g)* or MCL1 on mitochondria *(h)*. A549 cells were transduced with vector or N4+9b before co-culture. All data are represented as mean ± SEM of three independent experiments, analyzed by non-parametric *t* test, ****$P < 0.0001$. Scale bar: 20 µm.

Figure S8

MSCs modulated with BAK shRNA and MCL1 overexpression plasmid attenuate cell death in A549 cells.

(a) Representative images showing cell death by fluorescent TUNEL assay (red) in A549 cells. SARS-CoV-2 infected cells were co-cultured with MSCs expressing either scramble vector (Srm) and vector (V2) or dual transduction of BAK-shRNA and MCL1 overexpression plasmid, and stained with cell tracker green (CTG), followed by TUNEL staining (red)**.** CTG stained dead cells appear yellow. **(b)** Quantitative analysis of TUNEL and CTG dual positive cells were counted and plotted as no. of A549 dead cells (n= 12). **(c)** Quantitative analysis of TUNEL and CTG dual positive cells done similar to *panel b* except that instead of SARS-CoV-2 infection, A549 cells were transduced with N4+9b (n= 14). **(d)** PCR analysis showing mtDNA copy number in the cell supernatant of co-culture of A549 cells and MSCs. A549 were transduced with N4+9b, whereas MSCs were modulated with BAK shRNA and MCL1 overexpression plasmid (n= 7). All data are

represented as mean ± SEM of three independent experiments, analyzed by non-parametric *t* test, ***$P < 0.001$; ****$P < 0.0001$. Scale bar: 100 μm.

Figure S9

Source data of all the blots.

The original full blots for the representative figures shown in Main and Supplementary Figures.

Supplementary Table

Sr No	Cell line	Company	Cat no
1	A549	ATCC	CCL-185
2	NHBE	LONZA	CC-2540S
3	Human MSCs	Apolo hospital	

Sr No	Primary Antibodies	Company	Identifier
1	Anti-TOMM20	Abcam	Cat# ab56783, RRID:AB_945896
2	Anti-VDAC1	Abcam	Cat# ab15895, RRID:AB_2214787
3	Anti-PINK1	Abcam	Cat# ab75487, RRID:AB_1310581
4	Anti-Cytochrome C	Abcam	Cat# ab90529, RRID:AB_10673869
5	Anti-Parkin	Cell Signaling Technology	Cat# 2132, RRID:AB_10693040
6	Anti-TFAM	Abcam	Cat# ab131607, RRID:AB_11154693
7	Anti-Bax (D2E11)	Cell Signaling Technology	Cat# 5023, RRID:AB_10557411
8	Anti-Bak (D4E4)	Cell Signaling Technology	Cat# 12105, RRID:AB_2716685
9	Anti-Mcl-1 (D2W9E)	Cell Signaling Technology	Cat# 94296, RRID:AB_2722740
10	Anti-COX IV (3E11)	Cell Signaling Technology	Cat# 4850, RRID:AB_2085424
11	Anti-GFP	Abcam	Cat# ab290, RRID:AB_303395
12	Anti-Bcl-xL (54H6)	Cell Signaling Technology	Cat# 2764, RRID:AB_2228008
13	Anti-GAPDH	Santa Cruz Biotechnology	Cat# sc-32233, RRID:AB_627679
14	Anti-E-cadherin	Santa Cruz Biotechnology	Cat# sc-8426, RRID:AB_626780
15	Anti-Strep-tag II antibody	Abcam	Cat# ab76949, RRID:AB_1524455
16	Anti-Spike (SARS CoV-2)	CST	Cat# 52342, RRID: Not Available
17	IgG	Abcam	Cat# ab172730, RRID: 2687931
18	Anti-DNA	Abcam	Cat# ab273137, RRID: Not available
19	TFAM (D5C8) Rabbit	CST	Cat #8076, RRID: AB_10949110

Sr No	Commercial assay Kits	Source	Catalog number
1	Live and Dead Cell Assay	Abcam	Cat# ab115347
2	IL-1 beta Human Elisa Kit	R&D Systems	Cat # DLB50
3	Immunoprecipitation kit	Abcam	Cat # ab206996
4	SYTOX	Invitrogen	Cat # R37168
5	DeadEnd™ Colorimetric TUNEL System	Promega	G7130
6	Mitochondria Isolation Kit	Sigma	MITOISO2-1KT
7	QIAamp Viral RNA Mini Kit (50)	Qiagen	Cat. No. / ID: 52904

Sr No	Plasmid name	Source	Addgene no
1	pWPI-IRES-Puro-Ak-ACE2-TMPRSS2	Addgene	RRID:Addgene_154987
2	pcDNA3 TFAM-mClover	Addgene	RRID:Addgene_129574
3	pLYS1-FLAG-MitoGFP-HA	Addgene	RRID:Addgene_50057
4	pCMV-VSV-G	Addgene	RRID:Addgene_8454
5	pCMV-dR8.2 dvpr	Addgene	RRID:Addgene_8455
6	pLVX-EF1alpha-nCoV2019-nsp1-2xStrep-IRES-Puro	Addgene	RRID:Addgene_141367
7	pLVX-EF1alpha-nCoV2019-nsp2-2xStrep-IRES-Puro	Addgene	RRID:Addgene_141368
8	pLVX-EF1alpha-nCoV2019-nsp4-2xStrep-IRES-Puro	Addgene	RRID:Addgene_141369
9	pLVX-EF1alpha-nCoV2019-nsp5-2xStrep-IRES-Puro	Addgene	RRID:Addgene_141370
10	pLVX-EF1alpha-nCoV2019-nsp6-2xStrep-IRES-Puro	Addgene	RRID:Addgene_141372
11	pLVX-EF1alpha-nCoV2019-nsp7-2xStrep-IRES-Puro	Addgene	RRID:Addgene_141373
12	pLVX-EF1alpha-nCoV2019-nsp8-2xStrep-IRES-Puro	Addgene	RRID:Addgene_141374
13	pLVX-EF1alpha-nCoV2019-nsp9-2xStrep-IRES-Puro	Addgene	RRID:Addgene_141375
14	pLVX-EF1alpha-nCoV2019-nsp10-2xStrep-IRES-Puro	Addgene	RRID:Addgene_141376
15	pLVX-EF1alpha-nCoV2019-nsp11-2xStrep-IRES-Puro	Addgene	RRID:Addgene_141377
16	pLVX-EF1alpha-nCoV2019-nsp12-2xStrep-IRES-Puro	Addgene	RRID:Addgene_141378
17	pLVX-EF1alpha-nCoV2019-nsp13-2xStrep-IRES-Puro	Addgene	RRID:Addgene_141379
18	pLVX-EF1alpha-2xStrep-nCoV2019-nsp14--IRES-Puro	Addgene	RRID:Addgene_141380
19	pLVX-EF1alpha-nCoV2019-nsp15-2xStrep-IRES-Puro.dna	Addgene	RRID:Addgene_141381
20	pTwist-EF1alpha-nCoV-2019-S-2xStrep	Addgene	RRID: Addgene_141382
21	pLVX-EF1alpha-nCoV2019-orf3a-IRES-Puro	Addgene	RRID:Addgene_141383
22	pLVX-EF1alpha-2xStrep-nCoV2019-orf3b-IRES-Puro	Addgene	RRID:Addgene_141384
23	pLVX-EF1alpha-nCoV2019-E-IRES-Puro	Addgene	RRID:Addgene_141385
24	pLVX-EF1alpha-nCoV2019-M-IRES-Puro	Addgene	RRID:Addgene_141386
25	pLVX-EF1alpha-nCoV2019-orf6-IRES-Puro	Addgene	RRID:Addgene_141387
26	pLVX-EF1alpha-nCoV2019-orf7a-IRES-Puro	Addgene	RRID:Addgene_141388
27	pLVX-EF1alpha-2xStrep-nCoV2019-orf7b-IRES-Puro	Addgene	RRID:Addgene_141389
28	pLVX-EF1alpha-nCoV2019-orf8-IRES-Puro	Addgene	RRID:Addgene_141390
29	pLVX-EF1alpha-nCoV2019-N-IRES-Puro	Addgene	RRID:Addgene_141391
30	pLVX-EF1alpha-nCoV2019-orf9b-IRES-Puro	Addgene	RRID:Addgene_141392
31	pLVX-EF1alpha-2xStrep-nCoV2019-orf9c-IRES-Puro	Addgene	RRID:Addgene_141393
32	pLVX-EF1alpha-nCoV2019-orf10-IRES-Puro	Addgene	RRID:Addgene_141394
33	pDONR207 SARS-CoV-2 NSP3	Addgene	RRID:Addgene_141257
34	pDONR223 SARS-CoV-2 NSP16	Addgene	RRID:Addgene_141269
35	EMT vector 1 for SARS CoV 2	In this paper	
36	EMT vector 2 for SARS CoV 2	In this paper	
37	VEH 1 for MCL-1	In this paper	
38	VEH 2 for MCL-1	In this paper	
39	PINK1 pLV	In this paper	

40	N4 T492I (Omicron variant)	In this paper
41	TFAM shRNA	Ahmad et al, 2014
42	BCL-xL	In this paper
43	Scrambled shRNA (Srm)<br>pLK0.1 vector backbone	In this paper

Sr No	Drugs/Serum/Reagents/Media	Company	Catalog no
1	ABT-737	Calbiochem	197333
2	Actinomycin D	Calbiochem	114666
3	Z-VAD-FMK	Sigma-Aldrich	V116
4	Normal Goat Serum	Jackson	005-000-121
5	Antimycin A	Sigma	A8674
6	Rotenone	Sigma	R8875
7	Carbonyl cyanide 4-(trifluoromethoxy)phenylhydrazone (FCCP)	Sigma	C2920
8	LTX	Invitrogen	A12621
9	Lipofectamin 3000	Invitrogen	L3000008
10	Ionomycin	Sigma	I0634
11	Calcein AM	Invitrogen	C1430
12	Triton X 100	Sigma	T9284
13	KAPA SYBER FAST QPCR MM	KAPA	kk4601
`14	Bovine Serum Albumin (BSA)	Bio World	22070004-5
15	StemPro™ MSC SFM	Gibco	A1033201
16	Ham's Nutrient Mixture F12	Sigma	51651C
17	SuperSignal™ West Femto Maximum Sensitivity Substrate	Invitrogen	34095
18	SuperSignal™ West Pico PLUS Chemiluminescent Substrate	Invitrogen	34580
19	L-Glutamine (200 mM)	Invitrogen	25030081
20	PageRuler™ Prestained Protein Ladder	Invitrogen	26616
21	Ampicillin sodium salt	Himedia	CMS645-5G
22	Protease Inhibitor Cocktail	Sigma	p8340
23	Fetal Bovine Serum	Gibco	10270106
24	Pierce™ 16% Formaldehyde	Invitrogen	28908
25	Hexadimethrine bromide	Sigma	H9268-10G
26	Bicinchoninic Acid (BCA)	Sigma	B9643-1L
27	Penicillin-Streptomycin	Invitrogen	15140122
29	DMEM, high glucose	Gibco	11965092
30	Sodium Pyruvate	Sigma	S8636-100ML
31	Opti-MEM	Gibco	22600050
32	BEGM™-2 BulletKit Medium	Lonza	CC-3170
33	2X PCR Master Mix	Thermo Fisher Scientific	K0171
34	amaR OnePCR	GeneDireX, Inc	SM213-0250
35	PE Mouse Anti-Human CD73	BD Biosciences	550257

36	Anti-Human CD45 FITC/CD34 PE	BD Biosciences	341071
37	PE-Cy™5 Mouse Anti-Human CD90	BD Biosciences	555597
38	CD44 Monoclonal Antibody (IM7), PE	Thermo fisher/ebioscience	12-0441-82

Sr No	Dyes	Company	Catalog no
1	TMRE	Invitrogen	T669
2	MitoSOX Red	Invitrogen	M36008
3	Mitotracker Red	Invitrogen	M7513
4	Mitotracker Green	Invitrogen	M7514
5	DAPI	Invitrogen	P36966
6	PicoGreen	Invitrogen	P11496
7	CTDR	Invitrogen	C34565

Sr No	Software and algorithms	Source	Ref
1	ImageJ	(Schneider et al., 2012)	https://imagej.nih.gov/ij; RRID:SCR_003070
2	Prism v7	GraphPad Software	https://www.graphpad.com/scientific-software/prism/; RRID:SCR_002798
3	Imaris v8.0.2	Bitplane	http://www.bitplane.com/; RRID:SCR_007370
4	FlowJo v10	FlowJo, Treestar Inc.	https://www.flowjo.com; RRID:SCR_008520

Sr No	sh RNA	Sequences	Ref
1	MCL1 shRNA-1	5’GCAAGAGGATTATGGCTAA3’	In this paper
2	MCL1 shRNA-2	5’GTTGATGAATGGAAATTCTTTCACT3’	In this paper
3	MCL1 shRNA-3	5’CCAGTATACTTCTTAGAAAGTCCAA3’	In this paper
4	BAK shRNA1	5’CCCATTCACTACAGGTGAA3’	PMID: 34385422
5	BAK shRNA2	5’ATGGTCACCTTACCTCTGCAACC3’	PMID: 34385422
6	BAK shRNA3	5’GGTACGAAGATTCTTC AAATCAT3’	PMID: 34385422
7	BAX shRNA1	5’GCTCTGAGCAGATCATGAA 3’	PMID: 34385422
8	BAX shRNA2	5’CAGCTCTGAGCAGATCATGAAGA3’	In this paper
9	BAX shRNA3	5’GACATGTTTTCTGACGGCAACTT3’	In this paper

Sr No	Gene name	Forward primer sequence	Reverse primer sequence	ref/ID
4	mt-tRNA	CACCCAAGAACAGGGTTTGT	TGGCCATGGGTATGTTGTTAA	(1)
5	Nuclear B2M	TGCTGTCTCCATGTTTGATGTATCT	TCTCTGCTCCCCACCTCTAAGT	In this paper
6	ND1	CGATTCCGCTACGACCAACT	AGGTTTGAGGGGGAATGCTG	In this paper
7	ND2	AGCACCACGACCCTACTACT	TGGTGGGGATGATGAGGCTA	In this paper
8	ND3	GCGGCTTCGACCCTATATCC	AGGGCTCATGGTAGGGGTAA	(2)
9	ND4	TCGCTCACACCTCATATCCTC	AGGCGGCAAAGACTAGTATGG	(2)

10	ND4	CTCACTCTCACTGCCCAAGA	AGCCGCCTAGTTTCAAGAGT	In this paper
11	ND5	CACATCTGTACCCACGCCTT	AATGCTAGGCTGCCAATGGT	In this paper
12	ND6	CCTATTCCCCCGAGCAATCTC	GGAGGATCCTATTGGTGCGG	In this paper
13	Cytochrome c	GGGAGGCAAGCATAAGACTG	TATCCTCTCCCCAGGTGATG	In this paper
14	COX-IV	ACTACCCCTTGCCTGATGTG	GCCCACAACTGTCTTCCATT	In this paper
16	IFNB1	CCAGGGGAAAACTCATGAGC GGTGCTGTGGGTGAAAGAGT	AGCAATTGTCCAGTCCCAGA	In this paper
18	MCL-1	AAGGCATGCTTCGGAAACTG	TGCTAATGGTTCGATGCAGC	In this paper
19	BAX	GACTATCATATGCTTACCGTAACTTG	GGTCGAGAATTCAAAAAGCTCTG	In this paper
20	BAK	GACTATCATATGCTTACCGTAAC	GGTCGAGAATTCAAAAACCC	In this paper

**Synthetic fragment of ND-2**
AGCACCACGACCCTACTACTATCTCGCACCTGAAACAAGCTAACATGACTAACACCCTTAATTCCATCCACCCTCC TCTCCCTAGGAGGCCTGCCCCCGCTAACCGGCTTTTTGCCCAAATGGGCCATTATCGAAGAATTCACAAAAAACAA TAGCCTCATCATCCCCACCA

1. Lin, C.S., H.T. Lee, M.H. Lee, S.C. Pan, C.Y. Ke, A.W. Chiu, and Y.H. Wei. 2016. Role of Mitochondrial DNA Copy Number Alteration in Human Renal Cell Carcinoma. *Int J Mol Sci*. 17.

2. Liu, Z., J. Tian, F. Peng, and J. Wang. 2022. Hypermethylation of mitochondrial DNA facilitates bone metastasis of renal cell carcinoma. *J Cancer*. 13:304-312.
