# Supplementary figures and images for: NSP4 and ORF9b of SARS-CoV-2 Induce Pro-Inflammatory Mitochondrial DNA Release in Inner Membrane-Derived Vesicles
Source: Cells. 2022 Sep 23;11(19):2969. doi: 10.3390/cells11192969 (PMC9561960; doi:10.3390/cells11192969)

## Slide 1
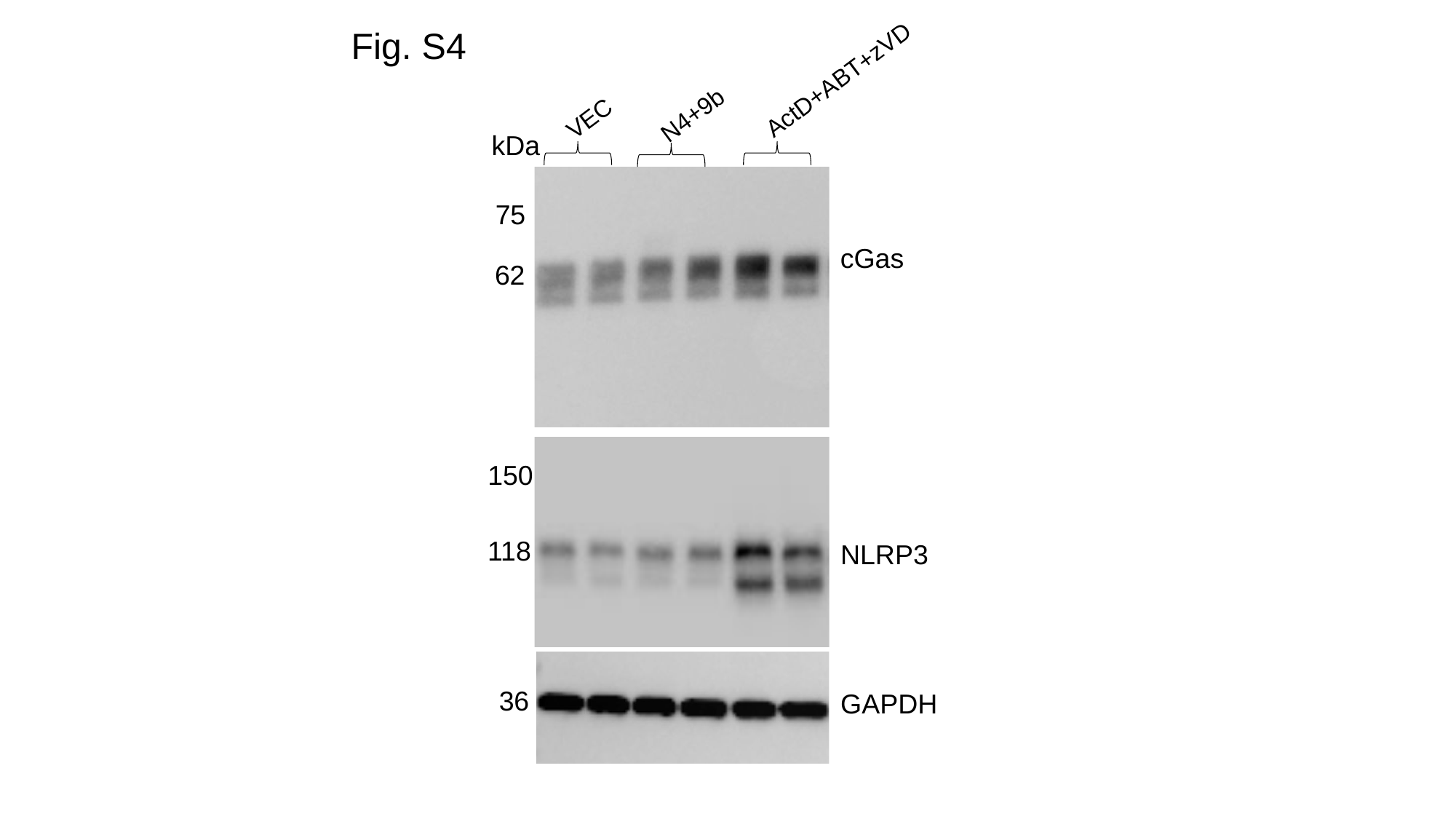

Fig. S4
ActD+ABT+zVD
N4+9b
VEC
kDa
75
cGas
62
150
118
NLRP3
36
GAPDH

Supplement: Supplementary file 1 [file cells-11-02969-s001.zip › Figure S4.pptx]

## Slide 1
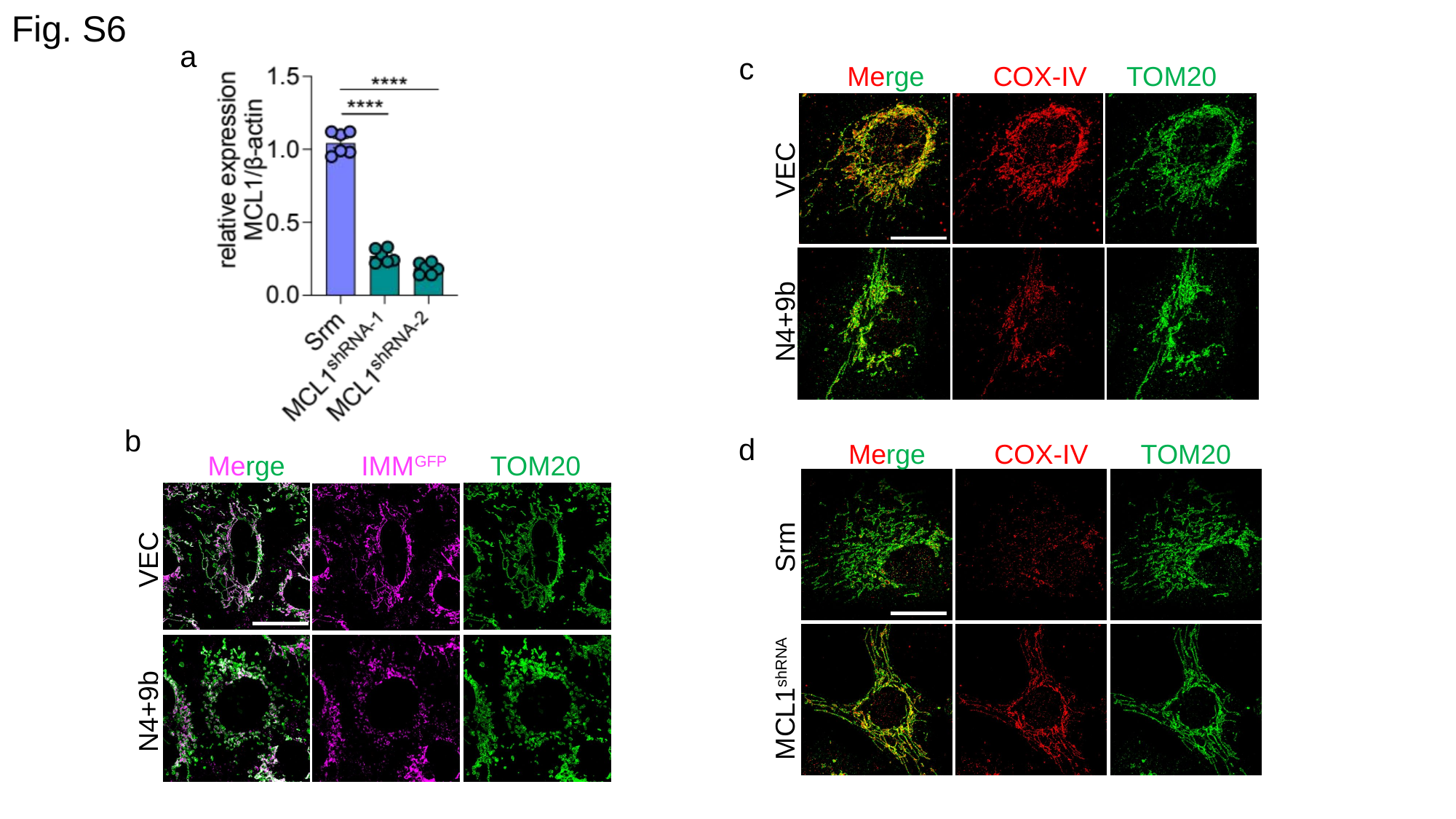

Fig. S6
a
c
Merge COX-IV TOM20
VEC
N4+9b
b
d
Merge COX-IV TOM20
Srm
MCL1shRNA
Merge IMMGFP TOM20
VEC
N4+9b

Supplement: Supplementary file 1 [file cells-11-02969-s001.zip › Figure S6.pptx]

## Slide 1
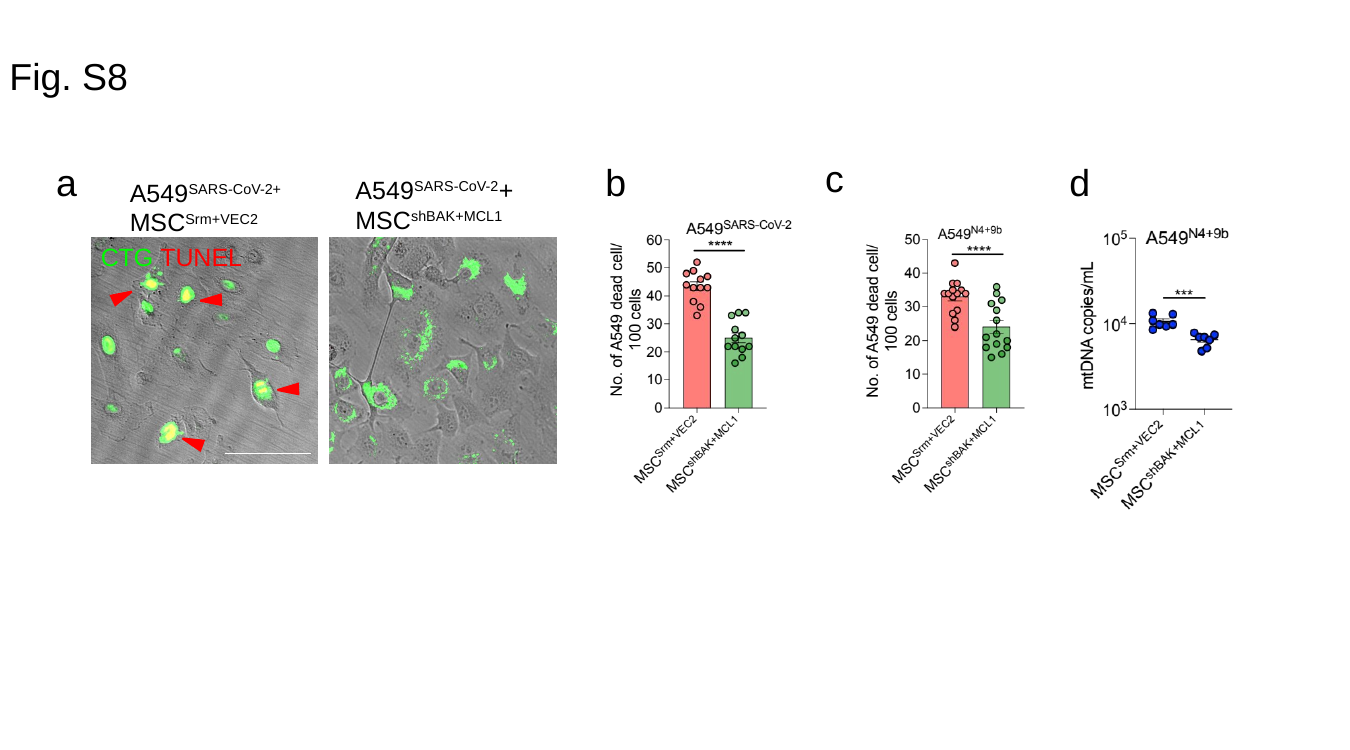

Fig. S8
c
a
b
d
A549SARS-CoV-2+
MSCshBAK+MCL1
A549SARS-CoV-2+
MSCSrm+VEC2
CTG TUNEL

Supplement: Supplementary file 1 [file cells-11-02969-s001.zip › Figure S8.pptx]
